# Genome-wide identification of the context-dependent sRNA expression in *Mycobacterium tuberculosis*

**DOI:** 10.1186/s12864-020-6573-5

**Published:** 2020-02-18

**Authors:** Vimla Kany G. Ami, Rami Balasubramanian, Shubhada R. Hegde

**Affiliations:** Institute of Bioinformatics and Applied Biotechnology (IBAB), Bengaluru, 560 100 India

**Keywords:** sRNA, RNA-Seq, Tuberculosis, Gene regulation, Persistence

## Abstract

**Background:**

Tuberculosis remains one of the leading causes of morbidity and mortality worldwide. Therefore, understanding the pathophysiology of *Mycobacterium tuberculosis* is imperative for developing new drugs. Post-transcriptional regulation plays a significant role in microbial adaptation to different growth conditions. While the proteins associated with gene expression regulation have been extensively studied in the pathogenic strain *M. tuberculosis* H37Rv, post-transcriptional regulation involving small RNAs (sRNAs) remains poorly understood.

**Results:**

We developed a novel moving-window based approach to detect sRNA expression using RNA-Seq data. Overlaying ChIP-seq data of RNAP (RNA Polymerase) and NusA suggest that these putative sRNA coding regions are significantly bound by the transcription machinery. Besides capturing many experimentally validated sRNAs, we observe the context-dependent expression of novel sRNAs in the intergenic regions of *M. tuberculosis* genome. For example, ncRv11806 shows expression only in the stationary phase, suggesting its role in mycobacterial latency which is a key attribute to long term pathogenicity. Also, ncRv11875C showed expression in the iron-limited condition, which is prevalent inside the macrophages of the host cells.

**Conclusion:**

The systems level analysis of sRNA highlights the condition-specific expression of sRNAs which might enable the pathogen survival by rewiring regulatory circuits.

## Background

Tuberculosis remains one of the most successful human pathogens and one among the top 10 leading causes of morbidity and mortality from infectious diseases worldwide [1]⁠. In about 90% of the affected individuals, bacteria may persist in the form of an asymptomatic latent infection, which may reactivate under any form of immunosuppression [2]. During the course of infection, M. tuberculosis adapts to different micro-environments such as iron restriction, starvation, hypoxia and low pH. The transcriptional and translational machinery associated with bacterial adaptation in response to environmental changes have been widely studied in *M. tuberculosis* [3]. Most of these studies have invariably focused on the protein-coding regions of the genome. However, with the emergence of transcriptome sequencing revolution, expression patterns in the non-protein coding regions can be analysed meaningfully. This is important as the recent studies suggest that bacterial genomes code for small non-coding RNAs (sRNAs), which play a significant role in modulating translation or mRNA stability [4].

Depending on the base pairing with their target mRNAs, there are three broad classes of sRNAs (i) *Antisense sRNAs* which are present in the opposite strand of their target mRNA and share an extensive sequence complementarity, (ii) *Trans-encoded sRNAs* which are located largely in the intergenic regions (IGRs) and share limited sequence complementarity with their target mRNAs, and (iii) *Cis-encoded sRNAs* which are present in the untranslated regions (UTRs) of the genes [4–6]. These sRNAs are important for adapting to various stresses and environmental changes caused by the host defense mechanisms. For example, *Escherichia coli* sRNA RyhB maintains iron homeostasis by downregulating the expression of iron utilization proteins such as SodB, PflA and MsrB [7]. Also in *E. coli*, sRNAs DsrA and RprA base-pair with the mRNA of the stress response sigma factor *rpoS*, thereby increasing its stability by rendering protection from RNaseE [8]⁠. While adapting to less carbon source, sRNA CrcZ in *Pseudomonas aeruginosa* helps in relieving catabolite repression by sequestering Crc protein [9]. Some of the sRNAs identified in *M. tuberculosis* so far include F6, B11, MTS2823, ncRV12659, DrrS, Mcr7 and MrsI [10–15]⁠. DrrS showed increased expression in the stationary phase which is regulated by the *M. tuberculosis* dormancy regulator DosR [12]⁠. Also, ncRv12659 which is transcribed anti sense to the gene *Rv2660c,* was shown to be expressed during starvation [15]. Another condition-specific sRNA is MrsI which is expressed during iron starvation, oxidative stress and membrane stress [14].

Both sequence and expression based approaches have been used to identify sRNAs in the bacterial genomes. Previously in *E. coli*, putative sRNAs were shortlisted by identifying conserved IGRs across *Salmonella typhi*, *S. paratyphi*, and *S. Typhimurium,* with potential transcription start and termination sites [16]⁠. In another sequence-based approach, 13 well annotated bacterial species were considered for identifying conserved IGRs which are likely to code for sRNAs [17]. However, such sequence-based methods remain efficient in identifying only those sRNAs which are conserved across other bacterial genomes. The emergence of transcriptome data has opened a way to identify sRNAs based on their expression. In *E. coli,* IGRs were identified as sRNAs if they showed significant expression compared to the upstream and downstream protein-coding genes [18]⁠. In *M. tuberculosis*, expression data corresponding to the log-phase growth was utilised to identify sRNAs by considering the read depth at a given position in the genome excluding the UTRs [19]⁠. However, none of these genome-wide analyses focused on the conditional expression of the sRNAs in different stress environments.

While sequence conservation-based approaches fail to identify species specific sRNAs and sRNAs which show significant divergence, expression based methods need to be improved to address the following challenges: a) to detect sRNA expression independent of the neighbouring gene expression and the signals arising due to UTR expression, b) to identify sRNA expression within an IGR without normalising the reads across the entire IGR, and c) to identify condition-specific expression of the sRNAs.

We used RNA-Seq data to identify sRNAs in *M. tuberculosis.* Our method employs sliding-windows along the IGRs to detect sRNA expression, while efficiently dismissing the expression signals arising from the upstream and the downstream genes and their UTRs. Previously, such moving windows of normalised RNA-Seq values along the genome were used to detect sigma-H dependent promoters in *Listeria monocytogenes* [20]. Also, as sRNAs are recognised to tune the cellular responses, we profiled the conditional expression of sRNAs by analysing the expression data across multiple growth conditions. Analysis of these condition specific sRNAs along with their predicted targets provided insights on the putative regulatory mechanisms for bacterial adaptation under various stress conditions.

## Results and discussion

### Profiling expression data of *M. tuberculosis* to identify sRNAs

We used RNA-Seq data of the mid-exponential phase culture to develop a methodology for identifying sRNAs in the intergenic regions (IGRs) of *M. tuberculosis* [21]. Initially, we quantified the expression of different functional elements in the genome and tested if the RNA-Seq data is sufficient to detect the IGR sRNA expression. Of the 4018 protein coding genes (CDS), 1000 highly expressing (HE) and less expressing (LE) genes respectively, were extracted (Additional file 9: Tables S3a and S3b). These were compared with the essential genes which show high expression levels [22–25] (Additional file 1: Figure S1). rRNAs are the abundantly expressed RNA species in the cell. Similarly, tRNAs show significant expression, which is comparable to the expression of the essential genes (Additional file 1: Figure S1). Further, we divided the IGR into untranslated regions (UTRs) and the absolute intergenic region (AbIGR) which is devoid of the UTRs. Both IGRs and AbIGRs show significant expression which is higher than the less expressed genes, implying functional importance of the non-protein coding regions in the genome. The high expression levels of the experimentally validated sRNAs encoded in the IGRs suggest that RNA-Seq could be potentially used to predict the location as well as the expression levels of sRNAs in the bacterial genomes (Additional file 8: Table S2; Additional file 1: Figure S1).

About 1037 IGRs of length more than 100 bp which are devoid of repeat regions, insertion elements, rRNAs and tRNAs were considered for identifying sRNAs (Methods). The distribution of these IGRs shows varied lengths ranging from 100 to 1500 bases (Additional file 2: Figure S2). As the given sRNA is unlikely to span the entire length of the IGR, a moving window approach was adopted to capture the expression of the sRNAs. The IGRs were covered by the windows of lengths 50 bases with 25 bases sliding and the expression of each of these windows was compared to its neighbouring windows to identify the peak expression signal. A window with an expression value of more than three times the median expression value of all the IGRs and showing higher expression compared to its adjacent windows was considered as a potential sRNA encoding region (Additional file 3: Figure S3; Methods).

Using this approach, we identified 119 IGR regions as significantly expressed in the mid-exponential growth phase which are likely to encode sRNA (Additional file 10: Table S4a). Of these, 52 and 2 expression regions were from the 5′ and the 3’UTRs of the neighbouring genes, respectively. These included three experimentally validated sRNAs, namely, ncRv13003Ac, ncRv3418Ac and ncRv13660Ac which are in the 5’UTR of their respective neighbouring genes. The rest 65 expression regions were localised in the AbIGR which included experimentally validated sRNAs ncRv11147Ac, ncRV2395, ncRv11534A and ncRv11846c [10, 11, 26].

### Increased transcription machinery binding in the expressed IGRs

For the 119 potential sRNA regions identified in the mid-exponential growth phase, we tested their expression by profiling the binding of RNA polymerase (RNAP) and NusA along these regions. RNA polymerase (RNAP) is the principal enzyme involved in synthesising of RNA from a DNA template. Another member of the transcription complex is NusA, a terminator and an anti-terminator of transcription which was shown to facilitate transcription by binding to RNAP in both mid-exponential and stationary phases of growth [27–29].

**Fig. 1:**
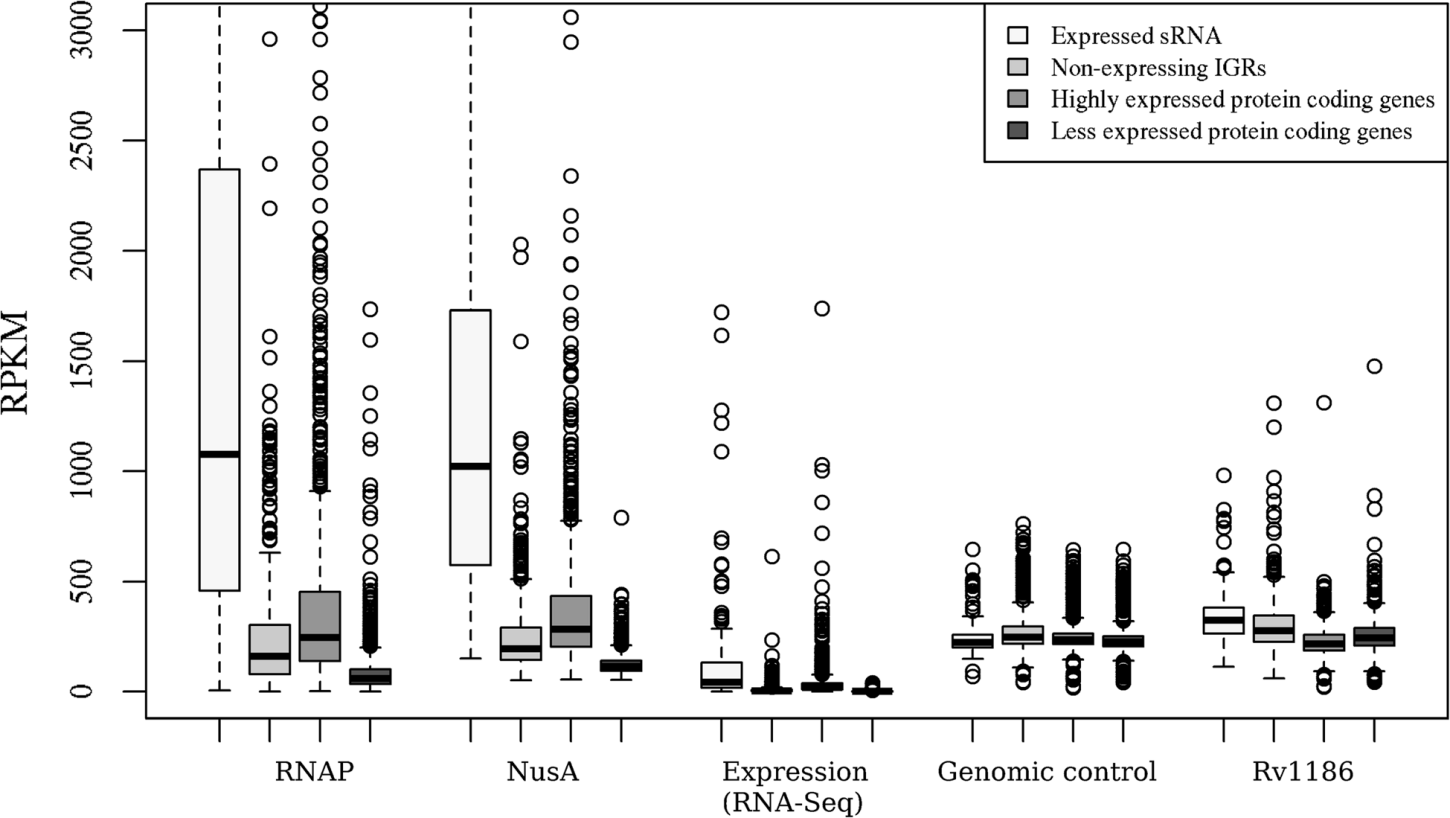
Binding of RNAP and NusA in the identified sRNA regions. Putative sRNA regions detected in the mid-exponential phase showed significant binding of RNAP and NusA compared to non-expressing IGRs (*P*-value < 0.05). They were also shown to be highly expressed in the RNA-Seq data associated with the ChIP-Seq experiments of RNAP and NusA (*P*-value < 4.424e-07). A similar binding profile was also seen for the highly expressed protein coding genes (*P*-value < 2.2e-16). However, this differential binding was not observed in the ChIP-seq data of the control sample Rv1186 and the genomic control

ChIP-seq data of RNAP and NusA in *M. tuberculosis* were used to test if these transcription-associated proteins are significantly bound to the identified sRNA regions on the genome [27]⁠. Additionally, ChIP-seq data of the polyketide synthase regulator Rv1186c and a genomic control sample attributing non-specific binding signals across the genome were used as control datasets (SRR1524124 and SRR5753731). We observe that the 119 expressed sRNA regions were significantly bound by both RNAP and NusA compared to the non-expressed IGRs (P value < 7.702e-10 and P value < 7.208e-06, respectively). Expression analysis using RNA-Seq data associated with RNAP and NusA experiments (SRP015746) showed that these putative sRNA regions are highly expressed compared to the non-expressed IGRs (P value < 4.424e-07). However, such a differential binding was not observed in the control samples Rv1186c and the genomic control (P value < 0.3239 and P value < 0.7858). Further, we performed a similar analysis for the highly expressed protein coding genes and the less expressed protein coding genes. As expected, the highly expressed protein coding genes showed increased binding of RNAP and NusA compared to the control samples (P-value < 2.2e-16) (Fig. 1). Therefore, sRNA regions identified in the mid-exponential growth phase seem to be significantly bound by the transcription machinery, suggesting transcriptional activity in these genomic regions.

### Context-dependent expression of sRNAs to revamp cellular responses

**Fig. 2:**
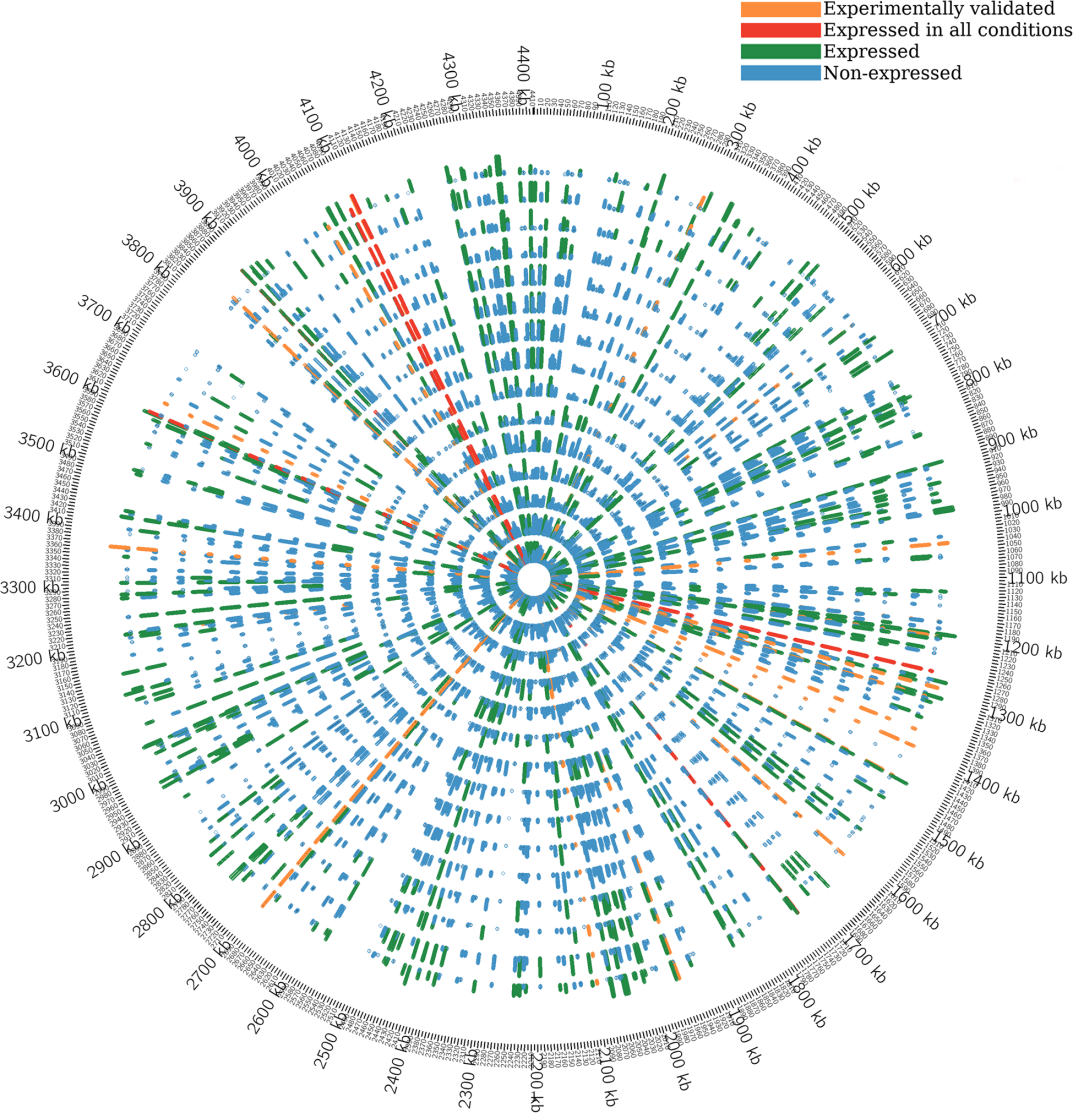
Genome-wide representation of the conditional expression of IGR sRNAs. The circles represent 15 growth conditions from innermost to the outer as ordered in Table S1. For each growth condition, the expression units range from 0 to 1500 RPKM. Experimentally validated sRNAs and sRNAs expressed in all conditions are highlighted in orange and red strokes respectively. Green strokes represent the expressed sRNAs and the blue strokes represent the absence of the sRNA expression

Similar to transcription factors, bacterial sRNAs show condition-specific expression which enables them to impart necessary cellular responses for a particular growth environment [7, 9, 12, 15]. The 119 sRNAs described earlier were identified in the mid-exponential phase growth culture. To gain more insights on the context-dependent expression of the sRNAs, we analysed *M. tuberculosis* RNA-Seq data of 5 different studies representing 15 different growth conditions. These included exponential and stationary growth phases, *M. tuberculosis* persistence and reactivation conditions, stress conditions such as iron depletion, NO treatment and acidic pH growth [27, 30, 31] (Additional file 8: Table S1). Of the 1037 IGRs considered in the analysis, we observed the expression of 430 putative sRNAs from 361 IGRs in at least one growth condition (Additional file 10: Table S4b; Additional file 4: Figures. S4a and S4b). Genome-wide representation of the IGR sRNA expression highlights the context-dependent expression of sRNAs along the *M. tuberculosis* genome (Figure 2).

**Fig. 3:**
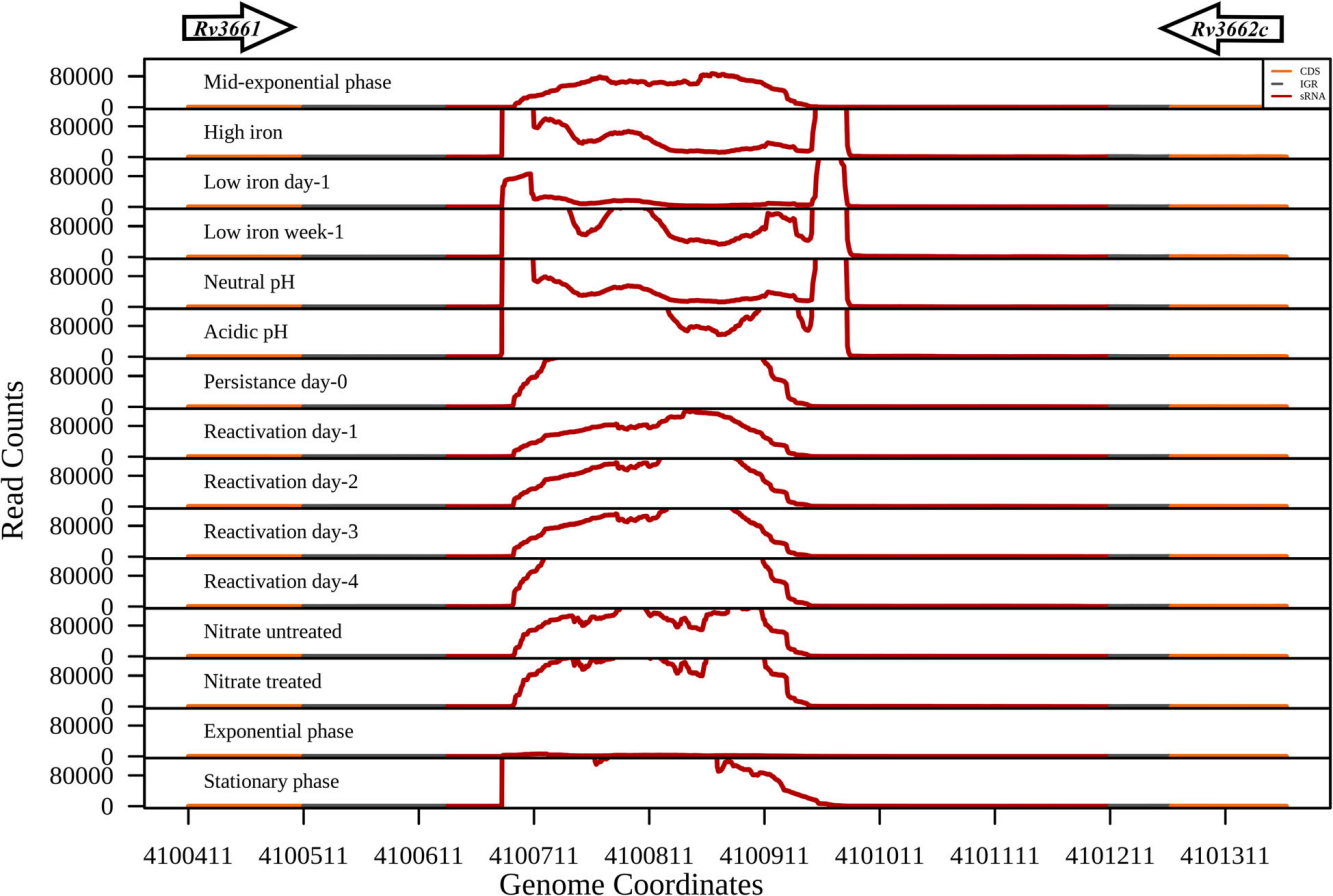
High expression of the MTS2823. Literature curated sRNA MTS2823, which is flanked by the genes *Rv3661* and *Rv3662c* (represented as arrows), shows expression in all the 15 growth conditions

**Fig. 4:**
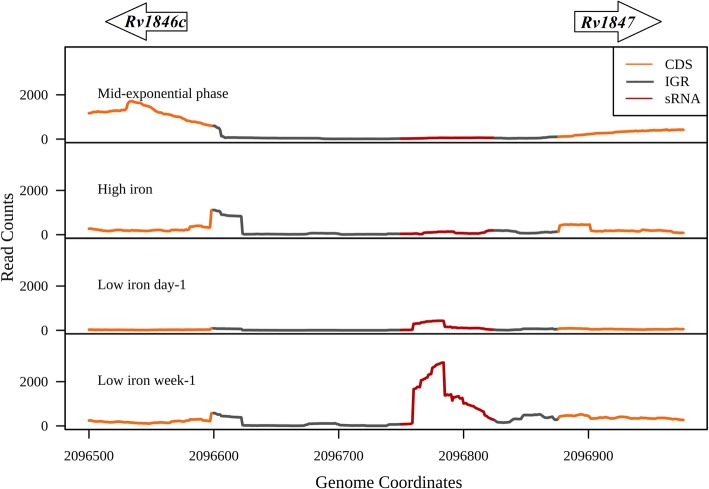
Expression of MrsI in iron rich and iron limiting conditions. sRNA MrsI expression is induced in low iron conditions, both at day-1 and week-1,compared to iron-rich growth conditions*.* The upstream and the downstream genes are represented as arrows

We captured the expression of 24 sRNAs from 42 experimentally validated intergenic sRNAs of *M. tuberculosis* (Table 1). Over-expression of the sRNA MTS2823 (ncRv13661) was previously shown to affect the growth rate of *M. tuberculosis* [11]*.* In our analysis, MTS2823 showed expression in all the 15 growth conditions with a very high expression in the stationary phase (Fig. 3). One of the promising targets predicted for MTS2823 is the gene Rv0115 (*hddA*) which codes for D-alpha-D-heptose-7-phosphate kinase, involved in GDP-L-fucose salvage pathway. Also, some of the other targets predicted for MTS2823 such as *hemD*, *Rv0875*, *ribH*, *mpt83*, *Rv3828c* and *Rv3839* were down-regulated by ≥2.5-fold upon over expression of MTS2823 [11]⁠. Another sRNA MrsI (ncRv11846) was shown to be induced during exposure to iron starvation, oxidative stress and membrane stress. MrsI represses the iron storage mRNA *bfrA* in iron deprived conditions [14]⁠. Along similar lines, we observe the induced expression of MrsI during iron limiting conditions. Also, MrsI expression is significantly induced in the late stages of iron deprivation compared to low iron day-1 (*P* value < 2.2e-16) (Fig. 4).

**Table 1:** Conditional expression of the 24 experimentally validated sRNAs. Of these, only 2 sRNAs were expressed in all the 15 growth conditions

sRNA	Start	End	Strand	Number of Expressed Conditions	PubMed Identifier
ncRv10243A (F6)	293604	293705	+	12	23284830
ncRv10537A	629877	629975	+	6	19555452; 22072964
ncRv10932Ac	1041165	1041129	–	4	20181675; 22072964
ncRv11051c (MTS0823)	1175225	1175315	+	2	22072964; 20181675
ncRv11075A	1200555	1200605	+	1	22072964
ncRv11147Ac (MTS0903)	1275549	1276297	–	8	20181675
ncRv11160A	1287126	1287201	+	13	23284830
ncRv11174Ac	1306073	1306038	–	3	20181675
ncRv1222A	1365274	1365365	+	5	22452820
ncRv11248c	1393055	1393140	+	2	20181675; 22072964
ncRv11296A	1453007	1453060	+	13	20181675
ncRv11435c	1612987	1613047	+	1	23284830
ncRv11534A	1735693	1735747	+	15	19555452; 22072964
ncRv1734A (MTS1338)	1960667	1960783	+	7	24244498
ncRv11846Ac (MrsI)	2096839	2096768	+	4	29871950
ncRv2395A	2692172	2692521	+	5	20181675
ncRv12560A	2881252	2881320	+	2	20181675
ncRv12904A	3214341	3214399	+	9	23284830
ncRv13003Ac	3363153	3363023	–	2	20181675
ncRv13241Ac	3621466	3621265	–	11	23284830
ncRv3418Ac	3837458	3837288	–	13	23284830
ncRv13596A (MTS2774)	4040879	4040938	+	5	20181675
ncRv13660Ac (MTS2822)	4099478	4099386	–	13	20181675; 22072964
ncRv13661A (MTS2823)	4100669	4100968	+	15	20181675

**Fig. 5:**
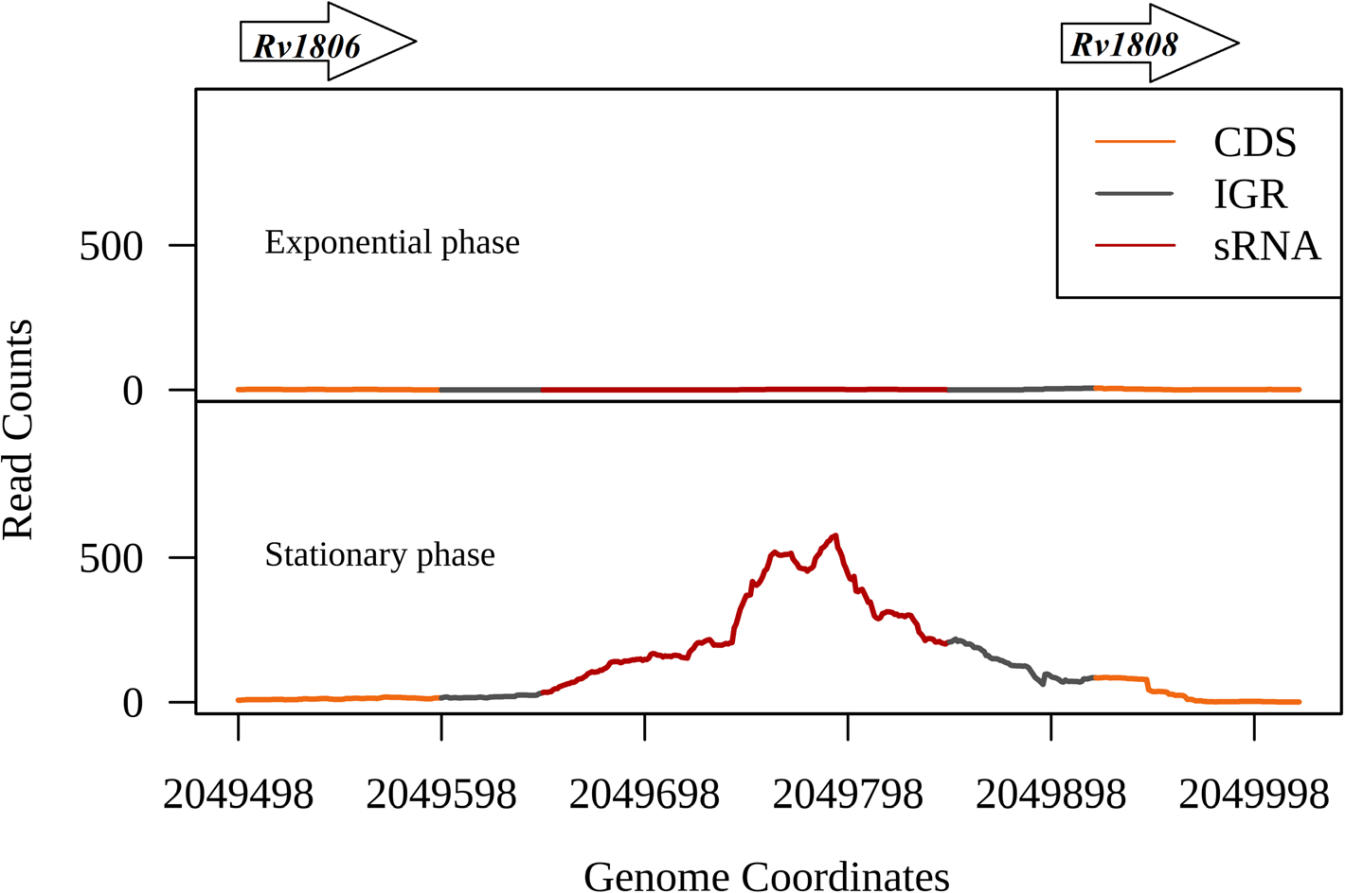
Expression of ncRv11806 in the exponential and stationary phases of growth. sRNA ncRv11806 expression is induced in the stationary phase compared to the exponential growth phase. ncRv11806 lies in the IGR between the genes *PE20* and *PPE32* (represented as arrows)

While 6 sRNAs are expressed irrespective of the growth condition, the rest of the sRNAs showed context-dependent expression (Fig. 2 and Additional file 4: Figure. S4b). The sRNA ncRv11806, which is flanked by the genes PE20 and PPE32*,* showed expression only in the stationary phase of growth (*P* value < 2.2e-16) (Fig. 5). Some of the potential targets predicted for this region were *rpfB*, *nrdH*, *memE*, *thyX*, *senX3* and *mutT1* (Additional file 11: Table S5a). The expression of *rpfB* which codes for a resuscitation promoting factor (RpfB) gets diminished as the pathogen transits into stationary phase [32] (Additional file 5: Figure S5A). ncRv11806 is predicted to bind at the 5’UTR region of *rpfB* mRNA, which extends further to the protein coding region (Additional file S5: Figure S5B). We therefore hypothesise that the induced expression of ncRv11806 in the stationary phase of *M. tuberculosis* growth might repress the translation of *rpfB*.

**Fig. 6:**
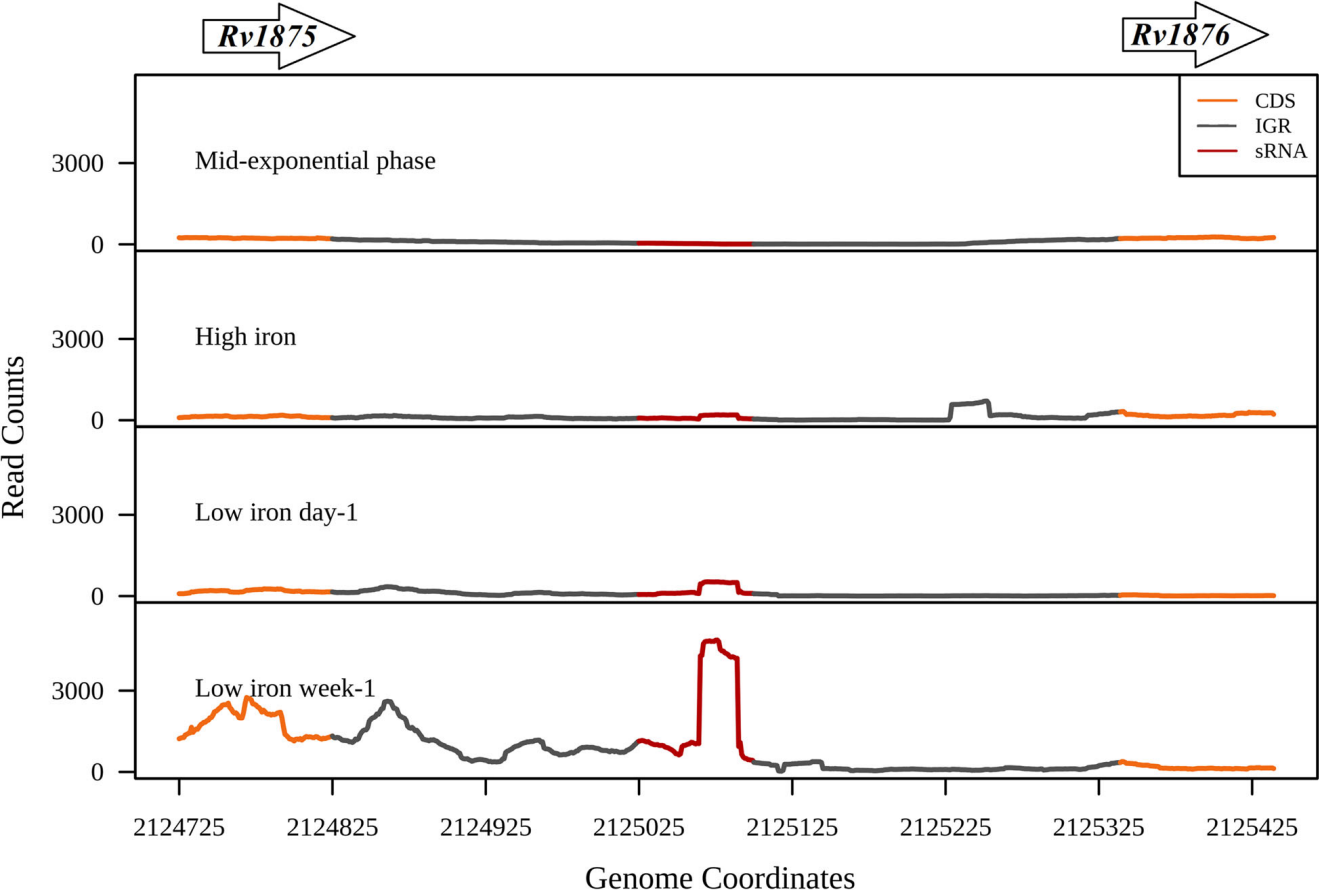
Expression of ncRv11875C in iron rich and iron limiting conditions. sRNA ncRv11875C which is flanked by the genes *Rv1875* and *bfrA* is induced in the iron limiting conditions (day-1 and week-1) compared to the iron rich environment. The upstream and the downstream genes are represented as arrows

Another sRNA ncRv11875C located between the genes *Rv1875* and *bfrA* showed significant expression in the iron limiting conditions (P value < 2.2e-16) (Fig. 6). Genes *Rv3003c, Rv1924c, Rv3150, Rv1937, Rv1728c, Rv1626, Rv1308, Rv0544c, Rv0532* and *Rv1526c* are the predicted targets for this sRNA (Additional file 11: Table S5b). Subsequent gene expression analysis revealed that the predicted targets *Rv1308, Rv3003c, Rv1924c, Rv1728c, Rv1626* and *Rv3150* showed reduced expression in the iron limited condition compared to mid-exponential and high iron growth conditions (Additional file 6: Figure S6). On the other hand, *Rv1937,* which is a probable monooxygenase containing [2Fe-2S] cluster shows increased expression in the iron limited conditions, suggesting a probable positive regulation by ncRv11875C (Additional file 6: Figure S6).

**Fig. 7:**
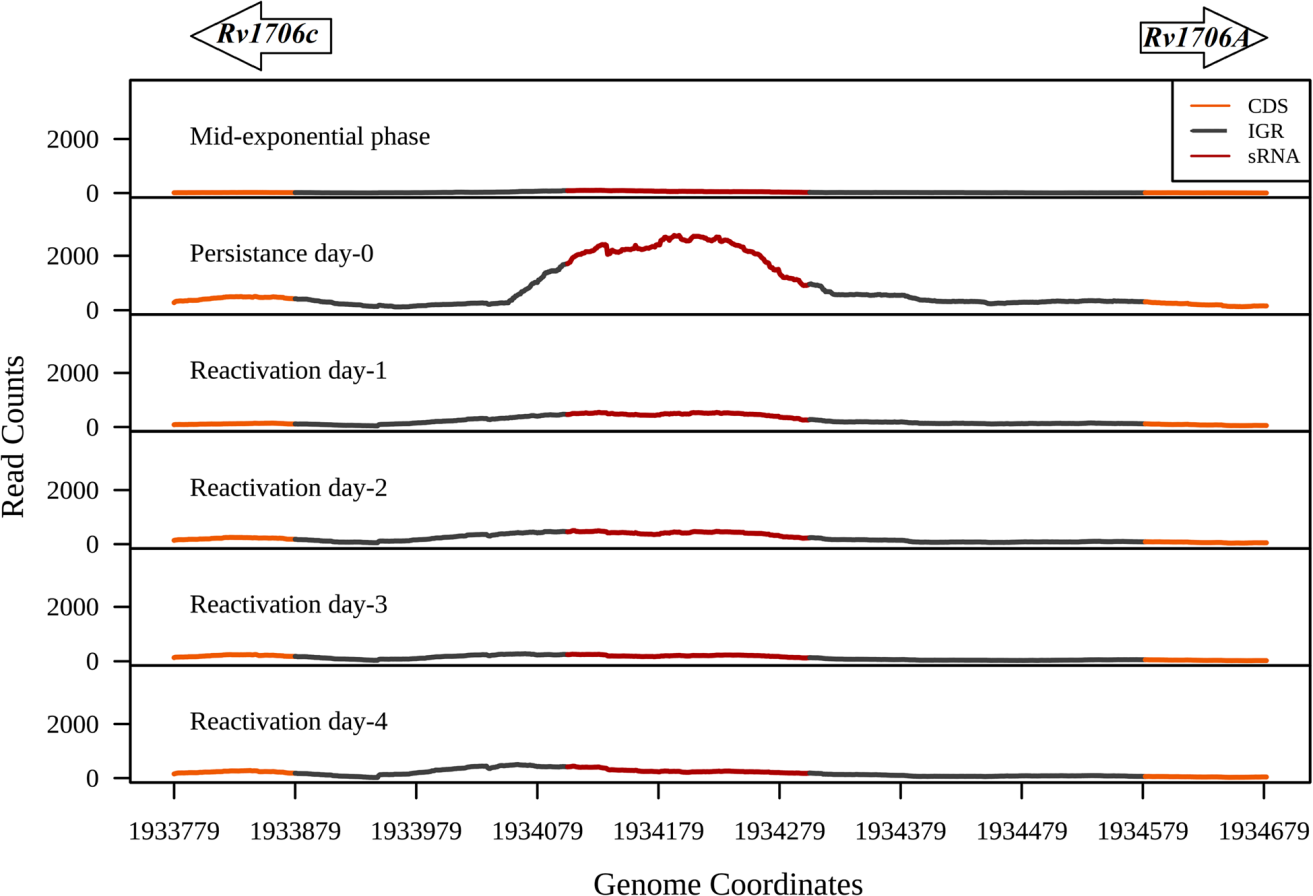
Expression of persistence and reactivation specific sRNAs. sRNA ncRv11706A which is flanked by the genes *Rv1706c* and *Rv1706A* (represented as arrows) showed expression in persistence compared to the exponential phase and all the stages of reactivation

*M. tuberculosis* persists in the host with a reduced metabolic activity and gets reactivated upon encountering favourable conditions for growth [2]. However, the regulatory roles of mycobacterial sRNAs in these growth phases remain poorly understood. We observe that the sRNA ncRv11706A, which is in the intergenic region between *Rv1706A* and *Rv1706c*, is highly expressed in hypoxia induced persistence (*P* value < 2.2e-16) (Fig. 7). Some of the predicted targets shortlisted for this sRNA such as *Rv3158, Rv2736c, Rv1382, Rv2325c* and *Rv2898c* showed reduced expression in persistence compared to mid-exponential growth and various reactivation phases (Additional file 11: Table S5c; Additional file 7: Figure S7A). On the other hand, predicted target genes *Rv3047c* and *Rv3102c* showed increased expression in persistence compared to mid-exponential and reactivation conditions (Additional file 7: Figure S7A). Among these, *Rv2736c* encoding RecX was repressed in persistence significantly. RecX, which modulates the activity of RecA by inhibiting its ATP hydrolysis and the strand-exchange activities, was shown to be significantly downregulated in SS18b model which mimics latent TB infection [33, 34]*.* ncRv11706A is predicted to interact at 3 bases downstream of the start codon of *recX* mRNA, suggesting that this interaction might affect the translation, thereby repressing RecX activity (Additional file 7: Figure S7B). These conditionally expressed sRNAs in the context of their predicted targets and the functions, therefore, provide insights on bacterial adaptability to changing growth environments.

## Conclusions

Bacterial genomes encode both *cis* and *trans*-acting sRNAs which are important for the regulation of cellular functions [4]. Since sequence conservation is poor for the sRNAs across species, homology-based methods are less powerful in identifying sRNAs [35]. Previously, there were attempts to determine sRNA coding regions in the bacterial genomes using expression data [18, 19]. However, the challenges while using such an approach include discriminating between sRNA expression signal and the noise arising from IGRs, and to systematically eliminate the signals which are associated with the neighbouring gene expression. We have devised a novel, moving-window based method for detecting sRNA expression in the IGRs. Our method is elegant in capturing both validated and novel sRNA expression, with reduced influence of the expression signals arising from both the upstream and the downstream gene UTRs. As RNA-Seq is used as the input, the same data allows for the simultaneous quantification of both protein-coding gene as well as sRNA expression. Using this method, we identified 119 IGR sRNAs in the mid-exponential growth phase, which also exhibit preferential binding for the transcription machinery.

Mycobacteria encounter diverse environments in the host such as nutrient depletion, hypoxia and iron limitation. Profiling of sRNAs in multiple conditions is therefore essential to understand the expression dynamics of sRNAs, which correlates with the conditional responses of the cell. Our extended analysis of the repertoire of RNA-Seq data to detect sRNA expression revealed context-dependent expression of many sRNAs. As case studies, we chose some of these novel sRNAs identified by our method and attempted to explain their potential regulatory role by predicting gene targets. One such sRNA is ncRv11806 which shows expression in the stationary phase of growth. Resuscitation promoting factor (RpfB) is one of its predicted gene targets which is required for the revival of dormant bacteria. It is interesting to note that the binding of ncRv11806 to the *rpfB* mRNA masks the 5′ UTR and the start codon, which likely hinders translation. Also, ncRv11706A appears as the hypoxia-induced persistence specific sRNA. One of the targets for ncRv11706A is *recX,* the expression of which is downregulated in the latent mycobacterial infection. By inspecting the putative binding sites, it appears that the sRNA ncRv11706A masks the start codon on the *recX* mRNA. Therefore, our methodology for identifying sRNAs and subsequent cataloguing of their context-dependent expression generated novel perspectives on the sRNA mediated regulation. Further experimental validation of these putative sRNAs and their regulatory mechanisms will provide more insights on *M. tuberculosis* host adaptation and pathogenesis.

## Methods

### Downloading and processing expression data

RNA-Seq data of *M. tuberculosis* H37Rv were retrieved from the NCBI-Sequence Read Archive (NCBI-SRA) (https://www.ncbi.nlm.nih.gov/sra) and European Nucleotide Archive (ENA) (https://www.ebi.ac.uk/ena) databases. Based on the library size quality, 5 different studies with 15 unique growth conditions were chosen for the analysis (Additional file 8: Table S1). SRA files were converted to fastq using fastq-dump available in sra-toolkit.2.1.18. The adapter sequences and the reads with the mean Phred score < 15 were removed using trimmomatic (v 0.36) [36]⁠. Trimmed data were aligned to the *M. tuberculosis* H37Rv reference genome (NC_000962.3) [37] using bowtie2 (v 2.1.0) [38] and the resulting SAM files were converted into BAM files. BAM files for the replicate datasets were merged, sorted and indexed using samtools (v 0.1.18) [39].

### Selecting IGRs for the analysis

Intergenic region (IGR) is defined as the genomic region between two protein-coding regions, and absolute intergenic region (AbIGR) is the IGR devoid of the UTRs. The genome size of *M. tuberculosis* H37Rv is 4411532 bp, which consists of 4018 proteins coding regions, 3 rRNAs and 45 tRNAs [37]. Of the 4017 Intergenic regions (IGRs), following were excluded from the analysis: i) 830 IGRs between operonic genes [40] ii) 48 IGRs encoding tRNAs and rRNAs [37] iii) 41 IGRs which contain mycobacterium specific repeat regions such as MIRU, VNTR [41] iv) 46 IGRs with Insertion elements such as IS6110 (16 copies), IS1018 (6 copies), REP13E12 (14 copies) and other IS (11 copies) [37] and v) IGRs which have a length smaller than 100 bp. This resulted in a total of 1037 IGRs for further analysis.

Genome coordinates of the protein-coding regions, tRNAs, rRNAs and repeat regions were retrieved from the NCBI Refseq gene annotation file (https://www.ncbi.nlm.nih.gov/refseq/) [37]. Transcription termination (3’UTR) coordinates were derived from the WebGeSTer database [42]. Coordinates of the transcription start site were obtained from the study by Cortes et al. [43]⁠. From the intergenic regions, both the 3′ and the 5′ UTRs were removed to get the absolute intergenic region (AbIGR). MultiBamcov in Bedtools was used to obtain read counts which were further normalised using RPKM (Reads Per Kilobase Million) [44]⁠. In the mid-exponential phase growth culture, top 1000 genes with high expression, which correspond to more than third quartile (Q3) of the dataset were identified as highly expressed (HE) genes [21]. Similarly, bottom 1000 genes with the least expression, which correspond to less than first quartile (Q1) of the dataset were identified as less expressed (LE) genes [21]. For comparison, experimentally validated sRNAs in *M. tuberculosis* were curated from the available literature (Additional file 8: Table S2).

### Identifying IGR expression

To identify significant expression in the IGRs, windows of 50 base length with 25 bases sliding were used. In each IGR, a window was termed expressed if a) its expression value (RPKM) is greater than or equal to three times the median expression of the IGRs which are devoid of operons and repeat regions, and b) is greater than the adjacent windows. The first and the last windows in each IGR were masked to avoid the misclassification of the UTR expression as sRNA expression. The windows which overlap with the annotated pseudogenes were excluded. The expressing windows were merged to obtain putative sRNAs if they appear as contiguous in terms of genomic coordinates.

### ChIP-Seq data analysis

Differential binding of RNAP and NusA in the expressed IGRs in mid-exponential phase was analysed using ChIP-Seq datasets GSM1003214 and GSM1003222, respectively [27]. These were compared with the ChIP-Seq data of the transcription factor Rv1186 (SRR1524124) and a genomic control (GSM2683113). Fastq-dump function of the sra-toolkit-2.1.18 was used to retrieve fastq format of the data. Trimmomatic (v 0.36) was used to trim the adapter sequences and the reads with the mean Phred score < 15 [36]. The data were aligned to the reference genome using bowtie2 (v 2.1.0) [38]. Aligned SAM files were converted to BAM files which were further sorted and indexed by samtools. From the sorted bam files, readcounts were obtained using multiBamCov of bedtools [44], which were further normalised to RPKM values to represent the binding profile. Welch Two Sample t-test was performed using R (https://www.r-project.org/) to compare the binding of transcription machinery to the expressed sRNAs encoding regions and the non-expressed IGRs.

### Gene targets of the identified sRNAs

Genome-wide putative termination sites were derived using iTerm-PseKNC which were further used to determine the strands of the expressed sRNAs [45]. Potential gene targets of the sRNAs were derived using target prediction tools IntaRNA and TargetRNA2 [46, 47]. The reference genome of *M. tuberculosis* (NC_000962.3) was used as a target input file. Of the list of predicted targets with *p*-value < 0.05, we considered 10 genes with minimum binding energy and the least p-value as potential targets for further analysis. Expression of the target genes in each of the 15 RNA-Seq data was quantified using bedtools [44]. The read counts were normalised by calculating RPKM.

All statistical analyses were performed using R (https://www.r-project.org/). The statistical significance of the conditional expression of sRNAs was assessed using Wilcoxon signed rank test. Data were analysed using in-house shell and python scripts. Circos version-0.69 was used to represent the IGR expression along the circular chromosome co-ordinates [48]⁠.

## Supplementary information

**Additional file 1 Figure S1.** Expression profile of different functional categories in the mid-exponential growth phase. sRNAs are expressed on par with highly expressed and known essential genes. IGRs and AbIGRs, which are the potential sRNA encoding regions, also show significant expression suggesting functional relevance of the non-protein coding regions. (CDS - Protein coding regions, EG - Essential genes, HE – Highly expressed genes, LE - Less expressed genes, IGR - Intergenic regions, AbIGR - Absolute intergenic region devoid of neighbouring gene UTRs, and sRNA – curated sRNAs).

**Additional file 2 Figure S2.** Length distribution of 1037 IGRs chosen for the study. The IGR length ranges from 100 bp to 1500 bp.

**Additional file 3 Figure S3.** Moving-window approach to identify sRNA expression. The IGR between the two protein-coding regions (CDS) including untranslated regions (5’UTR and 3’UTR) was slided with windows of length 50 base with 25 bases sliding. In this cartoonic representation, the genomic region covered by the 3rd window is predicted to encode an sRNA as it shows expression higher than the cut-off (the solid line) and the expression greater than the adjacent windows.

**Additional file 4 Figure S4.** Expression of IGRs across growth conditions. (A) Number of sRNAs expressed in each of the growth conditions studied (B) Frequency distribution of the number of sRNAs versus the number of the expressed growth conditions. Of the 430 sRNAs, 6 showed expression in all the 15 growth conditions and 48 sRNAs were expressed in more than 10 growth conditions.

**Additional file 5 Figure S5.** Expression of the predicted target genes of ncRv11806.(A) Expression of the predicted gene targets of the sRNA ncRv11806 quantified in both exponential and stationary phases of growth. (B) Base pairing of ncRv11806 and one of its targets *Rv1009*. The 5’UTR region of *rpfB* is underlined and the start codon AUG is highlighted in bold letters.

**Additional file 6 Figure S6.** Expression of the target genes of ncRv11875C. Expression of some of the predicted gene targets of the sRNA ncRv11875C which are differentially expressed in low iron conditions compared to mid-exponential and high iron growth.

**Additional file 7 Figure S7.** Expression of the target genes of ncRv11706A. (A) Expression of few of the target genes of ncRv11706A which are differentially expressed at persistence day-0 compared to mid-exponential growth phase and various time points of reactivation. (B) Base pairing of ncRv11706A and one of its targets *recX*. The start codon is highlighted in bold letters.

**Additional file 8 Table S1.** List of the RNA-Seq data. Description of the 15 RNA-Seq datasets used in the study along with their SRA study and accession numbers, growth conditions, and the data type. **Table S2.** Experimentally validated sRNAs. List of literature curated *M. tuberculosis* sRNAs which are experimentally validated.

**Additional file 9 Table S3.** Highly expressed and less expressed genes in the mid-exponential growth phase. List of highly expressed (a) and less expressed genes (b) protein coding genes in the mid-exponential growth phase with their RPKM values and the gene coordinates.

**Additional file 10 Table S4.** Context-dependent expression of the sRNAs. (a) List of 119 sRNAs expressed in the mid-exponential growth phase. (b) Context-dependent expression of all the 430 sRNAs derived by our method. (c) Binary matrix representation of the context-dependent expression of sRNAs across 15 growth conditions. (d) Expression matrix of the identified sRNAs in all the 15 growth conditions.

**Additional file 11 Table S5.** Target genes of some of the sRNAs. Predicted gene targets of the sRNAs along with their quantified expression in relevant growth conditions. Targets of (a) ncRv11806, (b) ncRv11875 and (c) ncRv11706A.

## Data Availability

All the information relevant to the data analysed in this study are available in the Additional file 8.

## Abbreviations

AbIGR: Absolute Intergenic Region
CDS: Protein coding genes
ChIP-Seq: Chromatin Immunoprecipitation sequencing
IGR: Intergenic regions
RNAP: RNA Polymerase
RPKM: Reads Per Kilobase Million
sRNA: Small RNAs
UTR: Untranslated Regions
