# Supplementary figures and images for: Genome-wide identification of the context-dependent sRNA expression in Mycobacterium tuberculosis
Source: BMC Genomics. 2020 Feb 18;21:167. doi: 10.1186/s12864-020-6573-5 (PMC7029489; doi:10.1186/s12864-020-6573-5)

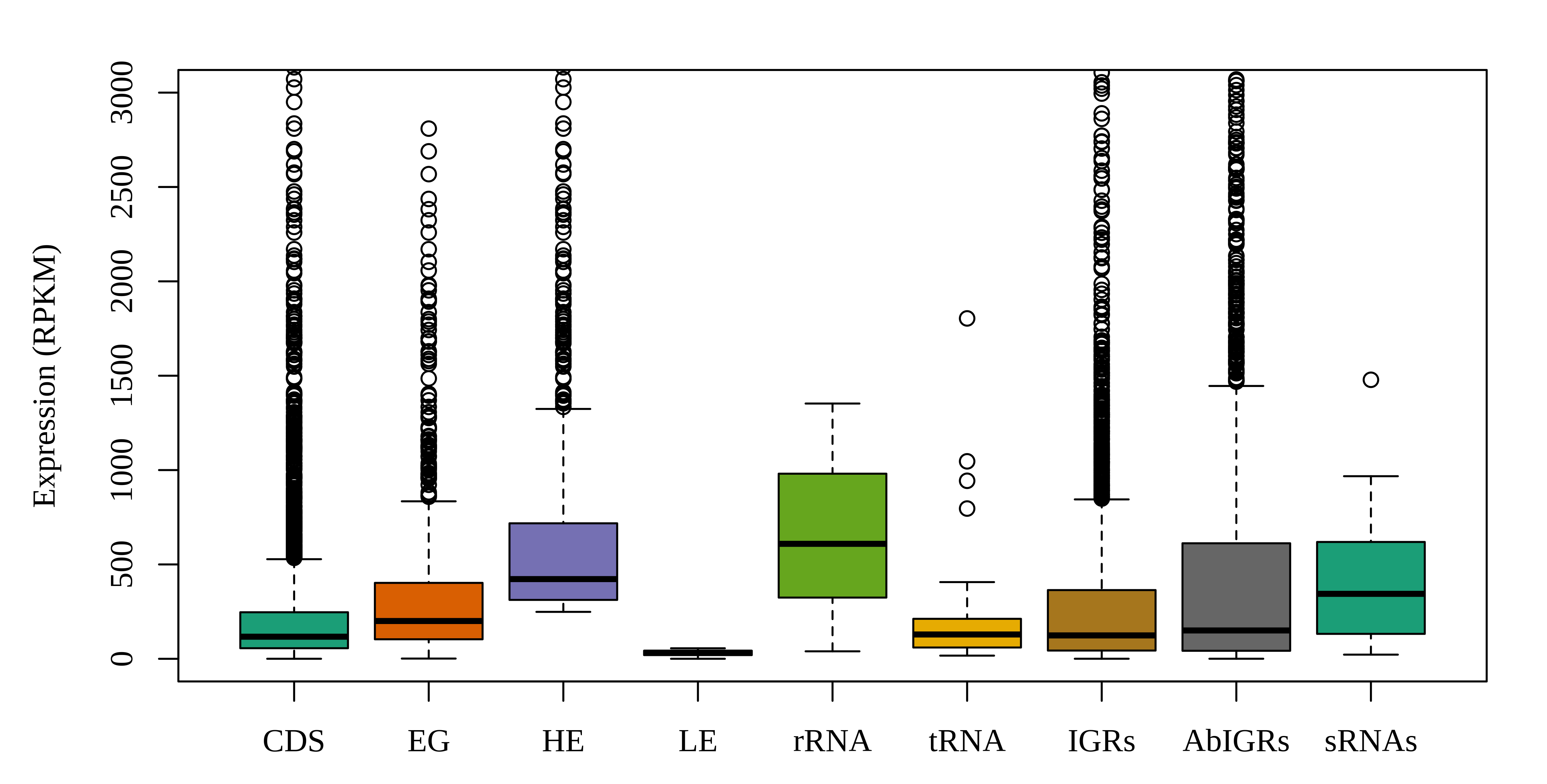

Supplement: Supplementary file 1 — Additional file 1 Figure S1. Expression profile of different functional categories in the mid-exponential growth phase. sRNAs are expressed on par with highly expressed and known essential genes. IGRs and AbIGRs, which are the potential sRNA encoding regions, also show significant expression suggesting functional relevance of the non-protein coding regions. (CDS - Protein coding regions, EG - Essential genes, HE – Highly expressed genes, LE - Less expressed genes, IGR - Intergenic regions, AbIGR - Absolute intergenic region devoid of neighbouring gene UTRs, and sRNA – curated sRNAs). [file 12864_2020_6573_MOESM1_ESM.tiff]

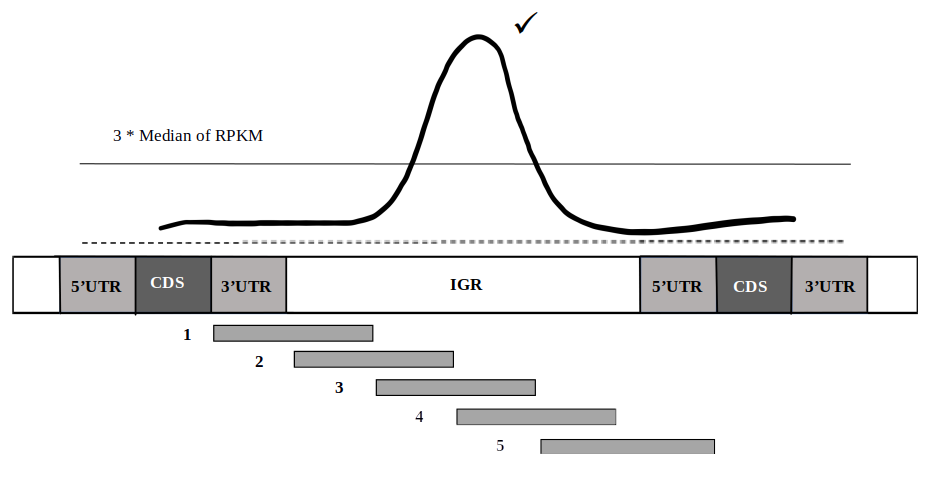

Supplement: Supplementary file 3 — Additional file 3 Figure S3. Moving-window approach to identify sRNA expression. The IGR between the two protein-coding regions (CDS) including untranslated regions (5’UTR and 3’UTR) was slided with windows of length 50 base with 25 bases sliding. In this cartoonic representation, the genomic region covered by the 3rd window is predicted to encode an sRNA as it shows expression higher than the cut-off (the solid line) and the expression greater than the adjacent windows. [file 12864_2020_6573_MOESM3_ESM.tiff]

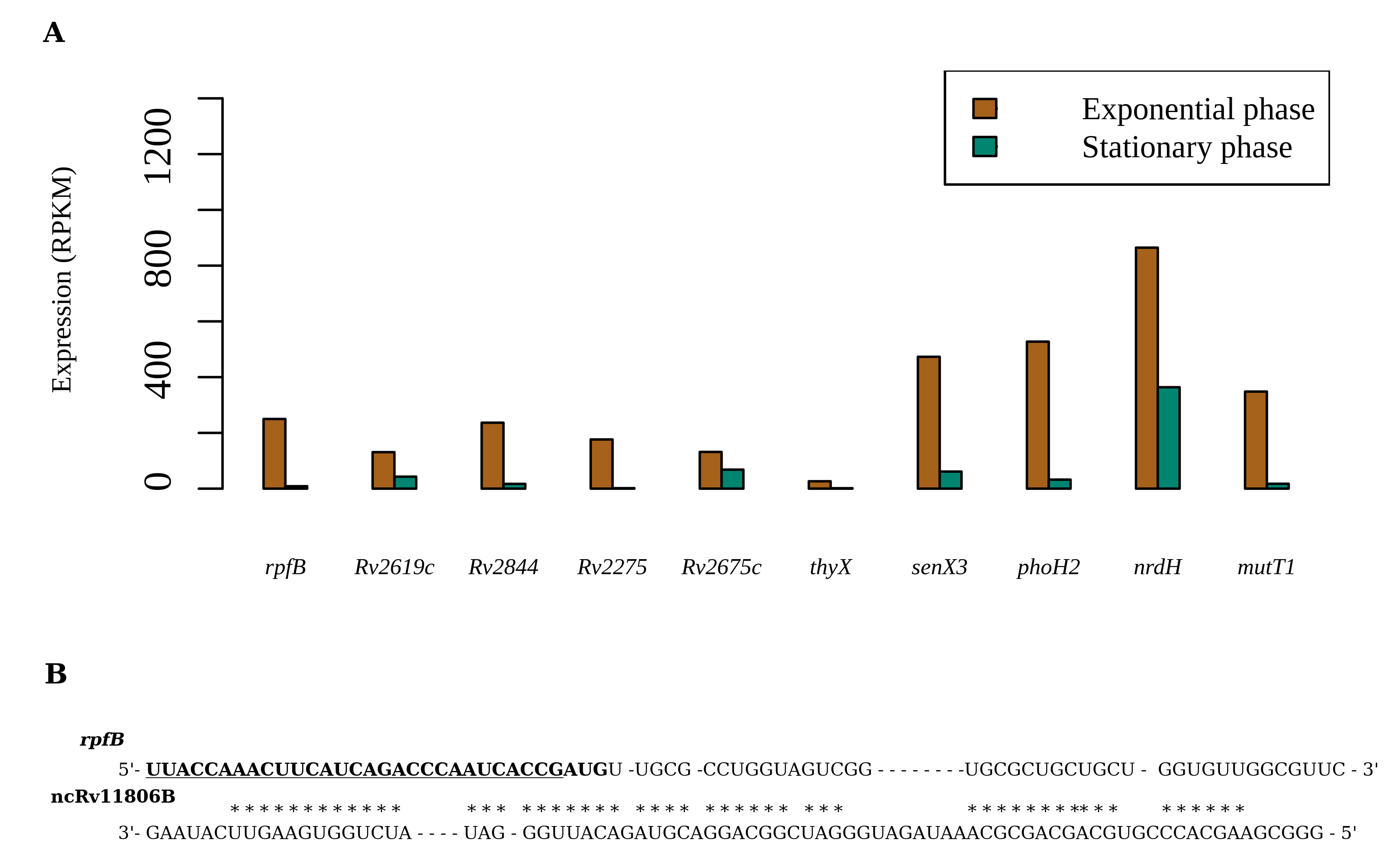

Supplement: Supplementary file 5 — Additional file 5 Figure S5. Expression of the predicted target genes of ncRv11806.(A) Expression of the predicted gene targets of the sRNA ncRv11806 quantified in both exponential and stationary phases of growth. (B) Base pairing of ncRv11806 and one of its targets Rv1009. The 5’UTR region of rpfB is underlined and the start codon AUG is highlighted in bold letters. [file 12864_2020_6573_MOESM5_ESM.tiff]

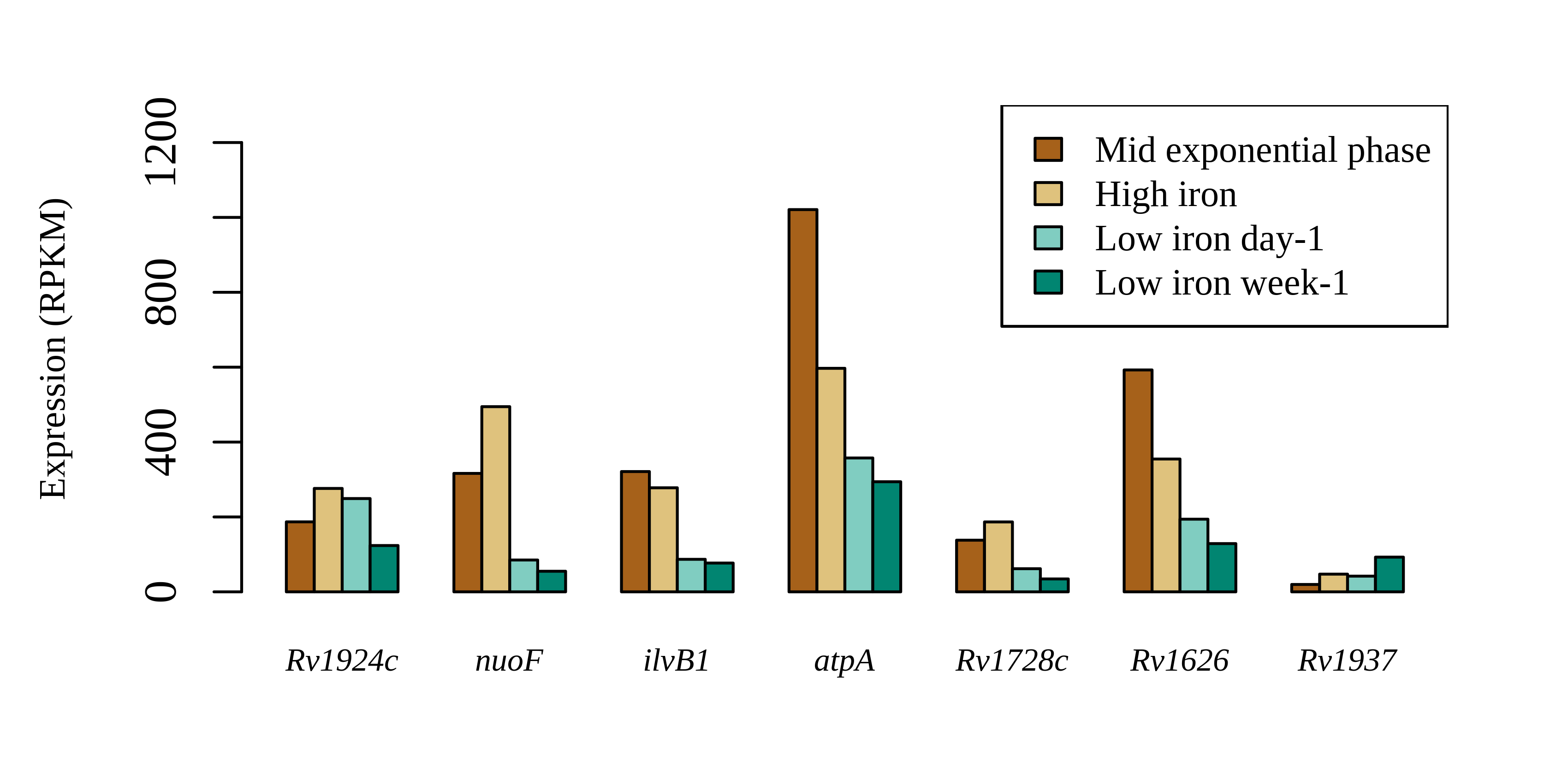

Supplement: Supplementary file 6 — Additional file 6 Figure S6. Expression of the target genes of ncRv11875C. Expression of some of the predicted gene targets of the sRNA ncRv11875C which are differentially expressed in low iron conditions compared to mid-exponential and high iron growth. [file 12864_2020_6573_MOESM6_ESM.tiff]

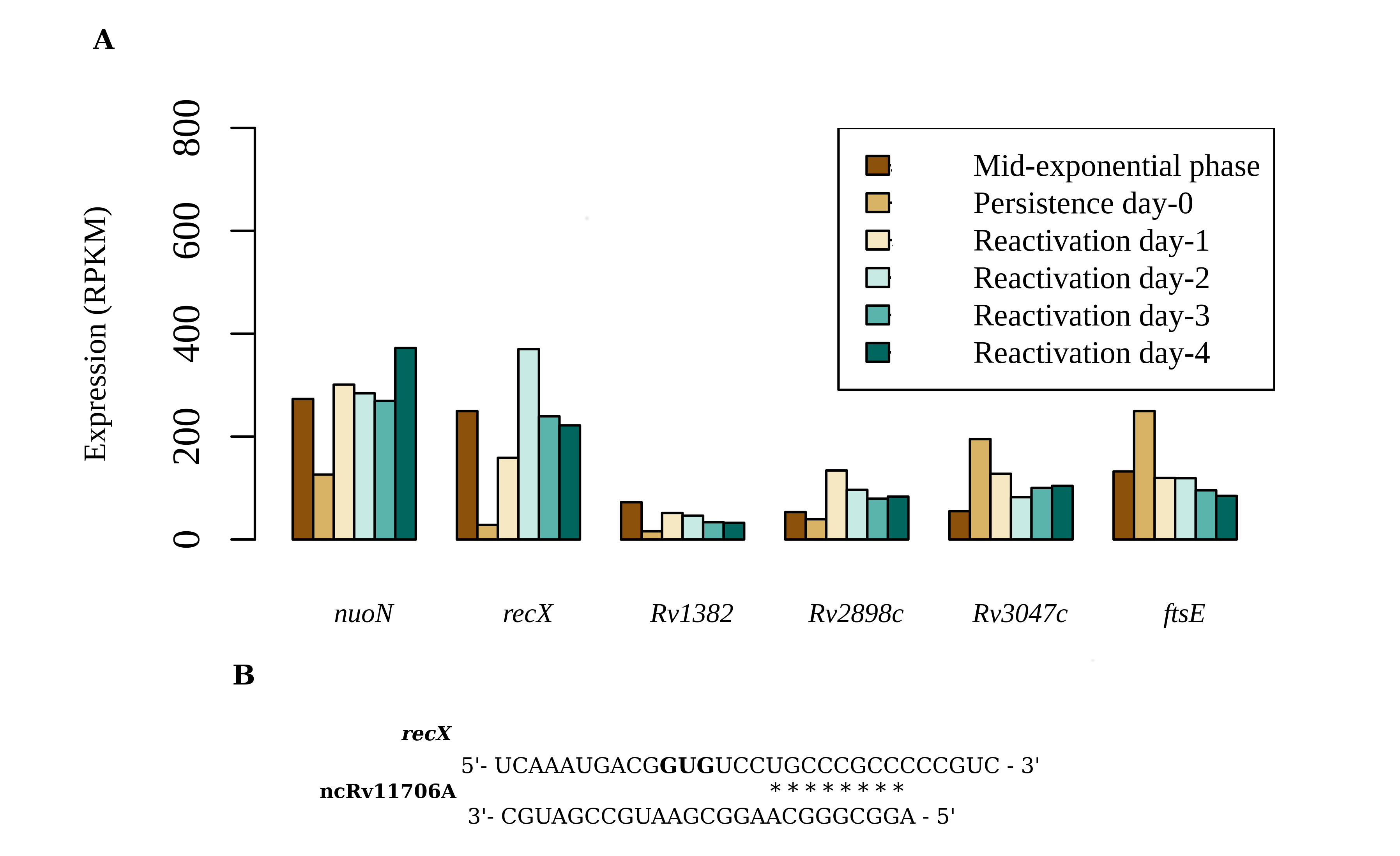

Supplement: Supplementary file 7 — Additional file 7 Figure S7. Expression of the target genes of ncRv11706A. (A) Expression of few of the target genes of ncRv11706A which are differentially expressed at persistence day-0 compared to mid-exponential growth phase and various time points of reactivation. (B) Base pairing of ncRv11706A and one of its targets recX. The start codon is highlighted in bold letters. [file 12864_2020_6573_MOESM7_ESM.tiff]
