# Supplementary material for: Genome-wide identification of the context-dependent sRNA expression in Mycobacterium tuberculosis
Source: BMC Genomics. 2020 Feb 18;21:167. doi: 10.1186/s12864-020-6573-5 (PMC7029489; doi:10.1186/s12864-020-6573-5)
Supplement: Supplementary file 8 — Additional file 8 Table S1. List of the RNA-Seq data. Description of the 15 RNA-Seq datasets used in the study along with their SRA study and accession numbers, growth conditions, and the data type. Table S2. Experimentally validated sRNAs. List of literature curated M. tuberculosis sRNAs which are experimentally validated. [file 12864_2020_6573_MOESM8_ESM.pdf]

Table S1. List of the RNA-Seq data. Description of the 15 RNA-Seq datasets used in the study along with their SRA study and accession numbers, growth conditions, and the data type.

SRA study	SRA Accession	Condition	Data type	Total reads mapped to the IGR (in million)
SRP184670	SRR8550302<br>SRR8550303<br>SRR8550304	Mid-Exponential phase	Paired	1.54<br>1.47<br>1.28
SRP056290	SRR1917703<br>SRR1917704<br>SRR1917705	High iron	Paired	5.04<br>3.46<br>1.63
SRP056290	SRR1917706<br>SRR1917707<br>SRR1917708	Low iron 1day	Paired	0.62<br>3.74<br>5.98
SRP056290	SRR1917709<br>SRR1917710<br>SRR1917711	Low iron 1week	Paired	4.68<br>2.23<br>3.54
SRP056290	SRR1917712<br>SRR1917713	Tyloxapol pH7.0	Paired	3.84<br>6.72
SRP056290	SRR1917714<br>SRR1917715	Tyloxapol pH5.5	Paired	6.81<br>8.56
SRP077489	SRR3725585<br>SRR3725586<br>SRR3725587	Persistance day 0 (hypoxia)	Paired	2.62<br>2.71<br>2.4
SRP077489	SRR3725588<br>SRR3725589<br>SRR3725590	Reactivation day1	Paired	2.26<br>2.09<br>2.34
SRP077489	SRR3725591<br>SRR3725592<br>SRR3725593	Reactivation day2	Paired	2.5<br>1.88<br>2.3
SRP077489	SRR3725594<br>SRR3725595<br>SRR3725596	Reactivation day3	Paired	1.73<br>2.79<br>2.71
SRP077489	SRR3725597<br>SRR3725598<br>SRR3725599	Reactivation day4	Paired	2.39<br>2.52<br>3.08
SRP015746	SRR568038<br>SRR568039	Exponential phase	Single	0.21<br>1.38
SRP015746	SRR568040	Stationary phase	Single	1.78
SRP030118	SRR998928<br>SRR998929<br>SRR998930	Nitrate Untreated MTB	Single	0.56<br>1.01<br>0.63

SRP030118	SRR998931<br>SRR998932<br>SRR998933	Nitrate treated MTB	Single	0.99<br>1.4<br>1.34

Table S2. Experimentally validated sRNAs. List of literature curated *M. tuberculosis* sRNAs which are experimentally validated.

sRNA	Start	End	Strand	Location	Pubmed Identifier
ncRv10150Ac	177282	177441	-	Intergenic	19555452;<br>20181675;<br>22072964;
ncRv10243A	293604	293705	+	Intergenic	23284830
ncRv10537A	629877	629975	+	5'/3' UTRs	19555452;<br>22072964
ncRv10609AA	704187	704247	+	Intergenic	23284830
ncRv10710A	806185	806218	+	5'/3' UTRs	19555452
ncRv10932Ac	1041165	1041129	-	Intergenic	20181675;<br>22072964
ncRv11051c	1175225	1175315	+	Intergenic	22072964;20181675
ncRv11075A	1200555	1200605	+	Intergenic	22072964
ncRv11092c	1220388	1220487	-	Intergenic	22072964;<br>23284830
ncRv11147Ac	1275549	1276297	-	Intergenic	20181675;
ncRv11160A	1287126	1287201	+	Intergenic	23284830
ncRv11174Ac	1306073	1306038	-	5' UTR	20181675
ncRv1222A	1365274	1365365	+	Intergenic	20181675
ncRv11230c	1374224	1374270	+	Intergenic	22452820
ncRv11248c	1393055	1393140	+	Intergenic	20181675;<br>22072964
ncRv11264Ac	1413224	1413094	-	Intergenic	23284830
ncRv11296A	1453007	1453060	+	3' UTR	20181675
ncRv11315A	1471619	1471742	+	Intergenic	23284830
ncRv1nr02A	1476825	1476884	+	3' UTR or 5' UTR	20181675
ncRv1364Ac	1535716	1535417	-	Intergenic	22072964
ncRv11435c	1612987	1613047	+	Intergenic	23284830
ncRv11534A	1735693	1735747	+	Intergenic	19555452;<br>22072964

ncRv11689c	1915190	1914962	-	Intergenic	19555452
ncRv1734A	1960667	1960783	+	Intergenic	24244498
ncRv11846Ac	2096839	2096768	+	Intergenic	29871950
ncRv2165Ac	2429373	2429342	-	5' UTR	20181675
ncRv11414Ac	2641081	2641126	-	5'/3' UTRs	20181675
ncRv2395A	2692172	2692521	+	Intergenic	20181675
ncRv12560A	2881252	2881320	+	Intergenic	20181675
ncRv12562A	2882185	2882276	+	Intergenic	24348997
ncRv12765Ac	3075497	3075437	-	Intergenic	24244498
ncRv12904A	3214341	3214399	+	Intergenic	23284830
ncRv13003Ac	3363153	3363023	-	5' UTR	20181675
ncRv13241Ac	3621466	3621265	-	5' UTR	23284830
ncRv3418Ac	3837458	3837288	-	5' UTR	23284830
ncRv13596A	4040879	4040938	+	Intergenic	20181675
ncRv13651A	4093468	4093522	+	Intergenic	20181675
ncRv13660Ac	4099478	4099386	-	Intergenic	20181675; 22072964
ncRv13661A	4100669	4100968	+	Intergenic	20181675
ncRv13722Ac	4168281	4168224	-	Intergenic	19555452; 20181675
ncRv13778c	4224925	4224970	+	Intergenic	22452820
ncRv13843A	4317073	4317165	+	Intergenic	22072964
