# Supplementary material for: Genome-wide identification of the context-dependent sRNA expression in Mycobacterium tuberculosis
Source: BMC Genomics. 2020 Feb 18;21:167. doi: 10.1186/s12864-020-6573-5 (PMC7029489; doi:10.1186/s12864-020-6573-5)
Supplement: Supplementary file 9 — Additional file 9 Table S3. Highly expressed and less expressed genes in the mid-exponential growth phase. List of highly expressed (a) and less expressed genes (b) protein coding genes in the mid-exponential growth phase with their RPKM values and the gene coordinates. [file 12864_2020_6573_MOESM9_ESM.pdf]

Table S3. Highly expressed and less expressed genes in the mid-exponential growth phase

Table S3a. Highly Expressed genes in the Mid -Exponential growth phase

Gene	Start	End	RPKM
Rv0005	5240	7267	451.269
Rv0006	7302	9818	505.753
Rv0007	9914	10828	430.81
Rv0009	12468	13016	1362.198
Rv0010c	13133	13558	414.943
Rv0015c	17467	18762	507.553
Rv0019c	23270	23737	386.048
Rv0020c	23861	25444	1035.44
Rv0036c	39056	39829	318.757
Rv0038	41304	41912	290.418
Rv0039c	42004	42351	410.437
Rv0040c	42433	43365	626.097
Rv0042c	46581	47207	252.468
Rv0046c	50021	51124	1000.696
Rv0049	52831	53244	551.582
Rv0053	58192	58482	863.449
Rv0054	58586	59080	688.09
Rv0055	59122	59376	975.18
Rv0056	59409	59867	1059.778
Rv0061c	65012	65350	491.461
Rv0062	65552	66694	403.378
Rv0078B	87798	88004	275.267
Rv0079	88204	89025	558.208
Rv0080	89022	89480	316.479
Rv0088	96927	97601	262.179
Rv0108c	130895	131104	1677.835
Rv0125	151148	152215	553.731
Rv0129c	156578	157600	1405.543
Rv0141c	167271	167681	275.356
Rv0144	170284	171126	331.18
Rv0145	171215	172168	785.645
Rv0147	173238	174758	256.539
Rv0148	174833	175693	316.295
Rv0156	184723	185055	408.65
Rv0157	185052	186479	626.256
Rv0157A	186495	186623	632.294
Rv0164	193626	194111	754.272
Rv0167	196861	197658	420.073
Rv0168	197660	198529	249.284
Rv0169	198534	199898	288.468
Rv0171	200932	202479	449.451
Rv0172	202476	204068	361.241
Rv0173	204065	205237	312.897
Rv0174	205231	206778	678.222
Rv0175	206814	207455	1229.419
Rv0176	207452	208420	260.387
Rv0177	208417	208971	427.053
Rv0178	208938	209672	505.581
Rv0179c	209703	210812	270.774
Rv0184	214969	215718	330.403
Rv0185	215715	216224	302.114
Rv0186	216269	218344	267.223
Rv0186A	218390	218551	1099.789
Rv0188	219486	219917	515.27
Rv0189c	219996	221723	250.289
Rv0192	223607	223909	266.99
Rv0198c	234516	236507	283.367
Rv0201c	237892	238395	509.116
Rv0203	241514	241924	270.02
Rv0204c	241976	243214	433.584
Rv0206c	244484	247318	350.711
Rv0207c	247384	248112	331.227
Rv0211	251782	253602	702.982
Rv0220	262812	264023	391.225
Rv0227c	271574	272839	585.911
Rv0236A	286898	287071	284.93
Rv0237	287186	288352	571.921
Rv0239	289104	289337	473.542
Rv0241c	289812	290654	495.691
Rv0242c	290665	292029	1202.787
Rv0243	292171	293493	435.938
Rv0247c	298116	298862	484.145
Rv0248c	298863	300803	323.398
Rv0249c	300834	301655	418.417
Rv0250c	301735	302028	377.46
Rv0257	309699	310073	393.219
Rv0270	324567	326249	291.953
Rv0275c	330933	331658	543.231

Table S3b. Less Expressed genes in the Mid-Exponential growth phase

Gene	Start	End	RPKM
Rv0003	3280	4437	59.809
Rv0022c	27023	27442	9.015
Rv0026	29722	31068	55.745
Rv0032	34295	36610	36.534
Rv0064	68620	71559	59.631
Rv0064A	71589	71828	56.112
Rv0067c	74629	75198	41.403
Rv0068	75301	76212	33.637
Rv0069c	76237	77622	47.785
Rv0070c	77619	78896	36.634
Rv0071	79486	80193	50.646
Rv0077c	85636	86466	42.521
Rv0083	90400	92322	54.32
Rv0085	93289	93951	39.131
Rv0086	93951	95417	49.429
Rv0087	95414	96892	24.62
Rv0090	98480	99250	16.836
Rv0094c	103710	104663	17.449
Rv0095c	104805	105215	11.884
Rv0096	105324	106715	6.09
Rv0097	106734	107603	28.061
Rv0098	107600	108151	22.919
Rv0099	108156	109778	31.046
Rv0100	109783	110019	49.343
Rv0101	110001	117539	33.865
Rv0104	122317	123831	30.323
Rv0105c	123980	124264	3.384
Rv0106	124374	125570	34.33
Rv0109	131382	132872	26.326
Rv0110	133020	133769	43.449
Rv0112	136289	137245	56.953
Rv0114	137941	138513	47.32
Rv0115	138513	139673	28.344
Rv0117	141200	142144	35.042
Rv0118c	142128	143876	56.492
Rv0121c	147908	148342	57.886
Rv0122	148491	148859	47.193
Rv0124	149533	150996	30.555
Rv0131c	158315	159658	49.912
Rv0132c	159700	160782	31.792
Rv0138	165323	165826	58.255
Rv0151c	177543	179309	22.744
Rv0152c	179319	180896	22.918
Rv0159c	187433	188839	40.601
Rv0160c	188931	190439	7.04
Rv0181c	212277	213011	49.179
Rv0193c	224724	226571	22.968
Rv0194	226878	230462	13.24
Rv0195	230899	231534	10.489
Rv0209	249038	250123	57.104
Rv0210	250120	251598	33.406
Rv0213c	254637	255950	44.958
Rv0214	256064	257677	57.007
Rv0216	258913	259926	41.569
Rv0218	260924	262252	25.381
Rv0219	262254	262802	19.374
Rv0224c	267863	268627	47.979
Rv0252	302866	305427	31.655
Rv0253	305453	305809	40.37
Rv0254c	305825	306349	49.138
Rv0259c	310774	311517	12.695
Rv0260c	311514	312659	4.978
Rv0261c	312759	314168	4.553
Rv0266c	317525	321154	53.023
Rv0278c	333437	336310	58.286
Rv0294	358171	358956	44.418
Rv0304c	366150	372764	9.036
Rv0305c	372820	375711	36.855
Rv0306	375914	376585	52.43
Rv0311	379172	380401	40.228
Rv0318c	386305	387099	33.245
Rv0319	387148	387816	57.283
Rv0320	387888	388550	34.27
Rv0322	389260	390591	17.884
Rv0323c	390580	391251	50.532
Rv0324	391352	392032	33.395
Rv0325	392039	392263	40.112
Rv0326	392273	392728	20.337

Table S3. Highly expressed and less expressed genes in the mid-exponential growth phase

Rv0276	331748	332668	305.267
Rv0279c	336560	339073	339.157
Rv0281	340998	341906	619.582
Rv0282	342130	344025	539.969
Rv0283	344022	345638	365.603
Rv0284	345635	349627	1176.412
Rv0285	349624	349932	771.572
Rv0286	349935	351476	3604.871
Rv0287	351525	351818	4954.87
Rv0288	351848	352138	1770.43
Rv0289	352149	353036	673.861
Rv0290	353083	354501	727.39
Rv0291	354498	355883	439.97
Rv0292	355880	356875	373.926
Rv0297	361334	363109	1704.991
Rv0298	363252	363479	1139.244
Rv0299	363476	363778	750.635
Rv0300	363826	364047	429.48
Rv0308	377113	377829	593.465
Rv0309	377931	378587	590.453
Rv0313	382490	382876	320.614
Rv0314c	382879	383541	972.023
Rv0315	383602	384486	338.034
Rv0316	384535	385149	388.765
Rv0338c	403193	405841	265.636
Rv0341	409362	410801	1160.808
Rv0350	419835	421712	1118.381
Rv0351	421709	422416	691.587
Rv0352	422452	423639	309.917
Rv0353	423639	424019	391.772
Rv0363c	441265	442299	958.398
Rv0379	455977	456192	277.057
Rv0380c	456268	456819	589.794
Rv0383c	458461	459315	313.998
Rv0397A	476394	476642	341.008
Rv0423c	508582	510225	378.913
Rv0424c	510377	510652	362.261
Rv0426c	515373	515816	611.174
Rv0430	518733	519041	394.494
Rv0431	519073	519567	560.296
Rv0432	519600	520322	8347.213
Rv0440	528608	530230	337.827
Rv0441c	530296	530724	333.184
Rv0443	532396	532911	255.021
Rv0444c	533091	533789	428.03
Rv0455c	545375	545821	559.621
Rv0460	551749	551988	300.504
Rv0461	552026	552550	508.385
Rv0462	552614	554008	331.682
Rv0463	554016	554309	258.054
Rv0464c	554313	554885	306.02
Rv0466	556458	557252	345.487
Rv0467	557527	558813	318.374
Rv0469	559888	560748	407.372
Rv0470c	560848	561711	872.591
Rv0475	565797	566396	606.286
Rv0476	566508	566771	326.498
Rv0477	566776	567222	261.74
Rv0479c	567921	568967	842.977
Rv0483	571710	573065	324.729
Rv0484c	573046	573801	254.236
Rv0485	573984	575300	252.714
Rv0486	575348	576790	307.209
Rv0497	587377	588309	1054.957
Rv0500A	591111	591347	327.099
Rv0501	591654	592784	379.845
Rv0502	592791	593867	387.109
Rv0503c	593871	594779	253.909
Rv0504c	594802	595302	599.349
Rv0509	600441	601847	633.291
Rv0510	601857	602786	617.272
Rv0511	602819	604516	272.101
Rv0512	604602	605591	341.467
Rv0513	605604	606152	312.393
Rv0514	606149	606448	440.778
Rv0516c	608059	608535	420.276
Rv0524	614835	616223	288.617
Rv0525	616223	616831	375.985
Rv0530	620907	622124	715.099
Rv0530A	622121	622282	252.901

Rv0327c	392696	394045	15.641
Rv0328	394111	394713	56.992
Rv0329c	394694	395320	28.957
Rv0330c	395347	396087	25.432
Rv0331	396201	397367	39.143
Rv0339c	405950	408448	39.915
Rv0344c	414381	414941	49.133
Rv0347	417304	418290	59.573
Rv0355c	424777	434679	16.18
Rv0362	439871	441253	58.983
Rv0366c	444222	444815	33.583
Rv0368c	445314	446525	11.129
Rv0369c	446531	447046	17.447
Rv0370c	447147	448043	6.386
Rv0371c	448040	448633	4.636
Rv0372c	448630	449385	5.27
Rv0373c	449404	451803	13.509
Rv0374c	451800	452279	9.799
Rv0375c	452294	453154	11.461
Rv0376c	453230	454372	25.933
Rv0377	454421	455386	45.281
Rv0378	455637	455858	16.081
Rv0385	462135	463307	36.082
Rv0386	463411	466668	43.597
Rv0395	474940	475344	3.817
Rv0396	475350	475742	9.693
Rv0397	475816	476184	28.607
Rv0399c	477327	478556	53.286
Rv0401	479789	480160	47.594
Rv0402c	480355	483231	43.327
Rv0416	502167	502373	28.319
Rv0417	502366	503124	40.574
Rv0442c	530751	532214	58.131
Rv0447c	535224	536507	32.124
Rv0448c	536504	537169	29.88
Rv0449c	537229	538548	55.446
Rv0452	542142	542852	54.241
Rv0453	543174	544730	25.827
Rv0456c	545889	546803	44.849
Rv0456A	547076	547357	27.044
Rv0456B	547344	547517	33.462
Rv0470A	561854	562294	25.653
Rv0488	577664	578269	15.48
Rv0493c	583701	584690	56.612
Rv0494	584695	585423	52.838
Rv0507	597199	600105	56.078
Rv0535	626457	627251	41.718
Rv0540	632372	633034	48.011
Rv0551c	641096	642811	44.689
Rv0552	642889	644493	51.711
Rv0553	644490	645470	39.616
Rv0561c	651529	652755	39.178
Rv0565c	656010	657470	42.391
Rv0570	661295	663373	59.137
Rv0571c	663487	664818	36.759
Rv0573c	665851	667242	17.306
Rv0574c	667252	668394	32.098
Rv0575c	668579	669745	45.22
Rv0584	679229	681862	17.623
Rv0585c	681885	684272	21.93
Rv0589	686821	688035	50.244
Rv0591	689059	690504	11.323
Rv0592	690501	692027	10.883
Rv0593	692024	693232	22.444
Rv0594	693237	694787	31.479
Rv0595c	694839	695231	39.742
Rv0596c	695228	695485	53.225
Rv0600c	697904	698410	25.002
Rv0601c	698524	698994	8.084
Rv0602c	699038	699799	8.899
Rv0603	699856	700167	23.142
Rv0607	702813	703199	29.242
Rv0610c	704752	705909	45.011
Rv0614	709356	710348	49.344
Rv0620	712716	713807	1.721
Rv0621	714202	715266	31.531
Rv0622	715370	716317	11.825
Rv0629c	720005	721732	43.408
Rv0630c	721729	725013	31.213
Rv0631c	725013	728306	27.184

Table S3. Highly expressed and less expressed genes in the mid-exponential growth phase

Rv0539	631743	632375	441.103
Rv0543c	635573	635875	626.959
Rv0544c	635935	636213	792.816
Rv0545c	636210	637463	303.393
Rv0555	646298	647962	331.648
Rv0558	649689	650393	482.6
Rv0559c	650407	650745	264.863
Rv0562	652771	653778	301.52
Rv0568	659450	660868	328.918
Rv0577	671166	671951	624.585
Rv0581	677710	677925	313.803
Rv0597c	695668	696903	296.41
Rv0598c	697154	697567	400.711
Rv0599c	697564	697800	723.175
Rv0608	703244	703489	344.242
Rv0609	703486	703887	392.987
Rv0613c	706948	709515	573.05
Rv0626	718025	718285	309.127
Rv0627	718282	718689	656.771
Rv0632c	728583	729278	938.037
Rv0634A	731113	731364	729.692
Rv0634B	731712	731879	564.978
Rv0635	731930	732406	1162.104
Rv0636	732393	732821	712.958
Rv0637	732825	733325	834.459
Rv0638	733737	734222	1150.68
Rv0639	734254	734970	1673.153
Rv0640	735022	735450	2057.509
Rv0641	735517	736224	600.74
Rv0642c	736298	737203	764.485
Rv0651	748276	748812	1621.886
Rv0652	748849	749241	808.244
Rv0653c	749234	749929	636.501
Rv0655	751517	752596	1196.296
Rv0659c	754685	754993	1730.072
Rv0660c	754980	755225	1562.556
Rv0667	759807	763325	1334.608
Rv0668	763370	767320	418.215
Rv0670	769792	770550	970.424
Rv0682	781560	781934	1589.216
Rv0683	781934	782404	963.129
Rv0684	782485	784590	2688.884
Rv0685	784821	786011	1493.52
Rv0692	791831	792160	576.394
Rv0693	792157	793332	329.22
Rv0694	793335	794525	2950.209
Rv0700	800487	800792	2323.598
Rv0701	800809	801462	2258.901
Rv0702	801462	802133	1892.893
Rv0703	802133	802435	1699.114
Rv0704	802528	803370	2436.676
Rv0705	803411	803692	1610.001
Rv0706	803689	804282	1786.928
Rv0707	804282	805106	1799.914
Rv0708	805110	805526	2576.045
Rv0709	805526	805759	1742.247
Rv0710	805756	806166	806.1
Rv0714	811373	811741	1217.892
Rv0715	811742	812059	1219.31
Rv0716	812059	812622	1111.518
Rv0717	812627	812812	1697.454
Rv0718	812976	813374	1577.437
Rv0719	813398	813937	1404.48
Rv0720	813940	814308	1903.665
Rv0721	814328	814990	1908.132
Rv0722	814993	815190	2700.682
Rv0723	815190	815630	498.021
Rv0724	815663	817534	328.636
Rv0730	822866	823594	322.144
Rv0732	824800	826125	825.189
Rv0733	826122	826667	553.862
Rv0734	826670	827470	287.087
Rv0748	840947	841204	294.326
Rv0756c	850741	851466	263.341
Rv0757	851608	852351	249.768
Rv0759c	853825	854157	353.298
Rv0760c	854267	854686	547.415
Rv0761c	854699	855826	293.932
Rv0787A	882524	882763	265.594
Rv0788	882760	883434	626.16

Rv0648	742719	746366	12.325
Rv0649	746363	747037	19.267
Rv0650	747037	747945	16.468
Rv0656c	752984	753367	27.954
Rv0658c	753693	754409	47.168
Rv0661c	755335	755772	21.277
Rv0665	758801	759139	44.173
Rv0666	759136	759309	24.289
Rv0687	787099	787926	49.891
Rv0697	796933	798372	12.907
Rv0698	798833	799444	57.177
Rv0699	799629	799850	5.057
Rv0711	806335	808698	54.804
Rv0725c	817539	818444	50.506
Rv0728c	820496	821476	41.783
Rv0736	828140	828892	30.228
Rv0737	829207	829704	21.272
Rv0739	830855	831661	19.523
Rv0743c	833886	834443	52.648
Rv0745	835154	835681	12.766
Rv0752c	843242	844414	43.615
Rv0753c	844421	845953	54.9
Rv0754	846159	847913	15.687
Rv0755c	848103	850040	54.58
Rv0762c	855925	856470	38.674
Rv0763c	856473	856679	35.371
Rv0764c	856682	858037	35.003
Rv0765c	858037	858864	30.513
Rv0766c	858864	860072	31.288
Rv0767c	860069	860710	42.331
Rv0768	860912	862381	27.159
Rv0769	862412	863158	46.71
Rv0770	863256	864143	23.964
Rv0771	864140	864574	33.424
Rv0776c	868984	869763	34.287
Rv0790c	884072	884800	35.553
Rv0791c	884797	885840	43.074
Rv0792c	885837	886646	47.023
Rv0793	886719	887024	20.592
Rv0797	890388	891482	36.04
Rv0807	901635	902024	36.428
Rv0816c	909611	910033	59.003
Rv0817c	910030	910842	37.078
Rv0826	919634	920689	34.618
Rv0828c	921191	921613	5.667
Rv0835	930953	931597	28.807
Rv0836c	932279	932932	32.825
Rv0837c	933003	934031	14.676
Rv0838	934720	935490	43.367
Rv0839	935577	936389	58.135
Rv0840c	936457	937317	21.052
Rv0841	937593	937835	19.765
Rv0842	938112	939404	59.009
Rv0843	939388	940392	28.006
Rv0845	941190	942467	38.261
Rv0846c	942680	944194	30.294
Rv0848	944938	946056	58.797
Rv0849	946056	947315	23.008
Rv0851c	947641	948468	34.648
Rv0862c	960342	962612	43.688
Rv0878c	976872	978203	48.867
Rv0881	979362	980228	45.399
Rv0886	983803	985530	58.615
Rv0887c	985513	985971	43.238
Rv0891c	992598	993455	43.581
Rv0893c	995318	996295	38.525
Rv0894	996524	997705	17.923
Rv0895	997782	999299	20.259
Rv0899	1002812	1003792	50.86
Rv0913c	1017217	1018725	50.083
Rv0915c	1020058	1021329	24.549
Rv0916c	1021344	1021643	23.996
Rv0917	1022087	1023868	23.744
Rv0918	1024211	1024687	58.554
Rv0920c	1025497	1026816	49.278
Rv0924c	1030578	1031864	56.034
Rv0943c	1052696	1053736	12.787
Rv0944	1053765	1054241	18.407
Rv0953c	1064114	1064962	30.074
Rv0962c	1074440	1075114	17.742

Rv0805	898831	899787	258.775
Rv0809	903725	904819	1625.373
Rv0810c	904905	905087	361.859
Rv0813c	907338	908018	1105.421
Rv0814c	908181	908483	345.276
Rv0815c	908485	909318	287.447
Rv0818	910972	911739	340.428
Rv0821c	913558	914199	319.819
Rv0823c	916477	917646	1397.168
Rv0824c	917734	918750	565.882
Rv0831c	922894	923709	295.22
Rv0832	924951	925364	272.03
Rv0833	925361	927610	246.614
Rv0834c	927837	930485	450.069
Rv0859	955077	956288	445.886
Rv0860	956293	958455	859.287
Rv0863	962599	962880	850.9
Rv0864	962890	963393	263.534
Rv0866	963869	964294	892.713
Rv0867c	964312	965535	521.601
Rv0871	967898	968305	290.703
Rv0873	970505	972457	298.252
Rv0877	976075	976863	350.335
Rv0883c	980506	981267	276.668
Rv0889c	988740	989861	746.961
Rv0896	999472	1000767	481.273
Rv0898c	1002441	1002704	381.103
Rv0903c	1005852	1006562	365.936
Rv0905	1008207	1008938	316.538
Rv0909	1014681	1014860	404.716
Rv0922	1027685	1029337	254.779
Rv0925c	1031896	1032633	697.37
Rv0931c	1037920	1039914	1003.853
Rv0932c	1039936	1041048	498.368
Rv0934	1042115	1043239	343.869
Rv0935	1043299	1044315	414.706
Rv0950c	1060656	1061654	790.517
Rv0951	1061964	1063127	745.219
Rv0952	1063140	1064051	1125.999
Rv0954	1065127	1066038	286.003
Rv0955	1066078	1067445	276.196
Rv0957	1068205	1069776	1022.082
Rv0979A	1094886	1095059	311.516
Rv0980c	1095078	1096451	404.55
Rv0983	1099066	1100460	693.666
Rv0984	1100460	1101005	266.593
Rv0985c	1101025	1101480	278.784
Rv0991c	1108172	1108504	290.024
Rv0994	1110269	1111549	428.889
Rv0996	1112384	1113460	419.642
Rv0997	1114293	1114724	344.268
Rv1004c	1120889	1122148	269.802
Rv1006	1123714	1125417	534.908
Rv1009	1128091	1129179	263.312
Rv1010	1129152	1130105	390.776
Rv1013	1131625	1133259	340.553
Rv1015c	1133921	1134568	309.427
Rv1017c	1135501	1136481	2476.115
Rv1072	1196279	1197115	407.6
Rv1073	1197231	1198082	431.688
Rv1074c	1198156	1199373	270.715
Rv1077	1201717	1203111	1816.853
Rv1078	1203313	1204035	496.825
Rv1079	1204067	1205233	1288.134
Rv1080c	1205304	1205798	353.376
Rv1093	1220574	1221854	1836.823
Rv1094	1221959	1222786	499.972
Rv1095	1222997	1224298	371.775
Rv1097c	1225263	1226144	305.414
Rv1098c	1226141	1227565	300.385
Rv1099c	1227596	1228684	405.699
Rv1102c	1230660	1230971	494.939
Rv1103c	1230971	1231291	505.389
Rv1109c	1235457	1236095	250.014
Rv1110	1236185	1237192	267.661
Rv1117	1241633	1241956	329.531
Rv1132	1257325	1259055	1952.29
Rv1133c	1259067	1261346	281.502
Rv1152	1279655	1280020	738.998
Rv1156	1282306	1282893	520.512
Rv0963c	1075297	1076097	7.152
Rv0964c	1076196	1076678	30.605
Rv0972c	1082584	1083750	35.635
Rv0973c	1083747	1085750	19.238
Rv0974c	1085756	1087345	13.124
Rv0975c	1087348	1088496	14.797
Rv0976c	1088493	1090175	27.255
Rv0977	1090373	1093144	52.115
Rv0978c	1093361	1094356	24.508
Rv0979c	1094670	1094864	51.385
Rv0987	1102542	1105109	47.593
Rv0988	1105116	1106276	54.915
Rv1003	1120022	1120879	50.049
Rv1012	1131128	1131421	36.159
Rv1027c	1148427	1149107	17.487
Rv1028c	1149104	1151686	53.783
Rv1028A	1151920	1152012	50.776
Rv1029	1152012	1153727	34.094
Rv1030	1153724	1155853	31.716
Rv1031	1155853	1156422	48.041
Rv1032c	1156426	1157955	34.207
Rv1033c	1157963	1158736	48.779
Rv1039c	1161297	1162472	27.335
Rv1040c	1162549	1163376	16.432
Rv1044	1167053	1167676	28.988
Rv1045	1167673	1168554	27.273
Rv1048c	1171038	1172153	23.333
Rv1049	1172386	1172832	31.171
Rv1050	1172881	1173786	31.712
Rv1051c	1173945	1174700	10.095
Rv1056	1177628	1178392	53.918
Rv1057	1179396	1180577	31.984
Rv1058	1180684	1182315	44.963
Rv1064c	1186904	1187323	27.045
Rv1068c	1190757	1192148	32.073
Rv1088	1214513	1214947	11.433
Rv1112	1238255	1239328	24.184
Rv1115	1240187	1240885	23.024
Rv1120c	1243010	1243504	20.044
Rv1125	1248082	1249326	44.684
Rv1128c	1251617	1252972	16
Rv1129c	1253074	1254534	20.483
Rv1134	1261922	1262158	17.332
Rv1135c	1262272	1264128	25.783
Rv1138c	1265472	1266488	32.911
Rv1139c	1266485	1266985	58.873
Rv1143	1270062	1271144	55.589
Rv1145	1272423	1273334	16.586
Rv1146	1273355	1274767	27.686
Rv1147	1274900	1275550	51.889
Rv1151c	1278904	1279617	40.408
Rv1154c	1280843	1281484	48.608
Rv1169c	1299822	1300124	55.064
Rv1187	1329390	1331021	44.003
Rv1188	1331021	1332010	25.221
Rv1189	1332092	1332964	17.276
Rv1190	1332980	1333858	26.068
Rv1203c	1346321	1346905	52.625
Rv1236	1378927	1379850	41.506
Rv1243c	1384989	1386677	24.814
Rv1250	1394179	1395918	52.491
Rv1256c	1403386	1404603	56.016
Rv1258c	1406081	1407340	39.909
Rv1263	1410431	1411819	50.145
Rv1267c	1416181	1417347	32.147
Rv1268c	1417658	1418356	16.555
Rv1272c	1420410	1422305	53.442
Rv1273c	1422302	1424050	28.885
Rv1282c	1435268	1436143	59.901
Rv1285	1437909	1438907	30.615
Rv1286	1438907	1440751	37.527
Rv1288	1441348	1442718	35.892
Rv1290c	1443482	1445047	44.16
Rv1290A	1445058	1445372	21.853
Rv1291c	1445499	1445834	28.289
Rv1329c	1497195	1499189	41.698
Rv1330c	1499213	1500559	41.531
Rv1347c	1511973	1512605	31.9
Rv1348	1513047	1515626	32.162
Rv1349	1515623	1517362	33.006

Table S3. Highly expressed and less expressed genes in the mid-exponential growth phase

Rv1157c	1283056	1284171	782.389		Rv1350	1517491	1518234	30.443
Rv1158c	1284179	1284862	318.819		Rv1353c	1519200	1519985	35.933
Rv1159A	1286284	1286568	283.097		Rv1354c	1520005	1521876	40.079
Rv1162	1291065	1292741	248.687		Rv1355c	1521885	1524032	13.232
Rv1172c	1301755	1302681	424.151		Rv1356c	1524029	1524820	37.44
Rv1173	1302931	1305501	1814.687		Rv1357c	1525293	1526216	24.361
Rv1174c	1305669	1306001	1225.475		Rv1358	1526612	1530091	6.495
Rv1177	1309005	1309331	596.887		Rv1359	1530173	1530925	41.552
Rv1178	1309364	1310452	250.607		Rv1366	1538390	1539211	37.325
Rv1182	1320035	1321453	317.899		Rv1371	1543359	1544828	1.232
Rv1185c	1325776	1327512	650.914		Rv1372	1544825	1546006	26.458
Rv1192	1334927	1335754	268.815		Rv1373	1546012	1546992	36.948
Rv1194c	1337248	1338513	2043.301		Rv1376	1549148	1550641	53.4
Rv1195	1339003	1339302	2288.627		Rv1380	1553232	1554191	57.078
Rv1196	1339349	1340524	3804.457		Rv1393c	1568109	1569587	34.616
Rv1197	1340659	1340955	5125.345		Rv1394c	1569584	1570969	38.199
Rv1198	1341006	1341290	402.733		Rv1395	1571047	1572081	34.348
Rv1199c	1341358	1342605	448.853		Rv1403c	1579598	1580422	42.804
Rv1201c	1344216	1345169	257.833		Rv1414	1589891	1590292	34.948
Rv1207	1351191	1352147	304.17		Rv1429	1604878	1606146	23.162
Rv1209	1353157	1353525	2382.222		Rv1431	1608083	1609852	42.049
Rv1211	1354243	1354470	344.742		Rv1432	1609849	1611270	23.279
Rv1212c	1354498	1355661	349.086		Rv1439c	1616961	1617386	33.869
Rv1220c	1363503	1364150	422.186		Rv1450c	1630638	1634627	32.588
Rv1221	1364413	1365186	402.947		Rv1452c	1636004	1638229	31.744
Rv1222	1365344	1365808	380.731		Rv1453	1638381	1639646	53.272
Rv1223	1365875	1367461	301.646		Rv1455	1640680	1641543	48.276
Rv1229c	1371777	1372949	482.275		Rv1482c	1672457	1673299	17.228
Rv1233c	1376230	1376826	257.823		Rv1490	1679322	1680629	32.885
Rv1234	1376976	1377503	782.088		Rv1492	1682157	1684004	54.42
Rv1240	1383213	1384202	435.332		Rv1493	1684005	1686257	46.995
Rv1241	1384278	1384538	263.454		Rv1499	1690407	1690805	46.612
Rv1248c	1389357	1393052	503.925		Rv1502	1692924	1693823	55.443
Rv1274	1424197	1424754	454.117		Rv1505c	1695281	1695946	43.761
Rv1275	1424751	1425293	267.624		Rv1506c	1695943	1696443	36.343
Rv1294	1449375	1450700	284.802		Rv1508A	1699866	1700228	45.69
Rv1295	1450697	1451779	1161.47		Rv1509	1700212	1701093	28.947
Rv1297	1453204	1455012	1760.386		Rv1515c	1706630	1707526	58.547
Rv1298	1455163	1455405	306.751		Rv1516c	1707529	1708539	58.578
Rv1299	1455495	1456568	259.635		Rv1517	1708871	1709635	25.599
Rv1300	1456565	1457542	324.486		Rv1518	1709644	1710603	11.677
Rv1301	1457558	1458211	341.443		Rv1519	1710733	1711002	26.423
Rv1303	1459766	1460251	368.23		Rv1520	1711028	1712068	42.983
Rv1304	1460244	1460996	2809.315		Rv1522c	1714172	1717612	50.498
Rv1305	1461045	1461290	2170.147		Rv1524	1718726	1719970	57.513
Rv1306	1461321	1461836	1098.51		Rv1526c	1720780	1722060	32.758
Rv1307	1461843	1463183	1020.902		Rv1528c	1728953	1729450	7.645
Rv1308	1463228	1464877	661.224		Rv1529	1729502	1731256	35.859
Rv1309	1464884	1465801	962.034		Rv1530	1731373	1732476	27.377
Rv1310	1465841	1467301	679.353		Rv1537	1739856	1741247	39.721
Rv1311	1467315	1467680	284.359		Rv1542c	1744426	1744836	22.46
Rv1312	1467688	1468131	460.238		Rv1548c	1751297	1753333	23.8
Rv1322	1484982	1485278	1672.044		Rv1550	1753716	1755431	26.717
Rv1322A	1485313	1485771	694.187		Rv1551	1755445	1757310	7.922
Rv1323	1485862	1487031	430.028		Rv1552	1757681	1759432	3.14
Rv1324	1487161	1488075	277.453		Rv1553	1759435	1760178	3.309
Rv1328	1494564	1497155	730.114		Rv1554	1760175	1760555	2.706
Rv1331	1500661	1500966	428.284		Rv1555	1760552	1760929	10.435
Rv1332	1500926	1501582	363.266		Rv1556	1760997	1761605	50.18
Rv1335	1503103	1503384	308.675		Rv1557	1761744	1762937	27.916
Rv1337	1504356	1505078	250.655		Rv1562c	1765400	1767142	26.585
Rv1340	1506755	1507534	252.972		Rv1563c	1767135	1769432	19.656
Rv1352	1518763	1519134	442.459		Rv1564c	1769436	1771601	41.44
Rv1361c	1532443	1533633	441.363		Rv1573	1779314	1779724	13.956
Rv1374c	1547072	1547530	291.604		Rv1574	1779930	1780241	10.78
Rv1379	1552654	1553235	1072.711		Rv1575	1780199	1780699	37.192
Rv1386	1561464	1561772	1364.402		Rv1576c	1780643	1782064	55.65
Rv1387	1561769	1563388	2365.083		Rv1579c	1783309	1783623	40.93
Rv1388	1563694	1564266	2568.1		Rv1581c	1783906	1784301	36.95
Rv1389	1564401	1565027	1126.984		Rv1582c	1784497	1785912	25.921
Rv1390	1565093	1565425	394.046		Rv1586c	1787096	1788505	44.004
Rv1391	1565441	1566697	527.79		Rv1587c	1788162	1789163	25.321
Rv1392	1566825	1568036	803.416		Rv1588c	1789168	1789836	31.519
Rv1397c	1574112	1574513	3249.791		Rv1619	1818575	1820029	37.385
Rv1398c	1574510	1574767	498.496		Rv1620c	1819963	1821693	29.367
Rv1404	1580591	1581073	314.496		Rv1621c	1821690	1823273	48.684
Rv1409	1585194	1586213	590.985		Rv1622c	1823360	1824400	46.4
Rv1410c	1586210	1587766	622.937		Rv1645c	1854399	1855454	44.194
Rv1411c	1587772	1588482	967.3		Rv1661	1875304	1881684	17.303
Rv1434	1612256	1612393	1210.971		Rv1662	1881704	1886512	10.706

Table S3. Highly expressed and less expressed genes in the mid-exponential growth phase

Rv1435c	1612342	1612950	760.313	Rv1663	1886512	1888020	11.368
Rv1436	1613307	1614326	350.397	Rv1664	1888026	1891079	10.506
Rv1437	1614329	1615567	349.732	Rv1666c	1892270	1893562	41.588
Rv1438	1615564	1616349	322.413	Rv1669	1895725	1896087	8.459
Rv1440	1617837	1618070	286.656	Rv1670	1896120	1896467	53.785
Rv1441c	1618209	1619684	375.045	Rv1671	1896475	1896867	31.417
Rv1448c	1626959	1628080	417.633	Rv1673c	1898300	1899232	16.596
Rv1449c	1628097	1630199	268.142	Rv1674c	1899260	1899916	12.573
Rv1451	1635029	1635955	290.603	Rv1675c	1900241	1900975	8.739
Rv1462	1649526	1650719	283.413	Rv1681	1905250	1906242	53.016
Rv1463	1650716	1651516	508.666	Rv1682	1906403	1907320	28.835
Rv1466	1653231	1653578	249.561	Rv1686c	1911401	1912081	56.404
Rv1468c	1655609	1656721	285.375	Rv1687c	1912153	1912920	46.247
Rv1472	1659763	1660620	391.004	Rv1688	1912979	1913590	36.398
Rv1477	1666990	1668408	341.403	Rv1702c	1927211	1928575	25.579
Rv1478	1668419	1669144	575.37	Rv1705c	1931497	1932654	34.715
Rv1479	1669283	1670416	511.521	Rv1706c	1932694	1933878	28.581
Rv1480	1670413	1671366	257.56	Rv1706A	1934482	1934649	8.164
Rv1481	1671377	1672384	371.848	Rv1714	1941853	1942665	41.84
Rv1483	1673440	1674183	640.78	Rv1715	1942659	1943573	21.569
Rv1484	1674202	1675011	279.009	Rv1716	1943576	1944406	36.677
Rv1488	1677397	1678542	267.282	Rv1717	1944406	1944756	45.788
Rv1521	1712302	1714053	1483.228	Rv1719	1945641	1946420	43.698
Rv1535	1735976	1736212	321.776	Rv1720c	1947030	1947419	34.188
Rv1539	1742244	1742852	334.439	Rv1722	1947861	1949345	58.544
Rv1543	1745064	1746089	335.045	Rv1723	1949342	1950589	31.94
Rv1544	1746094	1746897	328.769	Rv1724c	1950632	1951051	23.205
Rv1545	1746919	1747146	390.686	Rv1725c	1951041	1951751	28.616
Rv1559	1763428	1764717	918.239	Rv1726	1951852	1953237	21.657
Rv1566c	1773928	1774620	257.154	Rv1727	1953270	1953839	45.174
Rv1569	1776702	1777862	364.496	Rv1729c	1954631	1955569	40.294
Rv1584c	1786307	1786528	381.376	Rv1730c	1955692	1957245	35.693
Rv1589	1790284	1791333	770.508	Rv1735c	1961291	1961788	10.074
Rv1590	1791334	1791573	1306.276	Rv1742	1968173	1968910	33.33
Rv1594	1794756	1795805	960.776	Rv1750c	1977969	1979567	37.751
Rv1595	1795805	1797388	807.981	Rv1753c	1981614	1984775	20.272
Rv1596	1797388	1798245	259.784	Rv1758	1989042	1989566	8.36
Rv1608c	1807298	1807762	605.23	Rv1759c	1989833	1992577	47.757
Rv1611	1810240	1811058	380.768	Rv1760	1993153	1994661	46.021
Rv1613	1812359	1813171	411.065	Rv1767	2000074	2000433	45.076
Rv1614	1813171	1814577	356.247	Rv1774	2007832	2009172	56.879
Rv1615	1815253	1815693	283.062	Rv1777	2010656	2011960	8.81
Rv1617	1816189	1817607	592.65	Rv1785c	2023447	2024628	59.786
Rv1626	1828180	1828797	374.641	Rv1786	2024828	2025031	54.171
Rv1627c	1828865	1830073	1369.516	Rv1787	2025301	2026398	54.034
Rv1630	1833542	1834987	432.074	Rv1790	2028425	2029477	18.697
Rv1631	1835013	1836236	778.989	Rv1800	2039453	2041420	41.487
Rv1636	1842451	1842891	309.398	Rv1801	2042001	2043272	9.76
Rv1637c	1842898	1843692	363.968	Rv1802	2043384	2044775	25.79
Rv1638A	1846716	1846973	287.583	Rv1803c	2044923	2046842	24.075
Rv1639c	1846989	1848458	1281.332	Rv1804c	2047023	2047349	25.094
Rv1641	1852273	1852878	1713.321	Rv1806	2048072	2048371	35.732
Rv1642	1852928	1853122	1975.005	Rv1814	2056521	2057423	50.823
Rv1643	1853184	1853573	653.901	Rv1817	2059595	2061058	40.058
Rv1644	1853606	1854388	271.167	Rv1834	2079830	2080696	21.345
Rv1676	1901047	1901751	253.164	Rv1835c	2080701	2082587	59.101
Rv1677	1901748	1902296	323.584	Rv1840c	2087971	2089518	49.181
Rv1693	1917756	1917932	734.22	Rv1850	2097961	2099694	56.645
Rv1697	1921542	1922723	288.932	Rv1851	2099694	2100329	32.805
Rv1698	1922745	1923689	362.146	Rv1864c	2111354	2112109	59.327
Rv1703c	1929131	1929721	600.727	Rv1865c	2112106	2112966	50.498
Rv1732c	1959243	1959791	291.665	Rv1866	2113140	2115476	44.329
Rv1738	1965657	1965941	254.744	Rv1867	2115764	2117248	39.039
Rv1751	1979621	1981003	278.935	Rv1868	2117347	2119446	56.086
Rv1770	2003878	2005164	273.279	Rv1873	2123174	2123611	15.037
Rv1779c	2012686	2014479	309.813	Rv1878	2128022	2129374	49.001
Rv1783	2019257	2023432	697.446	Rv1890c	2139076	2139687	23.705
Rv1789	2026790	2027971	1878.837	Rv1895	2142521	2143675	49.254
Rv1791	2029904	2030203	483.796	Rv1897c	2144451	2144882	51.133
Rv1793	2030694	2030978	332.22	Rv1903	2150364	2150768	46.583
Rv1794	2031066	2031968	342.722	Rv1909c	2156149	2156592	59.637
Rv1795	2032240	2033751	291.17	Rv1910c	2156706	2157299	50.771
Rv1796	2033729	2035486	573.981	Rv1913	2159191	2159943	47.853
Rv1797	2035483	2036703	695.523	Rv1914c	2159921	2160328	55.521
Rv1805c	2047687	2048034	319.714	Rv1917c	2162932	2167311	8.259
Rv1810	2052933	2053289	313.589	Rv1921c	2172524	2173795	19.713
Rv1818c	2061178	2062674	627.825	Rv1922	2174067	2175182	44.342
Rv1821	2066457	2068883	1548.506	Rv1928c	2180450	2181217	30.074
Rv1826	2071952	2072356	524.064	Rv1930c	2181918	2182442	15.27
Rv1827	2072596	2073084	495.952	Rv1931c	2182460	2183239	28.233

Table S3. Highly expressed and less expressed genes in the mid-exponential growth phase

Rv1828	2073081	2073824	537.994
Rv1830	2074841	2075518	412.458
Rv1831	2075571	2075828	618.158
Rv1836c	2082603	2084636	286.946
Rv1837c	2084756	2086981	296.024
Rv1843c	2092259	2093698	675.425
Rv1845c	2095218	2096168	391.129
Rv1846c	2096183	2096599	518.332
Rv1856c	2104146	2104823	417.038
Rv1860	2107736	2108713	347.622
Rv1863c	2110591	2111361	3400.194
Rv1870c	2120795	2121430	2836.282
Rv1871c	2121495	2121884	398.006
Rv1872c	2121907	2123151	352.507
Rv1883c	2133231	2133692	1323.935
Rv1885c	2134273	2134872	549.483
Rv1886c	2134890	2135867	315.717
Rv1887	2136258	2137400	353.845
Rv1891	2139741	2140148	564.676
Rv1898	2144940	2145248	262.198
Rv1899c	2145214	2146245	319.336
Rv1904	2150954	2151385	295.665
Rv1906c	2152425	2152895	359.605
Rv1908c	2153889	2156111	264.409
Rv1915	2160463	2161566	1183.304
Rv1919c	2171061	2171525	776.878
Rv1925	2177087	2178949	1053.923
Rv1926c	2178957	2179436	306.074
Rv1932	2183372	2183869	514.892
Rv1933c	2183866	2184957	247.133
Rv1980c	2223343	2224029	332.709
Rv1981c	2224220	2225188	630.641
Rv1987	2231026	2231454	442.313
Rv2007c	2256084	2256428	358.63
Rv2009	2258030	2258272	304.435
Rv2010	2258273	2258671	1076.77
Rv2030c	2276441	2278486	301.051
Rv2031c	2278498	2278932	329.181
Rv2033c	2280240	2281082	537.029
Rv2049c	2307293	2307517	379.803
Rv2050	2307821	2308156	318.117
Rv2059	2316279	2316680	274.113
Rv2060	2316279	2316680	547.36
Rv2061c	2316681	2317085	317.935
Rv2069	2326944	2327501	260.711
Rv2074	2330993	2331406	449.309
Rv2077c	2333323	2334294	739.477
Rv2080	2337306	2337869	1250.953
Rv2081c	2338065	2338505	337.144
Rv2091c	2348558	2349292	261.746
Rv2094c	2353046	2353297	279.515
Rv2097c	2355319	2356677	299.674
Rv2101	2360240	2363281	441.276
Rv2102	2363391	2364107	1111.683
Rv2109c	2368983	2369729	278.69
Rv2110c	2369726	2370601	339.594
Rv2111c	2370598	2370792	342.424
Rv2114	2373834	2374457	287.476
Rv2115c	2374461	2376290	387.281
Rv2128	2390085	2390288	1623.031
Rv2131c	2392517	2393320	269.479
Rv2134c	2394650	2395237	292.229
Rv2137c	2396902	2397315	885.948
Rv2140c	2399798	2400328	1070.113
Rv2142A	2402507	2402722	309.525
Rv2144c	2404165	2404521	1035.522
Rv2145c	2404616	2405398	290.595
Rv2146c	2405666	2405956	661.803
Rv2147c	2406118	2406843	393.685
Rv2149c	2407622	2408374	365.358
Rv2150c	2408385	2409524	402.238
Rv2153c	2412119	2413351	525.815
Rv2154c	2413348	2414922	1119.288
Rv2158c	2419001	2420608	1253.176
Rv2159c	2420631	2421665	349.407
Rv2161c	2422271	2423137	470.101
Rv2162c	2423240	2424838	271.368
Rv2164c	2427084	2428238	283.378
Rv2166c	2429427	2429858	360.856
Rv2169c	2431565	2431969	338.577

Rv1934c	2184959	2186188	16.186
Rv1935c	2186203	2187159	11.246
Rv1936	2187384	2188493	17.857
Rv1937	2188496	2191015	19.725
Rv1938	2191027	2192097	13.975
Rv1939	2192094	2192609	15.754
Rv1940	2192606	2193667	28.46
Rv1945	2195989	2197353	44.162
Rv1946c	2197508	2197960	59.29
Rv1948c	2198714	2199064	24.139
Rv1953	2200938	2201249	54.333
Rv1955	2201719	2202096	42.094
Rv1961	2204212	2204706	46.603
Rv1963c	2205582	2206802	39.737
Rv1964	2207700	2208497	9.423
Rv1965	2208507	2209322	8.008
Rv1966	2209327	2210604	6.038
Rv1967	2210601	2211629	5.196
Rv1968	2211626	2212858	4.209
Rv1969	2212855	2214126	5.838
Rv1970	2214123	2215256	6.254
Rv1971	2215257	2216570	6.009
Rv1972	2216592	2217167	11.351
Rv1973	2217164	2217646	15.024
Rv1974	2217659	2218036	8.952
Rv1975	2218052	2218717	23.326
Rv1977	2219754	2220800	56.647
Rv1985c	2228991	2229902	36.287
Rv1986	2230011	2230610	37.277
Rv1994c	2237628	2237984	54.057
Rv1995	2238141	2238908	11.394
Rv1997	2240159	2242876	28.093
Rv1999c	2243816	2245138	5.732
Rv2000	2245209	2246822	28.199
Rv2001	2246832	2247584	40.957
Rv2003c	2248563	2249420	42.59
Rv2008c	2256617	2257942	20.175
Rv2011c	2258854	2259285	44.236
Rv2012	2259326	2259820	32.577
Rv2017	2263998	2265038	33.29
Rv2019	2265989	2266405	56.63
Rv2022c	2267119	2267724	43.779
Rv2025c	2270750	2271748	47.995
Rv2026c	2271863	2272747	21.416
Rv2027c	2272787	2274508	29.936
Rv2028c	2274569	2275408	26.347
Rv2029c	2275405	2276424	38.802
Rv2036	2282099	2282740	51.083
Rv2037c	2282747	2283721	40.159
Rv2038c	2283723	2284796	13.102
Rv2039c	2284799	2285641	10.326
Rv2040c	2285628	2286530	16.454
Rv2041c	2286527	2287846	21.504
Rv2044c	2289282	2289599	20.095
Rv2056c	2314354	2314659	46.681
Rv2057c	2314661	2314825	49.881
Rv2058c	2314825	2315061	33.906
Rv2071c	2328222	2328977	35.051
Rv2072c	2328974	2330146	50.08
Rv2073c	2330214	2330963	51.864
Rv2077A	2334295	2334594	44.329
Rv2079	2335355	2337325	59.736
Rv2086	2343311	2343916	10.862
Rv2088	2344411	2346180	44.033
Rv2089c	2346197	2347324	17.948
Rv2116	2376571	2377140	44.859
Rv2122c	2380663	2380944	41.759
Rv2123	2381071	2382492	15.163
Rv2170	2432235	2432855	41.386
Rv2176	2437941	2439140	45.299
Rv2177c	2439282	2439947	29.846
Rv2179c	2441811	2442317	50.71
Rv2180c	2442327	2443214	40.091
Rv2191	2453819	2455756	43.941
Rv2205c	2469387	2470463	42.584
Rv2227	2500931	2501632	55.32
Rv2231A	2505736	2506161	39.601
Rv2231B	2506207	2506383	34.035
Rv2249c	2523241	2524791	24.298
Rv2250c	2524785	2525354	23.805

Table S3. Highly expressed and less expressed genes in the mid-exponential growth phase

Rv2172c	2433631	2434536	259.33	Rv2252	2526989	2527918	42.994
Rv2178c	2440332	2441720	362.46	Rv2253	2527984	2528487	8.887
Rv2182c	2444586	2445329	933.669	Rv2254c	2528520	2528975	29.769
Rv2183c	2445415	2445810	904.034	Rv2264c	2536572	2538350	48.901
Rv2185c	2447066	2447500	702.761	Rv2265	2538700	2539929	18.805
Rv2190c	2452115	2453272	741.993	Rv2266	2540104	2541390	30.537
Rv2193	2456901	2457512	882.539	Rv2267c	2541644	2542810	20.607
Rv2194	2457553	2458395	411.831	Rv2268c	2542807	2544276	35.558
Rv2195	2458392	2459681	248.001	Rv2269c	2544289	2544621	37.499
Rv2196	2459678	2461327	651.651	Rv2270	2544698	2545225	9.203
Rv2197c	2461504	2462148	479.27	Rv2274c	2546488	2546805	26.37
Rv2198c	2462148	2463047	468.824	Rv2277c	2549124	2550029	10.546
Rv2199c	2463233	2463652	478.386	Rv2281	2553173	2554831	23.039
Rv2200c	2463660	2464751	1373.342	Rv2282c	2554938	2555876	21.136
Rv2202c	2467053	2468027	380.694	Rv2283	2555941	2556135	12.789
Rv2203	2468231	2468923	332.487	Rv2286c	2558877	2559569	58.139
Rv2204c	2468931	2469287	276.958	Rv2287	2559703	2561331	44.634
Rv2206	2470622	2471332	265.526	Rv2293c	2564292	2565032	39.779
Rv2207	2471411	2472496	344.11	Rv2300c	2572076	2573008	59.116
Rv2208	2472493	2473242	536.818	Rv2304c	2575016	2575225	52.83
Rv2211c	2476042	2477181	288.639	Rv2305	2575809	2577098	55.106
Rv2213	2478338	2479885	311.495	Rv2307c	2577851	2578696	47.929
Rv2215	2481965	2483626	363.504	Rv2307A	2579228	2579419	16.149
Rv2216	2483626	2484531	1111.302	Rv2307B	2579504	2579935	13.483
Rv2217	2484584	2485276	333.166	Rv2308	2580419	2581135	24.724
Rv2219	2486235	2486987	258.956	Rv2309A	2583045	2583332	10.669
Rv2220	2487615	2489051	263.802	Rv2310	2583435	2583779	6.173
Rv2222c	2492402	2493742	377.34	Rv2311	2583884	2584408	30.2
Rv2225	2497742	2498587	336.745	Rv2316	2588838	2589710	12.175
Rv2238c	2510715	2511176	291.251	Rv2317	2589697	2590521	7.025
Rv2239c	2511176	2511652	1025.052	Rv2318	2590518	2591840	11.04
Rv2240c	2511690	2512280	4941.607	Rv2319c	2591848	2592726	17.336
Rv2241	2512539	2515244	3173.474	Rv2320c	2592723	2594153	14.989
Rv2243	2516787	2517695	1222.963	Rv2323c	2595361	2596269	24.567
Rv2244	2517771	2518118	1089.419	Rv2324	2596334	2596780	48.762
Rv2245	2518115	2519365	705.844	Rv2330c	2603695	2604222	27.95
Rv2246	2519396	2520712	321.259	Rv2331A	2604740	2605078	55.68
Rv2247	2520743	2522164	295.509	Rv2332	2605108	2606754	40.534
Rv2256c	2529341	2529874	397.836	Rv2333c	2606708	2608321	38.106
Rv2258c	2530836	2531897	246.511	Rv2336	2610837	2611805	44.864
Rv2259	2532245	2533330	312.939	Rv2337c	2611869	2612987	55.958
Rv2271	2545332	2545631	591.475	Rv2339	2614693	2617581	53.247
Rv2280	2551560	2552939	327.711	Rv2341	2619597	2620016	10.135
Rv2299c	2570059	2572002	290.829	Rv2352c	2632923	2634098	53.224
Rv2302	2573813	2574055	276.379	Rv2353c	2634528	2635592	0.168
Rv2329c	2601914	2603461	2385.673	Rv2370c	2650245	2651558	19.1
Rv2334	2608796	2609728	1935.34	Rv2378c	2656408	2657703	28.099
Rv2345	2623821	2625803	637.9	Rv2379c	2657700	2662085	42.587
Rv2346c	2625888	2626172	292.466	Rv2380c	2662067	2667115	31.472
Rv2347c	2626223	2626519	374.09	Rv2381c	2667255	2670269	19.533
Rv2348c	2626654	2626980	287.725	Rv2382c	2670269	2671603	32.588
Rv2372c	2652037	2652825	687.739	Rv2383c	2671593	2675837	26.306
Rv2373c	2652839	2653987	774.826	Rv2384	2675936	2677633	11.222
Rv2374c	2654062	2655093	433.026	Rv2385	2677729	2678649	19.703
Rv2375	2655265	2655582	334.887	Rv2386c	2678653	2680005	11.556
Rv2376c	2655609	2656115	10118.533	Rv2398c	2694981	2695799	42.598
Rv2391	2684679	2686370	2353.552	Rv2401A	2698042	2698245	43.491
Rv2392	2686367	2687131	657.125	Rv2407	2704697	2705518	17.995
RVnc0024	2692224	2692439	250.312	Rv2415c	2712891	2713784	40.222
Rv2395A	2692224	2692439	1413.798	Rv2416c	2714124	2715332	50.79
Rv2395B	2692551	2692715	494.696	Rv2423	2719597	2720643	26.133
Rv2396	2692799	2693884	498.1	Rv2424c	2720776	2721777	15.764
Rv2397c	2693909	2694964	452.65	Rv2432c	2728437	2728847	56.314
Rv2412	2710075	2710335	579.785	Rv2433c	2728844	2729134	36.147
Rv2428	2726193	2726780	1268.171	Rv2434c	2729115	2730560	27.688
Rv2429	2726806	2727339	775.102	Rv2435c	2730557	2732749	26.032
Rv2430c	2727336	2727920	433.914	Rv2436	2733230	2734144	40.08
Rv2431c	2727967	2728266	343.172	Rv2437	2734376	2734795	57.878
Rv2441c	2739772	2740032	586.265	Rv2452c	2752848	2752994	32.455
Rv2442c	2740047	2740361	349.205	Rv2458	2759779	2760687	32.764
Rv2444c	2742123	2744984	401.227	Rv2478c	2784123	2784608	36.868
Rv2445c	2745314	2745724	954.221	Rv2485c	2792723	2793988	37.334
Rv2450c	2751662	2752180	955.131	Rv2487c	2795301	2797385	16.485
Rv2454c	2753625	2754746	858.701	Rv2488c	2797467	2800880	21.244
Rv2455c	2754743	2756704	536.476	Rv2489c	2800846	2801145	23.921
Rv2457c	2758208	2759488	332.814	Rv2490c	2801254	2806236	17.735
Rv2460c	2762531	2763175	942.971	Rv2491	2806665	2807288	8.144
Rv2461c	2763172	2763774	770.396	Rv2492	2807278	2808030	11.086
Rv2462c	2763891	2765291	254.686	Rv2501c	2814916	2816880	50.882
Rv2463	2765655	2766839	422.472	Rv2505c	2819953	2821596	21.549

Table S3. Highly expressed and less expressed genes in the mid-exponential growth phase

Rv2468c	2771644	2772147	256.85		Rv2506	2821712	2822359	50.541
Rv2468A	2772098	2772331	256.306		Rv2510c	2825488	2827089	58.775
Rv2476c	2777388	2782262	316.794		Rv2514c	2830877	2831338	20.266
Rv2477c	2782366	2784042	421.313		Rv2515c	2831344	2832591	15.486
Rv2512c	2828556	2829803	263.725		Rv2516c	2832710	2833513	36.519
Rv2521	2837684	2838157	320.695		Rv2529	2852875	2854266	40.942
Rv2523c	2839538	2839930	366.94		Rv2544	2867124	2867786	7.394
Rv2524c	2840123	2849332	395.691		Rv2545	2867783	2868061	27.738
Rv2525c	2849852	2850574	254.679		Rv2546	2868154	2868567	9.796
Rv2526	2851091	2851318	355.229		Rv2566	2886373	2889795	54.82
Rv2533c	2858254	2858724	266.106		Rv2569c	2893464	2894408	51.853
Rv2534c	2858727	2859290	439.752		Rv2570	2894512	2894901	46.254
Rv2535c	2859300	2860418	270.96		Rv2573	2898043	2898783	24.163
Rv2536	2860452	2861144	394.638		Rv2577	2900918	2902507	14.179
Rv2539c	2862673	2863203	404.001		Rv2578c	2902509	2903531	15.003
Rv2540c	2863207	2864412	444.898		Rv2598	2926986	2927480	26.649
Rv2541	2864427	2864834	316.796		Rv2607	2934198	2934872	36.876
Rv2554c	2873258	2873770	311.846		Rv2620c	2947462	2947887	56.798
Rv2555c	2873771	2876485	861.869		Rv2622	2948636	2949457	35.092
Rv2556c	2876576	2876965	246.259		Rv2625c	2951322	2952503	54.014
Rv2557	2877072	2877746	476.936		Rv2630	2956893	2957432	47.937
Rv2558	2877831	2878541	403.062		Rv2631	2957572	2958870	58.03
Rv2576c	2900226	2900690	352.982		Rv2634c	2960105	2962441	40.425
Rv2584c	2910229	2910900	515.494		Rv2635	2962470	2962712	19.58
Rv2586c	2912683	2914011	299.415		Rv2636	2962713	2963390	23.837
Rv2587c	2914015	2915736	386.436		Rv2639c	2965026	2965358	54.262
Rv2588c	2915846	2916193	295.036		Rv2640c	2965478	2965837	48.811
Rv2595	2925492	2925737	470.204		Rv2643	2966910	2968406	31.301
Rv2600	2927990	2928391	268.188		Rv2644c	2968533	2968850	29.12
Rv2602	2930344	2930784	499.284		Rv2646	2970551	2971549	37.356
Rv2603c	2930805	2931560	290.289		Rv2647	2971659	2972027	31.887
Rv2605c	2932297	2933142	295.013		Rv2650c	2973795	2975234	39.515
Rv2606c	2933171	2934070	316.269		Rv2652c	2975928	2976554	22.208
Rv2633c	2959335	2959820	280.782		Rv2653c	2976586	2976909	10.311
Rv2656c	2978660	2979052	294.981		Rv2654c	2976989	2977234	9.488
Rv2657c	2979049	2979309	248.292		Rv2655c	2977231	2978658	56.277
Rv2665	2982699	2982980	542.023		Rv2658c	2979326	2979688	41.368
Rv2674	2990706	2991116	754.738		Rv2659c	2979691	2980818	34.171
Rv2694c	3011399	3011767	301.994		Rv2661c	2981187	2981576	25.052
Rv2696c	3012829	3013608	568.697		Rv2662	2981482	2981754	27.61
Rv2697c	3013683	3014147	253.428		Rv2664	2982097	2982351	59.134
Rv2698	3014173	3014658	571.646		Rv2667	2983896	2984654	11.942
Rv2699c	3014663	3014965	547.734		Rv2669	2985283	2985753	39.329
Rv2700	3015203	3015853	288.339		Rv2670c	2985731	2986840	30.191
Rv2703	3017835	3019421	574.371		Rv2685	3001983	3003269	56.417
Rv2706c	3020200	3020457	879.821		Rv2686c	3003280	3004038	29.546
Rv2707	3020573	3021547	679.224		Rv2687c	3004035	3004748	25.078
Rv2708c	3021548	3021796	293.051		Rv2689c	3005845	3007062	42.866
Rv2710	3022461	3023432	317.444		Rv2723	3034909	3036102	39.138
Rv2711	3023565	3024257	432.259		Rv2727c	3039825	3040769	48.491
Rv2715	3028098	3029123	418.816		Rv2735c	3047560	3048552	47.158
Rv2718c	3030413	3030877	338.439		Rv2754c	3067193	3067945	52.014
Rv2720	3031788	3032498	316.569		Rv2756c	3068461	3070083	45.819
Rv2721c	3032520	3034619	350.739		Rv2757c	3070170	3070586	46.26
Rv2725c	3037427	3038914	249.531		Rv2758c	3070583	3070849	44.282
Rv2726c	3038931	3039800	294.457		Rv2761c	3071546	3072640	58.432
Rv2732c	3044375	3044989	311.595		Rv2765	3074636	3075373	45.157
Rv2736c	3048562	3049086	642.294		Rv2767c	3076367	3076720	33.685
Rv2737c	3049052	3051424	562.813		Rv2768c	3076894	3078078	36.17
Rv2743c	3056420	3057232	547.574		Rv2769c	3078158	3078985	30.27
Rv2744c	3057251	3058063	290.183		Rv2779c	3086215	3086754	48.932
Rv2745c	3058193	3058531	464.344		Rv2787	3095111	3096874	32.062
Rv2748c	3059855	3062506	295.892		Rv2797c	3105619	3107307	44.835
Rv2750	3062816	3063634	496.074		Rv2804c	3112465	3113094	47.19
Rv2753c	3066222	3067124	741.707		Rv2805	3112867	3113271	58.755
Rv2778c	3085713	3086183	300.417		Rv2806	3113268	3113459	49.734
Rv2782c	3089045	3090361	1239.701		Rv2807	3113658	3114812	6.004
Rv2783c	3090339	3092597	720.7		Rv2811	3116139	3116747	4.448
Rv2784c	3092951	3093466	296.259		Rv2812	3116818	3118227	0.793
Rv2785c	3093479	3093748	853.867		Rv2813	3118224	3119036	5.946
Rv2791c	3100202	3101581	257.171		Rv2818c	3124996	3126144	23.325
Rv2792c	3101581	3102162	369.584		Rv2819c	3126240	3127367	39.843
Rv2816c	3123625	3123966	305.39		Rv2820c	3127364	3128272	37.859
Rv2817c	3123967	3124983	312.293		Rv2823c	3129344	3131773	21.909
Rv2828A	3136330	3136599	281.758		Rv2824c	3131770	3132714	28.413
Rv2829c	3136620	3137012	518.783		Rv2826c	3133709	3134593	34.766
Rv2830c	3137009	3137224	562.138		Rv2833c	3139174	3140484	14.008
Rv2837c	3143635	3144645	1260.786		Rv2834c	3140487	3141314	13.703
Rv2838c	3144620	3145171	403.558		Rv2835c	3141311	3142222	11.311
Rv2839c	3145171	3147873	438.614		Rv2848c	3156148	3157521	50.481

Table S3. Highly expressed and less expressed genes in the mid-exponential growth phase

Rv2840c	3147959	3148258	259.373	Rv2854	3164152	3165192	41.93
Rv2844	3150713	3151201	413.007	Rv2856	3166684	3167802	42.463
Rv2846c	3153039	3154631	250.703	Rv2862A	3174747	3174995	10.815
Rv2861c	3173160	3174017	307.78	Rv2863	3174992	3175372	47.996
Rv2868c	3179368	3180531	368.182	Rv2874	3184847	3186934	10.517
Rv2876	3187663	3187977	567.305	Rv2875	3187030	3187611	27.121
Rv2882c	3191644	3192201	922.048	Rv2886c	3195545	3196432	27.769
Rv2883c	3192373	3193158	1012.614	Rv2891	3200266	3201015	12.354
Rv2888c	3196864	3198285	275.773	Rv2892c	3200794	3202020	38.139
Rv2889c	3198292	3199107	261.858	Rv2893	3202420	3203397	53.693
Rv2890c	3199119	3199982	459.995	Rv2897c	3206431	3207942	10.695
Rv2894c	3203394	3204290	415.355	Rv2898c	3207942	3208328	53.214
Rv2895c	3204381	3205232	724.062	Rv2899c	3208576	3209406	49.684
Rv2901c	3211803	3212108	1156.624	Rv2912c	3219274	3219861	53.727
Rv2902c	3212162	3212956	424.459	Rv2913c	3219863	3221698	41.998
Rv2903c	3212970	3213854	725.951	Rv2914c	3221767	3223524	29.933
Rv2904c	3213912	3214253	930.872	Rv2917	3226363	3228243	37.236
Rv2905	3214628	3215572	276.761	Rv2918c	3228254	3230680	30.883
Rv2908c	3216905	3217147	265.799	Rv2920c	3231073	3232506	15.987
Rv2909c	3217155	3217643	481.035	Rv2924c	3238601	3239470	38.581
Rv2916c	3224708	3226285	882.159	Rv2932	3251072	3255688	9.505
Rv2925c	3239829	3240551	507.95	Rv2933	3255685	3262251	19.492
Rv2926c	3240548	3241171	620.558	Rv2934	3262248	3267731	30.052
Rv2927c	3241222	3241959	654.856	Rv2943	3288464	3289705	48.557
Rv2930	3243697	3245448	282.886	Rv2943A	3289705	3290235	49.763
Rv2940c	3276380	3282715	386.156	Rv2957	3309470	3310297	58.837
Rv2941	3283335	3285077	300.317	Rv2961	3313283	3313672	25.339
Rv2942	3285070	3287832	274.222	Rv2964	3316529	3317461	34.438
Rv2945c	3290624	3291325	641.02	Rv2973c	3327733	3329946	23.826
Rv2946c	3291503	3296353	582.201	Rv2974c	3329949	3331361	49.309
Rv2948c	3297837	3299954	401.892	Rv2983	3339118	3339762	41.093
Rv2949c	3299971	3300570	355.031	Rv2997	3355099	3356541	44.609
Rv2950c	3300596	3302455	317.911	Rv2998	3356815	3357276	59.345
Rv2951c	3303103	3304248	386.308	Rv2999	3357602	3358567	50.632
Rv2952	3304441	3305253	509.03	Rv3017c	3376490	3376852	49.704
Rv2954c	3306666	3307391	503.207	Rv3018c	3376939	3378243	12.341
Rv2959c	3312101	3312838	267.924	Rv3019c	3378711	3379001	18.883
Rv2960c	3312953	3313201	522.168	Rv3020c	3379036	3379329	17.622
Rv2969c	3323709	3324476	4739.629	Rv3022A	3380679	3380993	3.061
Rv2971	3326101	3326949	324.905	Rv3026c	3385163	3386077	29.81
Rv2985	3342165	3343118	268.183	Rv3032A	3392812	3393201	59.641
Rv2986c	3343176	3343820	462.913	Rv3033	3393380	3393928	40.583
Rv2989	3346147	3346848	360.592	Rv3037c	3397214	3398290	56.501
Rv2993c	3350274	3350993	539.286	Rv3047c	3408022	3408306	55.091
Rv2995c	3352458	3353468	271.218	Rv3054c	3415435	3415989	18.881
Rv2996c	3353483	3355069	321.238	Rv3055	3416081	3416695	41.572
Rv3001c	3359585	3360586	268.875	Rv3056	3416705	3417745	39.265
Rv3002c	3360624	3361130	546.498	Rv3060c	3421741	3423213	56.242
Rv3003c	3361130	3362986	1981.072	Rv3061c	3423262	3425427	25.521
Rv3004	3363348	3363686	1583.218	Rv3063	3427243	3429519	34.037
Rv3005c	3363693	3364532	498.765	Rv3064c	3429825	3430250	32.712
Rv3028c	3387075	3388031	397.464	Rv3070	3434087	3434467	57.29
Rv3029c	3388070	3388870	360.726	Rv3071	3434464	3435573	31.824
Rv3043c	3403200	3404921	437.086	Rv3072c	3435798	3436322	42.953
Rv3045	3406285	3407325	714.958	Rv3073c	3436329	3436685	36.038
Rv3046c	3407314	3407688	1133.284	Rv3079c	3441770	3442597	28.757
Rv3050c	3411217	3411957	2103.255	Rv3080c	3442656	3445988	19.95
Rv3051c	3412085	3414166	489.086	Rv3081	3446040	3447278	39.936
Rv3052c	3414232	3414684	277.227	Rv3082c	3447404	3448426	18.415
Rv3053c	3414719	3414958	488.231	Rv3084	3449997	3450923	51.63
Rv3075c	3438050	3438973	423.268	Rv3085	3450920	3451750	46.399
Rv3092c	3460814	3461734	288.648	Rv3087	3452925	3454343	44.781
Rv3093c	3461760	3462764	340.393	Rv3088	3454340	3455764	33.134
Rv3094c	3462761	3463891	700.065	Rv3089	3455761	3457272	38.105
Rv3099c	3468413	3469264	414.834	Rv3090	3458211	3459098	44.828
Rv3117	3483974	3484807	286.713	Rv3097c	3465778	3467091	25.824
Rv3118	3484809	3485111	382.187	Rv3106	3474007	3475377	44.246
Rv3119	3485132	3485575	360.463	Rv3107c	3475378	3476961	53.061
Rv3122	3488089	3488559	264.72	Rv3108	3477060	3477500	37.438
Rv3123	3488569	3489063	246.239	Rv3109	3477649	3478728	7.002
Rv3130c	3494975	3496366	386.93	Rv3110	3478779	3479174	17.485
Rv3131	3496551	3497549	709.285	Rv3111	3479171	3479683	9.429
Rv3134c	3499943	3500749	2460.175	Rv3112	3479700	3479951	13.624
Rv3135	3501334	3501732	2136.557	Rv3113	3480074	3480742	6.759
Rv3136	3501794	3502936	489.182	Rv3114	3480759	3481289	15.393
Rv3136A	3502945	3503277	361.879	Rv3124	3489506	3490375	20.036
Rv3137	3503393	3504175	453.757	Rv3125c	3490476	3491651	35.591
Rv3139	3505363	3506769	262.835	Rv3126c	3491808	3492122	20.216
Rv3140	3506790	3507995	271.447	Rv3159c	3527391	3529163	36.693
Rv3142c	3509118	3509546	282.401	Rv3162c	3531208	3531645	15.037

Table S3. Highly expressed and less expressed genes in the mid-exponential growth phase

Rv3143	3509654	3510055	255.425	Rv3163c	3531642	3532913	12.767
Rv3145	3511682	3512068	282.349	Rv3164c	3532943	3533905	24.21
Rv3146	3512077	3512631	280.996	Rv3165c	3533913	3534395	17.899
Rv3147	3512628	3513338	451.447	Rv3166c	3534392	3535351	26.663
Rv3148	3513338	3514660	316.344	Rv3167c	3535431	3536057	11.997
Rv3149	3514657	3515415	378.326	Rv3168	3536102	3537238	40.039
Rv3150	3515412	3516749	387.608	Rv3170	3538505	3539851	57.289
Rv3151	3516746	3519166	264.583	Rv3174	3542138	3542845	6.354
Rv3153	3520507	3521142	259.842	Rv3175	3542860	3544347	58.907
Rv3154	3521139	3521927	328.66	Rv3176c	3544344	3545300	6.991
Rv3155	3521924	3522223	291.951	Rv3177	3545447	3546307	11.409
Rv3156	3522234	3524135	273.077	Rv3178	3546438	3546797	7.845
Rv3157	3524132	3525793	329.475	Rv3179	3547618	3548907	37.454
Rv3158	3525790	3527385	706.468	Rv3180c	3549254	3549688	48.668
Rv3171c	3539846	3540745	305.644	Rv3181c	3549691	3550143	53.158
Rv3190A	3556855	3557064	252.991	Rv3182	3550374	3550718	40.089
Rv3197	3567024	3568367	395.957	Rv3183	3550715	3551044	20.653
Rv3198A	3571335	3571589	366.121	Rv3189	3554642	3555262	36.771
Rv3205c	3581627	3582505	344.162	Rv3192	3559563	3560024	42.666
Rv3207c	3583801	3584658	627.148	Rv3195	3564364	3565782	15.431
Rv3208	3585004	3585690	646.137	Rv3201c	3573731	3577036	24.799
Rv3208A	3585677	3585949	363.979	Rv3202c	3577033	3580200	42.155
Rv3211	3587798	3589381	713.505	Rv3229c	3605751	3607034	17.299
Rv3212	3589394	3590617	249.804	Rv3230c	3607112	3608254	26.168
Rv3213c	3590692	3591492	4578.207	Rv3236c	3611959	3613116	43.698
Rv3217c	3593804	3594235	280.303	Rv3239c	3614457	3617603	34.912
Rv3219	3595713	3595967	290.669	Rv3242c	3621570	3622211	35.113
Rv3220c	3596029	3597534	597.766	Rv3253c	3632097	3633584	50.144
Rv3221c	3597551	3597766	395.871	Rv3263	3643177	3644838	38.607
Rv3221A	3598051	3598356	761.126	Rv3279c	3661212	3662012	55.99
Rv3222c	3598353	3598904	268.631	Rv3289c	3670034	3670411	51.461
Rv3224	3599851	3600699	421.89	Rv3290c	3670445	3671794	33.651
Rv3241c	3620610	3621254	1287.734	Rv3294c	3675186	3675995	20.528
Rv3245c	3624910	3626613	745.48	Rv3296	3676775	3681316	48.911
Rv3246c	3626663	3627349	468.847	Rv3298c	3682110	3683024	57.516
Rv3248c	3628160	3629647	690.799	Rv3315c	3703464	3703865	46.876
Rv3249c	3629752	3630387	432.435	Rv3322c	3708438	3709052	39.06
Rv3250c	3630384	3630566	327.748	Rv3323c	3709049	3709714	55.054
Rv3251c	3630571	3630738	812.062	Rv3324c	3709715	3710248	25.035
Rv3257c	3637312	3638709	498.554	Rv3327	3711749	3713461	24.362
Rv3258c	3638811	3639302	1485.564	Rv3335c	3721731	3722600	59.209
Rv3259	3639425	3639844	344.258	Rv3343c	3729364	3736935	20.665
Rv3260c	3639872	3640141	445.888	Rv3345c	3738158	3742774	34.476
Rv3264c	3644898	3645977	377.505	Rv3346c	3743198	3743455	30.874
Rv3267	3647885	3649381	1010.019	Rv3347c	3743711	3753184	35.066
Rv3268	3649420	3650109	411.776	Rv3348	3753765	3754256	47.161
Rv3269	3650234	3650515	425.643	Rv3349c	3754293	3755033	28.271
Rv3270	3650526	3652682	322.631	Rv3350c	3755952	3767102	24.62
Rv3271c	3652679	3653347	562.814	Rv3351c	3767346	3768140	6.728
Rv3273	3654637	3656931	374.633	Rv3352c	3768222	3768593	11.868
Rv3274c	3656920	3658089	468.788	Rv3353c	3768736	3768996	16.849
Rv3280	3662062	3663708	457.11	Rv3357	3770773	3771048	53.968
Rv3281	3663689	3664222	421.405	Rv3358	3771045	3771302	48.181
Rv3283	3664928	3665821	777.64	Rv3359	3771344	3772534	26.465
Rv3284	3665818	3666249	387.465	Rv3361c	3773016	3773567	59.062
Rv3285	3666357	3668159	295.196	Rv3366	3777737	3778201	37.091
Rv3295	3676066	3676731	311.623	Rv3370c	3781501	3784740	34.579
Rv3307	3694054	3694860	268.155	Rv3371	3784932	3786272	53.209
Rv3312A	3700705	3701016	454.15	Rv3375	3788621	3790048	34.725
Rv3331	3717090	3718598	292.277	Rv3378c	3792358	3793248	50.83
Rv3332	3718595	3719746	422.495	Rv3379c	3793257	3794867	11.301
Rv3376	3790156	3790809	445.383	Rv3382c	3796448	3797437	41.109
Rv3385c	3799635	3799943	3854.793	Rv3383c	3797437	3798489	30.998
Rv3389c	3803919	3804791	1717.715	Rv3386	3800092	3800796	32.225
Rv3407	3826252	3826551	747.876	Rv3387	3800786	3801463	35.194
Rv3408	3826548	3826958	273.222	Rv3391	3805621	3807573	39.332
Rv3409c	3826991	3828727	700.137	Rv3392c	3807574	3808437	56.357
Rv3411c	3829930	3831519	427.606	Rv3393	3808461	3809387	47.406
Rv3412	3831726	3832136	346.759	Rv3394c	3809442	3811025	34.903
Rv3413c	3832146	3833045	1148.621	Rv3395c	3811022	3811636	42.809
Rv3414c	3833038	3833676	9923.7	Rv3397c	3814090	3814998	23.114
Rv3417c	3835272	3836891	276.995	Rv3398c	3815027	3816106	20.425
Rv3418c	3836986	3837288	310.79	Rv3399	3816129	3817175	54.254
Rv3420c	3838586	3839062	515.489	Rv3402c	3820653	3821891	24.843
Rv3429	3847165	3847701	1394.615	Rv3403c	3822262	3823863	43.669
Rv3441c	3860024	3861370	1681.888	Rv3406	3825330	3826217	22.427
Rv3442c	3861495	3861950	430.246	Rv3415c	3833694	3834521	28.73
Rv3443c	3861947	3862390	2120.393	Rv3427c	3843885	3844640	43.281
Rv3455c	3876052	3876822	1276.031	Rv3428c	3844738	3845970	3.719
Rv3456c	3876890	3877432	1680.698	Rv3432c	3850372	3851754	49.991

Table S3. Highly expressed and less expressed genes in the mid-exponential growth phase

Rv3457c	3877464	3878507	2117.067		Rv3434c	3853215	3853928	40.502
Rv3458c	3878659	3879264	1907.978		Rv3444c	3862624	3862926	36.487
Rv3459c	3879273	3879692	3071.408		Rv3445c	3862947	3863264	28.062
Rv3460c	3879696	3880070	2618.931		Rv3446c	3863317	3864531	7.475
Rv3461c	3880286	3880399	272.055		Rv3447c	3864528	3868238	4.663
Rv3462c	3880432	3880653	3284.339		Rv3448	3868352	3869755	6.818
Rv3464	3881837	3882832	3355.288		Rv3449	3869752	3871119	21.109
Rv3477	3894093	3894389	476.593		Rv3450c	3871084	3872496	34.049
Rv3478	3894426	3895607	1180.387		Rv3466	3883525	3884193	47.011
Rv3487c	3906174	3907007	287.649		Rv3467	3883964	3884917	15.761
Rv3489	3908072	3908236	380.452		Rv3468c	3884975	3886069	14.731
Rv3490	3908236	3909738	358.499		Rv3469c	3886073	3887083	8.387
Rv3508	3931005	3936710	396.623		Rv3470c	3887144	3888802	13.359
Rv3514	3945794	3950263	282.958		Rv3471c	3888808	3889341	8.387
Rv3519	3955550	3956260	380.902		Rv3472	3889362	3889868	49.473
Rv3520c	3956325	3957368	283.754		Rv3473c	3889948	3890733	9.225
Rv3524	3960755	3961786	557.333		Rv3476c	3892371	3893720	39.102
Rv3579c	4021425	4022393	979.753		Rv3481c	3900493	3901182	53.948
Rv3581c	4023868	4024347	3297.989		Rv3500c	3919220	3920062	49.454
Rv3582c	4024344	4025039	504.574		Rv3501c	3920097	3920861	55.966
Rv3583c	4025056	4025544	1074.292		Rv3504	3922471	3923673	25.81
Rv3584	4025830	4026378	1039.012		Rv3506	3924890	3926398	12.02
Rv3587c	4028968	4029762	870.335		Rv3513c	3945092	3945748	21.091
Rv3592	4034057	4034374	1353.569		Rv3515c	3950824	3952470	39.135
Rv3596c	4038158	4040704	262.249		Rv3516	3952544	3953335	43.459
Rv3597c	4040981	4041319	391.354		Rv3517	3953431	3954270	33.513
Rv3610c	4050601	4052883	545.223		Rv3522	3958448	3959512	40.753
Rv3613c	4053881	4054042	1120.29		Rv3527	3963605	3964054	50.825
Rv3614c	4054142	4054696	647.975		Rv3529c	3965884	3967038	9.374
Rv3615c	4054812	4055123	246.153		Rv3530c	3967038	3967820	12.776
Rv3616c	4055197	4056375	382.214		Rv3531c	3967817	3968944	49.442
Rv3628	4067423	4067911	639.601		Rv3532	3969343	3970563	20.904
Rv3633	4071791	4072666	271.698		Rv3533c	3970705	3972453	15.843
Rv3645	4082807	4084456	291.578		Rv3534c	3972552	3973592	58.822
Rv3646c	4084453	4087257	486.621		Rv3535c	3973589	3974500	57.043
Rv3647c	4087610	4088188	9442.656		Rv3537	3975369	3977060	36.957
Rv3648c	4088328	4088531	263.295		Rv3538	3977062	3977922	19.856
Rv3669	4110827	4111345	248.64		Rv3539	3978059	3979498	29.512
Rv3672c	4113521	4114342	351.957		Rv3540c	3979499	3980659	29.288
Rv3673c	4114474	4115157	389.322		Rv3541c	3980659	3981048	23.098
Rv3676	4116478	4117152	266.075		Rv3542c	3981045	3981980	24.383
Rv3677c	4117258	4118052	828.418		Rv3543c	3981977	3983140	35.42
Rv3678c	4118059	4118514	772.018		Rv3544c	3983125	3984144	50.559
Rv3678A	4118530	4118691	467.056		Rv3548c	3987382	3988296	58.91
Rv3679	4118776	4119798	450.395		Rv3549c	3988319	3989098	34.058
Rv3680	4119795	4120955	281.007		Rv3550	3989153	3989896	32.91
Rv3682	4121916	4124348	298.396		Rv3551	3989896	3990774	32.076
Rv3683	4124417	4125376	619.774		Rv3552	3990771	3991523	27.82
Rv3686c	4128751	4129083	273.946		Rv3553	3991621	3992688	34.71
Rv3688c	4129893	4130357	268.724		Rv3557c	3997029	3997631	51.645
Rv3699	4142044	4142745	326.905		Rv3558	3997980	3999638	44.001
Rv3708c	4151180	4152217	267.013		Rv3559c	3999647	4000435	33.867
Rv3709c	4152218	4153483	380.377		Rv3560c	4000432	4001589	29.441
Rv3711c	4155740	4156729	502.188		Rv3561	4001637	4003160	18.081
Rv3715c	4159889	4160500	753.419		Rv3562	4003161	4004294	27.618
Rv3716c	4160512	4160913	283.228		Rv3563	4004291	4005250	30.578
Rv3717	4161048	4161773	321.159		Rv3564	4005247	4006203	19.101
Rv3722c	4166821	4168128	424.819		Rv3565	4006200	4007366	21.124
Rv3723	4168536	4169300	381.871		Rv3566c	4007331	4008182	56.443
Rv3725	4170214	4171143	276.87		Rv3567c	4008719	4009282	37.001
Rv3726	4171421	4172614	269.886		Rv3571	4012417	4013493	39.675
Rv3734c	4184526	4185890	486.299		Rv3573c	4014077	4016212	17.305
Rv3747	4196724	4197107	288.798		Rv3577	4019262	4020128	46.199
Rv3750c	4198205	4198597	258.963		Rv3578	4020142	4021383	54.806
Rv3760	4205538	4205840	1123.951		Rv3591c	4033269	4034042	31.922
Rv3763	4209047	4209526	427.055		Rv3617	4057733	4058701	43.502
Rv3769	4214615	4214887	663.211		Rv3618	4058698	4059885	35.513
Rv3774	4218849	4219673	380.344		Rv3621c	4060648	4061889	16.156
Rv3778c	4223699	4224895	332.057		Rv3622c	4061899	4062198	7.999
Rv3780	4226989	4227525	251.689		Rv3635	4073634	4075409	40.27
Rv3794	4243233	4246517	696.422		Rv3638	4076984	4077730	11.445
Rv3799c	4254380	4255948	683.75		Rv3639c	4077884	4078450	12.518
Rv3800c	4255945	4261146	805.729		Rv3640c	4078520	4079749	5.874
Rv3801c	4261153	4263066	258.145		Rv3641c	4079925	4080560	31.292
Rv3802c	4263355	4264365	851.93		Rv3643	4081160	4081351	15.915
Rv3803c	4264563	4265462	3133.916		Rv3649	4088781	4091096	17.64
Rv3804c	4265642	4266658	246.183		Rv3650	4091233	4091517	23.532
Rv3807c	4269840	4270337	435.646		Rv3653	4093940	4094527	41.199
Rv3808c	4270366	4272279	306.374		Rv3655c	4094923	4095300	56.738
Rv3810	4273739	4274593	1629.464		Rv3656c	4095324	4095530	47.74

Table S3. Highly expressed and less expressed genes in the mid-exponential growth phase

Rv3837c	4311009	4311707	255.277		Rv3657c	4095540	4096115	23.012
Rv3841	4314178	4314723	4014.271		Rv3659c	4096936	4097994	9.908
Rv3842c	4314738	4315562	1195.191		Rv3660c	4098096	4099148	22.075
Rv3843c	4315568	4316596	521.1		Rv3663c	4102032	4103678	46.087
Rv3846	4320704	4321327	679.146		Rv3664c	4103675	4104475	16.68
Rv3847	4321538	4322071	268.214		Rv3665c	4104531	4105457	21.217
Rv3849	4323499	4323897	408.442		Rv3698	4140493	4142022	12.703
Rv3850	4324015	4324671	314.995		Rv3702c	4144913	4145614	33.575
Rv3852	4325074	4325478	787.16		Rv3714c	4158931	4159821	49.298
Rv3853	4325495	4325968	353.602		Rv3727	4172955	4174763	55.717
Rv3857c	4329417	4329614	271.618		Rv3728	4174873	4178070	39.256
Rv3858c	4330039	4331505	297.67		Rv3729	4178285	4180615	34.908
Rv3859c	4331498	4336081	293.491		Rv3730c	4180680	4181720	42.532
Rv3864	4340270	4341478	328.977		Rv3731	4181758	4182834	38.512
Rv3865	4341566	4341877	899.15		Rv3740c	4190833	4192179	54.35
Rv3866	4341880	4342731	454.693		Rv3743c	4193391	4195373	10.792
Rv3867	4342770	4343321	603.725		Rv3745c	4195886	4196098	30.258
Rv3868	4343314	4345035	340.917		Rv3746c	4196171	4196506	25.087
Rv3870	4346481	4348724	260.432		Rv3768	4214070	4214429	45.512
Rv3872	4350745	4351044	564.984		Rv3784	4230256	4231236	35.602
Rv3873	4351075	4352181	518.139		Rv3787c	4233610	4234536	33.068
Rv3874	4352274	4352576	11240.569		Rv3796	4249878	4251005	50.513
Rv3875	4352609	4352896	11087.04		Rv3797	4251085	4252866	35.88
Rv3878	4356693	4357535	416.496		Rv3812	4276571	4278085	57.28
Rv3879c	4357593	4359782	392.08		Rv3817	4281647	4282402	53.169
Rv3880c	4360199	4360546	853.531		Rv3828c	4302786	4303397	37.02
Rv3881c	4360543	4361925	596.43		Rv3829c	4303398	4305008	22.601
Rv3882c	4362032	4363420	246.99		Rv3830c	4305056	4305685	14.107
Rv3883c	4363417	4364757	406.191		Rv3832c	4306236	4306811	51
Rv3890c	4373726	4374013	846.697		Rv3833	4306867	4307658	41.877
Rv3891c	4374049	4374372	725.064		Rv3839	4312765	4313541	33.844
Rv3893c	4375762	4375995	368.746		Rv3840	4313567	4313980	40.102
Rv3898c	4383653	4383985	350.415		Rv3844	4318775	4319266	54.49
Rv3908	4393449	4394195	293.232		Rv3845	4319281	4319640	43.644
Rv3914	4402732	4403082	515.737		Rv3848	4322326	4323234	37.17
Rv3915	4403192	4404412	297.655		Rv3860	4336777	4337949	42.109
Rv3917c	4405457	4406491	265.988		Rv3895c	4380453	4381940	55.014
Rv3920c	4408334	4408897	711.784		Rv3902c	4387365	4387895	32.936
Rv3921c	4408969	4410069	643.734		Rv3903c	4387892	4390432	17.828
Rv3922c	4410053	4410415	372.622		Rv3904c	4390437	4390709	50.537
Rv3923c	4410412	4410789	463.387		Rv3905c	4390720	4391031	42.403
Rv3924c	4410786	4410929	1152.883		Rv3912	4400870	4401634	26.739
