# Supplementary material for: Genome-wide identification of the context-dependent sRNA expression in Mycobacterium tuberculosis
Source: BMC Genomics. 2020 Feb 18;21:167. doi: 10.1186/s12864-020-6573-5 (PMC7029489; doi:10.1186/s12864-020-6573-5)
Supplement: Supplementary file 10 — Additional file 10 Table S4. Context-dependent expression of the sRNAs. (a) List of 119 sRNAs expressed in the mid-exponential growth phase. (b) Context-dependent expression of all the 430 sRNAs derived by our method. (c) Binary matrix representation of the context-dependent expression of sRNAs across 15 growth conditions. (d) Expression matrix of the identified sRNAs in all the 15 growth conditions. [file 12864_2020_6573_MOESM10_ESM.pdf]

Table S4a. Putative sRNAs expressed in the mid-exponential phase

**Table S4a. Putative sRNAs expressed in the mid-exponential phase**			
sRNA ID	Start	End	Location
ncRv10049	53320	53570	Intergenic
ncRv10052	58124	58174	5'UTR of Rv0053
ncRv10062	66720	66770	Intergenic
ncRv10070c	79122	79222	5'UTR of Rv0070c
ncRv10128	156497	156547	3'UTR
ncRv10166	196683	196833	Intergenic and 5'UTR of Rv0167
ncRv10243	293544	293769	Intergenic and 3'UTR
ncRv10439c	528415	528565	Intergenic
ncRv10443	532937	533087	Intergenic
ncRv10484c	573927	573977	5'UTR of Rv0484c
ncRv10537c	629832	629882	5'UTR of Rv0537c
ncRv10597c	697004	697054	Intergenic
ncRv10650	748146	748196	5'UTR of Rv0651
ncRv10658c	754510	754560	Intergenic
ncRv10666	759685	759735	5'UTR of Rv0667
ncRv10668	767446	767546	Intergenic
ncRv10680c	780667	780717	Intergenic
ncRv10681	781462	781512	5'UTR of Rv0682
ncRv10684	784616	784666	Intergenic
ncRv10699	800276	800426	5'UTR of Rv0700
ncRv10867c	965636	965736	Intergenic and 3'UTR
ncRv10871	968356	968406	Intergenic
ncRv10932c	1041099	1041149	5'UTR of Rv0932c
ncRv10950c	1061880	1061930	Intergenic and 5'UTR of Rv0951
ncRv11012	1131547	1131597	5'UTR of Rv1013
ncRv11051c	1175251	1175301	Intergenic
ncRv11071c	1196193	1196243	5'UTR of Rv1072
ncRv11075c	1200546	1200596	Intergenic
ncRv11080c	1205899	1205949	5'UTR of Rv1080c
ncRv11094	1222837	1222962	Intergenic and 5'UTR of Rv1095
ncRv11133c	1261497	1261622	5'UTR of Rv1133c
ncRv11147	1275851	1275976	Intergenic
ncRv11156	1282919	1282969	Intergenic
ncRv11160	1287121	1287246	Intergenic and 5'UTR of Rv1161
ncRv11172c	1302732	1302832	Intergenic and 5'UTR of Rv1172c
ncRv11199c	1342706	1342856	5'UTR of Rv1199c
ncRv11296	1453048	1453098	5'UTR of Rv1297
ncRv11297	1455038	1455138	Intergenic and 5'UTR of Rv1298
ncRv11387	1563564	1563614	5'UTR of Rv1388
ncRv11435c	1612976	1613026	5'UTR of Rv1435c
ncRv11534	1735587	1735912	5'UTR of Rv1535
ncRv11535	1736263	1736313	Intergenic and 5'UTR of Rv1536
ncRv11629	1833480	1833530	Intergenic and 5'UTR of Rv1630
ncRv11640c	1852061	1852111	5'UTR of Rv1641
ncRv11733c	1960688	1960738	Intergenic
ncRv11765c	1999016	1999066	Intergenic
ncRv11804c	2047575	2047625	Intergenic and 5'UTR of Rv1806

Table S4a. Putative sRNAs expressed in the mid-exponential phase

ncRv11809	2052739	2052789	Intergenic
ncRv11809	2052814	2052914	Intergenic
ncRv11810	2053365	2053415	5'UTR of Rv1811
ncRv11813c	2056188	2056238	5'UTR of Rv1813c
ncRv11818c	2062725	2062775	Intergenic and 5'UTR of Rv1818c
ncRv11826	2072407	2072457	5'UTR of Rv1827
ncRv11979c	2223290	2223340	5'UTR of Rv1979c
ncRv12049c	2307543	2307593	5'UTR of Rv2049c
ncRv12080	2337895	2338020	3'UTR
ncRv12081c	2338531	2338581	5'UTR of Rv2081c
ncRv12146c	2405982	2406032	Intergenic and 5'UTR of Rv2146c
ncRv12161c	2423163	2423213	Intergenic and 5'UTR of Rv2161c
ncRv12219A	2487542	2487592	Intergenic and 5'UTR of Rv2220
ncRv12220	2489227	2489277	Intergenic and 3'UTR
ncRv12301	2573758	2573808	Intergenic
ncRv12374c	2655144	2655194	Intergenic
ncRv12390c	2684542	2684592	5'UTR of Rv2391
ncRv12395	2692151	2692201	Intergenic
ncRv12444c	2745110	2745160	5'UTR of Rv2444c
ncRv12461c	2763800	2763850	Intergenic and 5'UTR of Rv2461c
ncRv12560	2881278	2881328	Intergenic
ncRv12702	3017781	3017831	5'UTR of Rv2703
ncRv12710	3023458	3023508	Intergenic and 3'UTR
ncRv12744c	3058089	3058139	Intergenic and 5'UTR of Rv2744c
ncRv12783c	3092623	3092673	5'UTR of Rv2783c
ncRv12890c	3200033	3200183	Intergenic
ncRv12904c	3214304	3214554	Intergenic and 5'UTR of Rv2904c
ncRv12929	3243432	3243657	5'UTR of Rv2930
ncRv12939	3276293	3276343	Intergenic
ncRv12990c	3347745	3347870	5'UTR of Rv2990c
ncRv13000	3359372	3359547	Intergenic
ncRv13027c	3386970	3387020	Intergenic
ncRv13053c	3415034	3415084	5'UTR of Rv3053c
ncRv13134c	3501175	3501300	5'UTR of Rv3135
ncRv13197A	3568880	3568980	5'UTR of Rv3197A
ncRv13207c	3584734	3584784	5'UTR of Rv3207c
ncRv13210c	3587640	3587690	5'UTR of Rv3211
ncRv13221c	3597892	3597942	Intergenic
ncRv13241c	3621280	3621405	5'UTR of Rv3241c
ncRv13260c	3640242	3640367	Intergenic
ncRv13332	3719772	3719822	Intergenic
ncRv13332	3719872	3719922	Intergenic
ncRv13339c	3726045	3726095	5'UTR of Rv3340
ncRv13418c	3837339	3837514	Intergenic and 5'UTR of Rv3418c
ncRv13460c	3880096	3880146	5'UTR of Rv3460c
ncRv13462c	3880679	3880729	5'UTR of Rv3462c
ncRv13487c	3907058	3907108	Intergenic
ncRv13596c	4040805	4040855	Intergenic
ncRv13632	4071681	4071731	5'UTR of Rv3633

Table S4a. Putative sRNAs expressed in the mid-exponential phase

ncRv13647c	4088214	4088264	5'UTR of Rv3647c
ncRv13648c	4088657	4088707	Intergenic and 5'UTR of Rv3648c
ncRv13660c	4099274	4099399	5'UTR of Rv3660c
ncRv13661	4100686	4101161	Intergenic and 3'UTR
ncRv13680	4120981	4121031	Intergenic
ncRv13709c	4153659	4153709	5'UTR of Rv3710
ncRv13725	4171194	4171344	Intergenic and 5'UTR of Rv3726
ncRv13801c	4263092	4263217	5'UTR of Rv3801c
ncRv13802c	4264416	4264466	Intergenic and 5'UTR of Rv3802c
ncRv13803c	4265538	4265588	5'UTR of Rv3803c
ncRv13810	4274694	4274744	Intergenic
ncRv13822	4288111	4288161	Intergenic
ncRv13849	4323923	4323973	5'UTR of Rv3850
ncRv13859c	4336132	4336182	5'UTR of Rv3859c
ncRv13870	4348775	4348825	Intergenic
ncRv13916c	4405318	4405368	Intergenic
ncRv13919c	4408228	4408278	Intergenic
ncRv11005c	1123649	1123699	5'UTR of Rv1006
ncRv11737c	1965546	1965621	Intergenic and 5'UTR of Rv1738
ncRv12524c	2849358	2849433	5'UTR of Rv2524c
ncRv13520c	3957394	3957444	5'UTR of Rv3520c
ncRv12628	2955596	2955721	5'UTR of Rv2629
ncRv13646c	4087508	4087608	Intergenic

**Table S4b. sRNAs identified across 15 growth conditions**				
sRNA ID	Start	End	Location	Number of conditions expressed
ncRv10001	1950	2000	5'UTR of Rv0002	1
ncRv10009	13067	13117	Intergenic	4
ncRv10010c	13609	13659	Intergenic	1
ncRv10019c	23763	23838	5'UTR of Rv0019c	5
ncRv10049	53270	53570	Intergenic and 3'UTR	11
ncRv10052	58099	58174	Intergenic and 5'UTR of Rv0053	5
ncRv10053	58533	58583	Intergenic	1
ncRv10061c	65376	65526	5'UTR of Rv0061c	7
ncRv10062	66720	66770	Intergenic	1
ncRv10070cA	78997	79047	5'UTR of Rv0070c	6
ncRv10070cB	79097	79297	5'UTR of Rv0070c	6
ncRv10071A	80319	80369	Intergenic	4
ncRv10071B	80394	80444	Intergenic	4
ncRv10071C	80469	80519	Intergenic	4
ncRv10107cA	130567	130617	Intergenic	6
ncRv10107cB	130692	130842	Intergenic	6
ncRv10115A	139724	139774	Intergenic	2
ncRv10115B	139824	140024	Intergenic	2
ncRv10116c	141048	141098	Intergenic and 5'UTR of Rv0116c	1
ncRv10123	149325	149375	Intergenic	1
ncRv10128	156497	156572	Intergenic and 3'UTR	3
ncRv10129c	157651	157701	Intergenic and 5'UTR of Rv0129c	1
ncRv10135c	163275	163325	Intergenic	2
ncRv10165c	194841	194966	Intergenic and 5'UTR of Rv0166	2
ncRv10166	196683	196833	Intergenic and 5'UTR of Rv0167	7
ncRv10186A	218577	218652	Intergenic and 5'UTR of Rv0186A	3
ncRv10187	219418	219468	Intergenic	1
ncRv10190	222187	222237	Intergenic	1
ncRv10210	251674	251749	Intergenic and 5'UTR of Rv0211	2
ncRv10231	277840	277890	Intergenic and 5'UTR of Rv0232	1
ncRv10242c	292105	292155	Intergenic and 5'UTR of Rv0242c	1
ncRv10243	293519	293769	Intergenic and 3'UTR	12
ncRv10244c	295659	295734	Intergenic and 5'UTR of Rv0244c	3
ncRv10245	296544	296594	Intergenic	1
ncRv10251c	302678	302728	Intergenic	1
ncRv10279c	339299	339349	Intergenic and 5'UTR of Rv0280	1
ncRv10281	341932	342107	Intergenic and 5'UTR of Rv0282	8
ncRv10296c	361231	361331	Intergenic and 5'UTR of Rv0297	4
ncRv10297	363160	363210	Intergenic	1
ncRv10301	364520	364570	Intergenic and 5'UTR of Rv0302	1
ncRv10323c	391277	391327	Intergenic	1
ncRv10340	409299	409349	Intergenic and 5'UTR of Rv0341	1
ncRv10349	419709	419784	Intergenic and 5'UTR of Rv0350	3
ncRv10353A	424070	424170	Intergenic	2
ncRv10353B	424195	424245	Intergenic	2
ncRv10397	476335	476385	Intergenic	1
ncRv10414c	501044	501119	Intergenic and 5'UTR of Rv0415	3
ncRv10420c	506997	507047	Intergenic	1
ncRv10426c	515842	515917	5'UTR of Rv0426c	3
ncRv10429c	518672	518722	Intergenic and 5'UTR of Rv0430	1
ncRv10439c	528390	528565	Intergenic	14
ncRv10443	532937	533087	Intergenic	6
ncRv10466	557428	557503	Intergenic and 5'UTR of Rv0467	2
ncRv10468	559781	559831	Intergenic	3
ncRv10474	565669	565719	Intergenic	1
ncRv10475	566447	566497	Intergenic and 5'UTR of Rv0476	1
ncRv10484c	573852	573977	5'UTR of Rv0484c	7
ncRv10518	610934	610984	Intergenic	1
ncRv10537cA	629832	629882	5'UTR of Rv0537c	6
ncRv10537cB	629907	629957	Intergenic and 5'UTR of Rv0538	6
ncRv10563	654765	654865	Intergenic	4
ncRv10571c	664844	664994	Intergenic	3
ncRv10574c	668420	668470	Intergenic and 5'UTR of Rv0574c	2
ncRv10578c	675942	676067	Intergenic and 5'UTR of Rv0579	4
ncRv10597cA	696929	696979	Intergenic	6
ncRv10597cB	697004	697129	Intergenic	6
ncRv10609A	704158	704208	3'UTR	1
ncRv10650A	748071	748121	5'UTR of Rv0651	6
ncRv10650B	748146	748196	5'UTR of Rv0651	6
ncRv10655A	752622	752722	3'UTR	4
ncRv10655B	752797	752847	3'UTR	4
ncRv10658c	754510	754660	Intergenic	8

ncRv10666	759510	759785	Intergenic and 5'UTR of Rv0667	13
ncRv10668A	767346	767421	Intergenic	10
ncRv10668B	767446	767546	Intergenic	10
ncRv10668C	767571	767621	Intergenic	10
ncRv10680c	780592	780717	Intergenic	3
ncRv10681A	781362	781412	Intergenic and 5'UTR of Rv0682	10
ncRv10681B	781437	781537	5'UTR of Rv0682	10
ncRv10684	784616	784816	Intergenic and 5'UTR of Rv0685	10
ncRv10689cA	789662	789712	Intergenic	2
ncRv10689cB	789737	789787	Intergenic	2
ncRv10694	794551	794626	Intergenic	2
ncRv10699	800226	800476	5'UTR of Rv0700	14
ncRv10713A	811238	811288	5'UTR of Rv0714	2
ncRv10713B	811313	811363	5'UTR of Rv0714	2
ncRv10749A	841950	842000	Intergenic	1
ncRv10753c	846029	846104	Intergenic	2
ncRv10755c	850216	850266	5'UTR of Rv0755c	1
ncRv10756c	851542	851592	Intergenic	1
ncRv10810c	905113	905163	Intergenic and 5'UTR of Rv0810c	4
ncRv10813c	908069	908169	Intergenic and 5'UTR of Rv0813c	8
ncRv10815c	909344	909594	Intergenic and 5'UTR of Rv0815c	10
ncRv10817c	910868	910968	Intergenic and 5'UTR of Rv0818	4
ncRv10833	927636	927811	Intergenic and 3'UTR	6
ncRv10841	937936	938086	Intergenic	8
ncRv10847	944786	944936	Intergenic	4
ncRv10858c	954996	955071	Intergenic and 5'UTR of Rv0859	4
ncRv10867c	965561	965786	Intergenic and 3'UTR	12
ncRv10871	968331	968406	Intergenic	6
ncRv10884c	982705	982755	Intergenic and 5'UTR of Rv0885	1
ncRv10887cA	987097	987147	5'UTR of Rv0888	1
ncRv10887cB	987172	987222	5'UTR of Rv0888	1
ncRv10922	1029363	1029488	Intergenic	7
ncRv10932c	1041099	1041174	Intergenic and 5'UTR of Rv0932c	4
ncRv10950c	1061880	1061930	Intergenic and 5'UTR of Rv0951	1
ncRv10953c	1065063	1065113	Intergenic and 5'UTR of Rv0954	1
ncRv11005c	1123624	1123699	5'UTR of Rv1006	3
ncRv11012	1131547	1131597	5'UTR of Rv1013	2
ncRv11015c	1134644	1134694	5'UTR of Rv1015c	1
ncRv11026	1148179	1148229	Intergenic	1
ncRv11051c	1175251	1175301	Intergenic	1
ncRv11052A	1176362	1176412	Intergenic	4
ncRv11052B	1176462	1176512	Intergenic	4
ncRv11052C	1176587	1176637	5'UTR of Rv1054	4
ncRv11052D	1176662	1176762	5'UTR of Rv1054	4
ncRv11064c	1187374	1187424	5'UTR of Rv1065	4
ncRv11071c	1196143	1196268	Intergenic and 5'UTR of Rv1072	10
ncRv11072	1197141	1197216	5'UTR of Rv1073	8
ncRv11075c	1200546	1200596	Intergenic	1
ncRv11077	1203187	1203287	Intergenic and 5'UTR of Rv1078	3
ncRv11080c	1205824	1205974	5'UTR of Rv1080c	8
ncRv11094	1222812	1222962	Intergenic and 5'UTR of Rv1095	11
ncRv11101c	1230574	1230624	Intergenic	2
ncRv11133c	1261397	1261747	Intergenic and 5'UTR of Rv1133c	15
ncRv11147A	1275576	1275751	Intergenic	8
ncRv11147B	1275851	1275976	Intergenic	8
ncRv11147C	1276051	1276151	Intergenic	8
ncRv11156A	1282919	1282969	Intergenic	7
ncRv11156B	1282994	1283044	Intergenic	7
ncRv11160	1287096	1287321	Intergenic and 5'UTR of Rv1161	13
ncRv11169c	1300150	1300250	Intergenic and 5'UTR of Rv1169c	3
ncRv11172c	1302707	1302907	Intergenic and 5'UTR of Rv1172c	8
ncRv11173	1305527	1305652	Intergenic	9
ncRv11174c	1306052	1306152	Intergenic and 5'UTR of Rv1174c	3
ncRv11176c	1308893	1308993	Intergenic and 5'UTR of Rv1177	7
ncRv11179cA	1313400	1313500	Intergenic	3
ncRv11179cB	1313600	1313700	Intergenic and 5'UTR of Rv1180	3
ncRv11184c	1325662	1325762	Intergenic and 5'UTR of Rv1184c	2
ncRv11194c	1338864	1338989	Intergenic and 5'UTR of Rv1195	3
ncRv11199c	1342656	1342906	5'UTR of Rv1199c	12
ncRv11221	1365212	1365312	Intergenic and 5'UTR of Rv1222	5
ncRv11248c	1393078	1393178	5'UTR of Rv1248c	2
ncRv11268c	1418482	1418532	Intergenic	1
ncRv11296	1453023	1453198	5'UTR of Rv1297	13
ncRv11297	1455038	1455138	Intergenic and 5'UTR of Rv1298	5

Table S4b. sRNAs identified across 15 growth conditions

ncRv11302	1459610	1459760	Intergenic and 5'UTR of Rv1303	3
ncRv11323	1487107	1487157	Intergenic	2
ncRv11327c	1494476	1494526	Intergenic and 5'UTR of Rv1327c	1
ncRv11351	1518711	1518761	Intergenic and 5'UTR of Rv1352	1
ncRv11368	1541856	1541906	3'UTR	1
ncRv11374c	1547606	1547806	5'UTR of Rv1375	8
ncRv11385	1561345	1561445	Intergenic	5
ncRv11387	1563414	1563639	Intergenic and 5'UTR of Rv1388	7
ncRv11388	1564292	1564392	Intergenic	7
ncRv11396c	1573908	1574108	Intergenic	5
ncRv11405c	1581995	1582045	5'UTR of Rv1405c	3
ncRv11435c	1612976	1613026	5'UTR of Rv1435c	1
ncRv11440	1618121	1618171	Intergenic	3
ncRv11478	1669195	1669245	5'UTR of Rv1479	1
ncRv11490A	1680680	1680780	Intergenic	2
ncRv11490B	1680955	1681005	Intergenic	2
ncRv11501	1692812	1692862	5'UTR of Rv1502	1
ncRv11510	1703019	1703069	Intergenic and 5'UTR of Rv1511	1
ncRv11534	1735487	1735937	Intergenic and 5'UTR of Rv1535	15
ncRv11535	1736238	1736488	Intergenic and 5'UTR of Rv1536	14
ncRv11536	1739695	1739745	5'UTR of Rv1537	1
ncRv11591A	1792261	1792311	Intergenic	1
ncRv11591B	1792336	1792386	Intergenic	1
ncRv11592c	1793766	1793966	Intergenic and 5'UTR of Rv1592c	4
ncRv11614A	1814628	1814678	Intergenic	4
ncRv11614B	1814753	1814803	Intergenic and 5'UTR of Rv1615	4
ncRv11629	1833480	1833530	Intergenic and 5'UTR of Rv1630	1
ncRv11631	1836262	1836312	3'UTR	1
ncRv11640c	1852061	1852236	5'UTR of Rv1641	12
ncRv11645c	1855705	1855755	Intergenic and 5'UTR of Rv1646	1
ncRv11698	1923715	1923815	Intergenic and 5'UTR of Rv1699	2
ncRv11702cA	1928951	1929001	Intergenic	2
ncRv11702cB	1929026	1929076	Intergenic	2
ncRv11706A	1934750	1934875	Intergenic and 5'UTR of Rv1707	4
ncRv11706cA	1934004	1934079	Intergenic	4
ncRv11706cB	1934104	1934279	Intergenic	4
ncRv11733c	1960663	1960763	Intergenic	7
ncRv11734c	1961142	1961192	3'UTR	2
ncRv11737cA	1965396	1965446	Intergenic and 5'UTR of Rv1737c	4
ncRv11737cB	1965546	1965621	Intergenic and 5'UTR of Rv1738	4
ncRv11759cA	1992878	1992928	Intergenic	2
ncRv11759cB	1993028	1993103	Intergenic	2
ncRv11765c	1999016	1999091	Intergenic	6
ncRv11778c	2012556	2012606	Intergenic	2
ncRv11781cA	2017577	2017627	Intergenic	2
ncRv11781cB	2017677	2017727	5'UTR of Rv1782	2
ncRv11790	2029778	2029878	5'UTR of Rv1791	2
ncRv11794	2031994	2032219	Intergenic and 5'UTR of Rv1795	12
ncRv11803c	2046968	2047018	Intergenic	1
ncRv11804c	2047550	2047675	Intergenic and 5'UTR of Rv1806	7
ncRv11806	2049623	2049673	Intergenic	2
ncRv11806B	2049623	2049798	Intergenic	2
ncRv11808	2051201	2051251	3'UTR	1
ncRv11809A	2052739	2052789	Intergenic	7
ncRv11809B	2052814	2052914	Intergenic	7
ncRv11810	2053340	2053440	5'UTR of Rv1811	4
ncRv11812c	2055410	2055635	Intergenic and 5'UTR of Rv1812c	5
ncRv11813c	2056163	2056338	5'UTR of Rv1813c	9
ncRv11814	2057449	2057524	Intergenic and 5'UTR of Rv1815	2
ncRv11818c	2062725	2062775	Intergenic and 5'UTR of Rv1818c	1
ncRv11826	2072407	2072582	5'UTR of Rv1827	13
ncRv11828	2073850	2073925	5'UTR of Rv1829	3
ncRv11829A	2074463	2074513	3'UTR	4
ncRv11829B	2074563	2074613	3'UTR	4
ncRv11829C	2074638	2074688	Intergenic	4
ncRv11829D	2074763	2074838	Intergenic and 5'UTR of Rv1830	4
ncRv11832	2078728	2078853	3'UTR	2
ncRv11846c	2096750	2096825	Intergenic	4
ncRv11856c	2104874	2104974	Intergenic and 5'UTR of Rv1856c	2
ncRv11875A	2124850	2124900	Intergenic	4
ncRv11875B	2125025	2125100	Intergenic	4
ncRv11875C	2125125	2125175	Intergenic	4
ncRv11886c	2136143	2136218	5'UTR of Rv1887	3
ncRv11903	2150844	2150944	5'UTR of Rv1904	4

ncRv11914c	2160354	2160404	Intergenic	1
ncRv11941	2194560	2194610	Intergenic	1
ncRv11976cA	2219527	2219577	Intergenic and 5'UTR of Rv1977	2
ncRv11976cB	2219602	2219752	5'UTR of Rv1977	2
ncRv11979c	2223215	2223340	5'UTR of Rv1979c	6
ncRv11980c	2224055	2224180	Intergenic and 5'UTR of Rv1980c	6
ncRv11984c	2228937	2228987	Intergenic	1
ncRv11985c	2229953	2230003	Intergenic and 5'UTR of Rv1986	1
ncRv11986A	2230636	2230686	3'UTR	2
ncRv11986B	2230711	2230761	Intergenic and 3'UTR	2
ncRv11987	2231605	2231655	5'UTR of Rv1988	2
ncRv11988	2232645	2232720	Intergenic	2
ncRv11990c	2233663	2233713	Intergenic	1
ncRv11996A	2239983	2240058	Intergenic and 3'UTR	2
ncRv11996B	2240083	2240133	Intergenic and 5'UTR of Rv1997	2
ncRv12031c	2278958	2279008	Intergenic and 5'UTR of Rv2031c	1
ncRv12049cA	2307543	2307593	5'UTR of Rv2049c	4
ncRv12049cB	2307743	2307793	5'UTR of Rv2050	4
ncRv12075c	2332955	2333005	Intergenic	1
ncRv12080	2337895	2338020	3'UTR	2
ncRv12081c	2338531	2338631	Intergenic and 5'UTR of Rv2081c	5
ncRv12108	2368843	2368943	3'UTR	2
ncRv12111c	2370818	2370868	Intergenic and 5'UTR of Rv2111c	2
ncRv12115c	2376316	2376366	Intergenic and 5'UTR of Rv2115c	1
ncRv12126c	2388523	2388598	Intergenic and 5'UTR of Rv2127	3
ncRv12145cA	2405424	2405524	Intergenic and 5'UTR of Rv2145c	3
ncRv12145cB	2405549	2405624	Intergenic	3
ncRv12146c	2405982	2406082	Intergenic and 5'UTR of Rv2146c	3
ncRv12161c	2423163	2423213	Intergenic and 5'UTR of Rv2161c	4
ncRv12169c	2431995	2432070	Intergenic and 5'UTR of Rv2169c	3
ncRv12190c	2453298	2453398	Intergenic and 5'UTR of Rv2190c	2
ncRv12198c	2463073	2463173	Intergenic and 5'UTR of Rv2198c	6
ncRv12200c	2464777	2464852	Intergenic and 5'UTR of Rv2200c	4
ncRv12202c	2468103	2468153	Intergenic and 5'UTR of Rv2203	3
ncRv12214c	2481902	2481952	5'UTR of Rv2215	1
ncRv12219A	2487542	2487592	Intergenic and 5'UTR of Rv2220	1
ncRv12220	2489077	2489277	Intergenic and 3'UTR	9
ncRv12225	2498688	2498738	Intergenic	1
ncRv12301	2573758	2573808	Intergenic	2
ncRv12307c	2579147	2579197	Intergenic	1
ncRv12327A	2600505	2600555	Intergenic	2
ncRv12327B	2600580	2600630	Intergenic	2
ncRv12329c	2603512	2603562	Intergenic	1
ncRv12333cA	2608547	2608597	5'UTR of Rv2334	5
ncRv12333cB	2608697	2608772	5'UTR of Rv2334	5
ncRv12357c	2641190	2641240	Intergenic	1
ncRv12359	2642068	2642118	Intergenic	1
ncRv12374c	2655144	2655244	Intergenic and 5'UTR of Rv2375	2
ncRv12386cA	2680506	2680556	5'UTR of Rv2387	5
ncRv12386cB	2680681	2680756	5'UTR of Rv2387	5
ncRv12390cA	2684417	2684517	Intergenic and 5'UTR of Rv2391	14
ncRv12390cB	2684542	2684667	5'UTR of Rv2391	14
ncRv12395	2692126	2692201	Intergenic	5
ncRv12395A	2692490	2692540	Intergenic	2
ncRv12411c	2710016	2710066	Intergenic and 5'UTR of Rv2412	1
ncRv12442c	2740387	2740437	5'UTR of Rv2442c	2
ncRv12444c	2745035	2745235	Intergenic and 5'UTR of Rv2444c	12
ncRv12455c	2756730	2756805	Intergenic and 5'UTR of Rv2455c	2
ncRv12457cA	2759514	2759589	Intergenic and 5'UTR of Rv2457c	3
ncRv12457cB	2759614	2759664	Intergenic	3
ncRv12459	2762406	2762506	Intergenic	4
ncRv12461c	2763800	2763875	Intergenic and 5'UTR of Rv2461c	4
ncRv12484c	2792545	2792595	Intergenic and 5'UTR of Rv2484c	1
ncRv12497cA	2812122	2812272	5'UTR of Rv2497c	7
ncRv12497cB	2812297	2812347	Intergenic	7
ncRv12512cA	2829854	2829904	5'UTR of Rv2513	2
ncRv12512cB	2830054	2830154	5'UTR of Rv2513	2
ncRv12519	2837289	2837339	Intergenic	1
ncRv12524cA	2849358	2849433	5'UTR of Rv2524c	5
ncRv12524cB	2849483	2849533	Intergenic and 5'UTR of Rv2524c	5
ncRv12542	2866367	2866417	Intergenic	5
ncRv12560	2881278	2881328	Intergenic	1
ncRv12589	2917810	2917860	5'UTR of Rv2590	1
ncRv12615cA	2945111	2945211	Intergenic	1

ncRv12615cB	2945261	2945311	Intergenic	1
ncRv12617c	2946363	2946413	Intergenic	1
ncRv12622	2949533	2949583	Intergenic and 5'UTR of Rv2623	1
ncRv12626cA	2953094	2953344	Intergenic	2
ncRv12626cB	2953369	2953419	Intergenic	2
ncRv12628	2955496	2955721	Intergenic and 5'UTR of Rv2629	10
ncRv12632c	2959216	2959316	5'UTR of Rv2632c	4
ncRv12637	2964293	2964393	Intergenic	2
ncRv12664	2982577	2982652	5'UTR of Rv2665	4
ncRv12688c	3005676	3005726	5'UTR of Rv2688c	2
ncRv12694c	3011793	3011843	Intergenic and 5'UTR of Rv2694c	1
ncRv12702	3017681	3017831	Intergenic and 5'UTR of Rv2703	7
ncRv12706c	3020483	3020533	Intergenic	1
ncRv12709	3022386	3022436	Intergenic and 5'UTR of Rv2710	2
ncRv12710	3023458	3023533	Intergenic and 3'UTR	5
ncRv12744c	3058089	3058189	Intergenic and 5'UTR of Rv2744c	5
ncRv12783cA	3092623	3092673	5'UTR of Rv2783c	3
ncRv12783cB	3092698	3092798	5'UTR of Rv2783c	3
ncRv12792c	3102188	3102263	5'UTR of Rv2792c	2
ncRv12808	3115329	3115379	5'UTR of Rv2809	2
ncRv12813A	3119837	3119887	Intergenic	1
ncRv12813B	3120287	3120337	Intergenic	1
ncRv12813C	3120362	3120412	Intergenic	1
ncRv12842c	3150002	3150102	Intergenic and 5'UTR of Rv2842c	4
ncRv12866	3178311	3178411	Intergenic	3
ncRv12890c	3200033	3200183	Intergenic	8
ncRv12898c	3208404	3208454	Intergenic	1
ncRv12904cA	3214304	3214379	5'UTR of Rv2904c	9
ncRv12904cB	3214479	3214604	Intergenic and 5'UTR of Rv2905	9
ncRv12909c	3217669	3217719	5'UTR of Rv2909c	2
ncRv12929	3243382	3243682	5'UTR of Rv2930	12
ncRv12939	3276243	3276368	Intergenic	8
ncRv12940c	3282741	3282866	Intergenic and 5'UTR of Rv2940c	5
ncRv12942	3288083	3288133	5'UTR of Rv2943	3
ncRv12950c	3302481	3302556	5'UTR of Rv2950c	5
ncRv12953	3306561	3306611	Intergenic	1
ncRv12958c	3312026	3312076	Intergenic	1
ncRv12970c	3325754	3325854	5'UTR of Rv2970c	1
ncRv12990c	3347745	3347920	Intergenic and 5'UTR of Rv2990c	11
ncRv13000	3359347	3359547	Intergenic	9
ncRv13003c	3363087	3363137	5'UTR of Rv3003c	2
ncRv13027c	3386970	3387070	Intergenic	4
ncRv13034c	3395122	3395172	5'UTR of Rv3035	1
ncRv13053c	3414984	3415359	Intergenic and 5'UTR of Rv3053c	14
ncRv13059	3421446	3421496	Intergenic	1
ncRv13061c	3425528	3425578	5'UTR of Rv3062	1
ncRv13124	3490401	3490451	Intergenic	1
ncRv13130c	3496467	3496542	Intergenic and 5'UTR of Rv3131	2
ncRv13134c	3501150	3501300	Intergenic and 5'UTR of Rv3135	9
ncRv13171c	3540796	3540871	Intergenic and 5'UTR of Rv3171c	4
ncRv13176c	3545326	3545376	Intergenic and 5'UTR of Rv3176c	1
ncRv13197AA	3568730	3568830	5'UTR of Rv3197A	4
ncRv13197AB	3568855	3568980	5'UTR of Rv3197A	4
ncRv13202cA	3580301	3580351	5'UTR of Rv3203	1
ncRv13202cB	3580476	3580576	5'UTR of Rv3203	1
ncRv13207c	3584684	3584784	5'UTR of Rv3207c	2
ncRv13208A	3586125	3586175	5'UTR of Rv3209	1
ncRv13210c	3587615	3587740	5'UTR of Rv3211	12
ncRv13218	3595534	3595709	Intergenic and 5'UTR of Rv3219	13
ncRv13221c	3597792	3598042	Intergenic and 5'UTR of Rv3221c	15
ncRv13223c	3599577	3599752	Intergenic and 5'UTR of Rv3223c	7
ncRv13241c	3621280	3621455	5'UTR of Rv3241c	11
ncRv13260c	3640167	3640417	Intergenic and 5'UTR of Rv3260c	7
ncRv13287c	3669439	3669514	Intergenic	3
ncRv13303c	3691014	3691064	Intergenic	1
ncRv13332A	3719772	3719822	Intergenic	1
ncRv13332B	3719872	3719922	Intergenic	1
ncRv13339c	3725970	3726095	Intergenic and 5'UTR of Rv3340	6
ncRv13401	3820453	3820553	Intergenic	2
ncRv13402cA	3821942	3821992	5'UTR of Rv3402c	2
ncRv13402cB	3822067	3822242	Intergenic	2
ncRv13411c	3831545	3831595	Intergenic and 5'UTR of Rv3411c	1
ncRv13415c	3834772	3834872	Intergenic and 5'UTR of Rv3416	3
ncRv13418c	3837314	3837489	Intergenic and 5'UTR of Rv3418c	13

ncRv13424c	3842102	3842152	Intergenic	3
ncRv13425	3842920	3842970	Intergenic	4
ncRv13443c	3862416	3862491	5'UTR of Rv3443c	4
ncRv13460c	3880096	3880271	5'UTR of Rv3460c	15
ncRv13462c	3880679	3880829	Intergenic and 5'UTR of Rv3462c	14
ncRv13476c	3893921	3894071	Intergenic and 5'UTR of Rv3477	6
ncRv13478	3895658	3895783	Intergenic	13
ncRv13483c	3902963	3903063	5'UTR of Rv3484	2
ncRv13487c	3907033	3907508	Intergenic	13
ncRv13490	3909764	3909839	Intergenic	3
ncRv13503c	3922282	3922332	5'UTR of Rv3503c	1
ncRv13520c	3957419	3957519	5'UTR of Rv3520c	7
ncRv13555c	3995750	3995800	Intergenic	1
ncRv13573c	4016288	4016413	Intergenic and 5'UTR of Rv3574	3
ncRv13595c	4038076	4038151	Intergenic	3
ncRv13596c	4040730	4040880	Intergenic and 5'UTR of Rv3596c	5
ncRv13614c	4054722	4054797	Intergenic and 5'UTR of Rv3614c	3
ncRv13616cA	4056401	4056451	Intergenic and 5'UTR of Rv3616c	3
ncRv13616cB	4056476	4056526	Intergenic	3
ncRv13632	4071681	4071781	5'UTR of Rv3633	5
ncRv13646c	4087283	4087383	Intergenic and 5'UTR of Rv3646c	5
ncRv13647c	4088214	4088314	Intergenic and 3'UTR	3
ncRv13648c	4088557	4088732	Intergenic and 5'UTR of Rv3648c	15
ncRv13660c	4099174	4099474	5'UTR of Rv3660c	13
ncRv13661	4100636	4101211	Intergenic and 3'UTR	15
ncRv13680	4120981	4121131	Intergenic	5
ncRv13681cA	4121630	4121680	5'UTR of Rv3681c	2
ncRv13681cB	4121830	4121880	Intergenic	2
ncRv13687c	4129792	4129842	Intergenic	5
ncRv13709c	4153584	4153709	Intergenic and 5'UTR of Rv3710	4
ncRv13723	4169326	4169376	Intergenic	1
ncRv13725	4171194	4171419	Intergenic and 5'UTR of Rv3726	12
ncRv13739c	4190618	4190718	Intergenic	5
ncRv13750c	4198623	4198698	Intergenic and 5'UTR of Rv3750c	3
ncRv13801c	4263092	4263292	Intergenic and 5'UTR of Rv3801c	12
ncRv13802c	4264391	4264516	Intergenic and 5'UTR of Rv3802c	4
ncRv13803c	4265538	4265638	5'UTR of Rv3803c	5
ncRv13804c	4266684	4266784	Intergenic and 5'UTR of Rv3804c	7
ncRv13810	4274644	4274769	Intergenic and 5'UTR of Rv3811	4
ncRv13822A	4287961	4288161	Intergenic	11
ncRv13822B	4288186	4288236	Intergenic	11
ncRv13823c	4291555	4291630	Intergenic	2
ncRv13836A	4310840	4310890	Intergenic	2
ncRv13836B	4310940	4310990	Intergenic	2
ncRv13846	4321353	4321528	Intergenic and 5'UTR of Rv3847	3
ncRv13847	4322147	4322272	Intergenic and 5'UTR of Rv3848	5
ncRv13848	4323435	4323485	Intergenic	1
ncRv13849	4323923	4323998	5'UTR of Rv3850	4
ncRv13855A	4328225	4328275	Intergenic	5
ncRv13855B	4328300	4328350	Intergenic	5
ncRv13859c	4336132	4336282	Intergenic and 5'UTR of Rv3859c	5
ncRv13870	4348775	4348825	Intergenic	5
ncRv13871	4350628	4350703	5'UTR of Rv3872	11
ncRv13875	4352922	4352997	Intergenic and 3'UTR	5
ncRv13879cA	4359833	4359883	5'UTR of Rv3879c	2
ncRv13879cB	4359908	4359958	5'UTR of Rv3879c	2
ncRv13879cC	4360008	4360058	Intergenic and 5'UTR of Rv3879c	2
ncRv13901c	4387215	4387265	Intergenic	2
ncRv13907c	4393274	4393349	5'UTR of Rv3908	4
ncRv13916cA	4405218	4405368	Intergenic and 5'UTR of Rv3916c	4
ncRv13916cB	4405393	4405443	Intergenic	4
ncRv13919c	4408228	4408303	Intergenic	3

Table S4c. Binary matrix representing the context-dependent expression of sRNAs																
sRNA ID	SRR8550302	SRR1917703	SRR1917706	SRR1917709	SRR1917712	SRR1917714	SRR3725585	SRR3725588	SRR3725591	SRR3725594	SRR3725597	SRR998928	SRR998931	SRR568038	SRR568040	Number of conditions expressed
ncRv10001	0	0	0	0	0	0	0	0	0	0	0	0	0	1	0	1
ncRv10009	0	1	1	1	1	0	0	0	0	0	0	0	0	0	0	4
ncRv10010c	0	0	0	0	0	1	0	0	0	0	0	0	0	0	0	1
ncRv10019c	0	1	0	1	1	0	1	0	0	0	0	0	0	1	0	5
ncRv10049	1	1	1	1	0	1	1	1	1	1	1	0	1	0	0	11
ncRv10052	1	0	0	1	1	1	0	0	0	0	0	1	0	0	0	5
ncRv10053	0	0	0	0	0	0	0	0	0	0	0	0	1	0	0	1
ncRv10061c	0	0	0	0	0	0	1	1	1	1	1	1	1	0	0	7
ncRv10062	1	0	0	0	0	0	0	0	0	0	0	0	0	0	0	1
ncRv10070cA	1	0	0	0	0	1	0	0	0	0	0	1	1	1	1	6
ncRv10070cB	0	0	0	0	0	1	0	0	0	0	0	1	1	1	1	6
ncRv10071A	0	0	0	0	0	0	0	1	1	1	1	0	0	0	0	4
ncRv10071B	0	0	0	0	0	0	0	1	1	1	1	0	0	0	0	4
ncRv10071C	0	0	0	0	0	0	0	1	1	1	1	0	0	0	0	4
ncRv10107cA	0	1	1	1	1	1	0	0	0	0	0	0	1	0	0	6
ncRv10107cB	0	1	1	1	1	1	0	0	0	0	0	0	1	0	0	6
ncRv10115A	0	0	0	0	0	1	0	0	0	0	0	1	0	0	0	2
ncRv10115B	0	0	0	0	0	1	0	0	0	0	0	1	0	0	0	2
ncRv10116c	0	0	0	0	0	0	0	0	0	0	0	0	0	0	1	1
ncRv10123	0	0	0	0	0	0	0	0	0	0	0	0	1	0	0	1
ncRv10128	1	1	0	0	1	0	0	0	0	0	0	0	0	0	0	3
ncRv10129c	0	0	0	0	0	0	0	0	0	0	0	0	0	1	0	1
ncRv10135c	0	0	1	1	0	0	0	0	0	0	0	0	0	0	0	2
ncRv10165c	0	1	0	0	0	1	0	0	0	0	0	0	0	0	0	2
ncRv10166	1	1	0	0	1	1	0	0	0	1	1	0	0	1	0	7
ncRv10186A	0	0	0	1	0	1	0	0	0	0	0	1	0	0	0	3
ncRv10187	0	0	0	0	0	0	0	0	0	0	0	0	1	0	0	1

Table S4c. Binary matrix

ncRv10190	0	0	0	0	0	0	1	0	0	0	0	0	0	0	0	1
ncRv10210	0	0	0	1	0	0	0	0	0	0	0	0	1	0	0	2
ncRv10231	0	0	0	1	0	0	0	0	0	0	0	0	0	0	0	1
ncRv10242c	0	0	0	1	0	0	0	0	0	0	0	0	0	0	0	1
ncRv10243	1	1	1	1	0	0	1	1	1	1	1	0	1	1	1	12
ncRv10244c	0	0	1	1	0	0	0	0	0	0	0	0	1	0	0	3
ncRv10245	0	0	0	1	0	0	0	0	0	0	0	0	0	0	0	1
ncRv10251c	0	0	0	0	0	0	0	0	0	0	0	0	1	0	0	1
ncRv10279c	0	0	0	1	0	0	0	0	0	0	0	0	0	0	0	1
ncRv10281	0	1	1	1	0	1	0	0	0	0	0	1	1	1	1	8
ncRv10296c	0	0	1	1	0	1	0	0	0	0	0	0	0	0	1	4
ncRv10297	0	0	0	0	0	0	0	0	0	0	0	0	0	0	1	1
ncRv10301	0	0	0	0	0	0	0	0	0	0	0	0	0	1	0	1
ncRv10323c	0	0	0	0	0	0	0	0	0	0	0	0	0	0	1	1
ncRv10340	0	0	0	0	0	0	0	0	0	0	0	0	1	0	0	1
ncRv10349	0	0	0	1	0	0	0	0	0	0	0	1	1	0	0	3
ncRv10353A	0	0	0	0	0	0	0	0	0	0	0	0	1	1	0	2
ncRv10353B	0	0	0	0	0	0	0	0	0	0	0	0	1	1	0	2
ncRv10397	0	0	0	0	0	0	0	0	0	0	0	0	1	0	0	1
ncRv10414c	0	0	0	1	0	0	0	0	0	0	0	0	0	1	1	3
ncRv10420c	0	0	1	0	0	0	0	0	0	0	0	0	0	0	0	1
ncRv10426c	0	0	0	0	0	0	0	0	0	0	0	0	1	1	1	3
ncRv10429c	0	1	0	0	0	0	0	0	0	0	0	0	0	0	0	1
ncRv10439c	1	0	1	1	1	1	1	1	1	1	1	1	1	1	1	14
ncRv10443	1	1	1	1	1	1	0	0	0	0	0	0	0	0	0	6
ncRv10466	0	0	1	0	0	0	0	0	0	0	0	0	1	0	0	2
ncRv10468	0	0	0	0	0	0	0	0	1	1	0	1	0	0	0	3
ncRv10474	0	0	0	0	0	0	0	0	0	0	0	0	1	0	0	1
ncRv10475	0	0	0	0	0	1	0	0	0	0	0	0	0	0	0	1

Table S4c. Binary matrix

ncRv10484c	1	0	1	1	0	0	0	0	0	0	0	1	1	1	1	7
ncRv10518	0	0	0	0	0	0	1	0	0	0	0	0	0	0	0	1
ncRv10537cA	1	0	0	0	0	0	1	1	1	1	1	0	0	1	0	6
ncRv10537cB	0	0	0	0	0	0	1	1	1	1	1	0	0	1	0	6
ncRv10563	0	0	0	0	0	0	1	0	0	1	1	0	1	0	0	4
ncRv10571c	0	0	0	0	0	0	1	0	0	0	0	1	0	0	1	3
ncRv10574c	0	0	0	0	0	0	0	0	0	0	0	0	0	1	1	2
ncRv10578c	0	0	1	0	0	1	0	0	0	0	0	0	0	1	1	4
ncRv10597cA	1	0	0	0	1	0	0	0	1	1	1	0	0	1	0	6
ncRv10597cB	0	0	0	0	1	0	0	0	1	1	1	0	0	1	0	6
ncRv10609A	0	0	0	0	0	0	0	0	0	0	0	0	0	1	0	1
ncRv10650A	1	0	0	0	0	1	0	1	1	1	1	0	0	0	0	6
ncRv10650B	0	0	0	0	0	1	0	1	1	1	1	0	0	0	0	6
ncRv10655A	0	1	1	0	1	1	0	0	0	0	0	0	0	0	0	4
ncRv10655B	0	1	1	0	1	1	0	0	0	0	0	0	0	0	0	4
ncRv10658c	1	1	1	1	1	0	0	1	1	1	0	0	0	0	0	8
ncRv10666	1	0	1	1	0	1	1	1	1	1	1	1	1	1	1	13
ncRv10668A	1	1	1	0	1	1	1	1	1	1	1	0	0	0	0	10
ncRv10668B	0	1	1	0	1	1	1	1	1	1	1	0	0	0	0	10
ncRv10668C	1	1	1	0	1	1	1	1	1	1	1	0	0	0	0	10
ncRv10680c	1	0	0	0	0	0	1	0	0	0	0	0	1	0	0	3
ncRv10681A	1	0	0	0	0	0	1	1	1	1	1	1	1	1	1	10
ncRv10681B	0	0	0	0	0	0	1	1	1	1	1	1	1	1	1	10
ncRv10684	1	1	1	0	1	1	1	0	0	0	0	1	1	1	1	10
ncRv10689cA	0	0	0	0	0	0	1	0	0	0	0	0	0	0	1	2
ncRv10689cB	0	0	0	0	0	0	1	0	0	0	0	0	0	0	1	2
ncRv10694	0	0	1	0	0	0	0	0	0	0	0	0	0	1	0	2
ncRv10699	1	1	1	0	1	1	1	1	1	1	1	1	1	1	1	14
ncRv10713A	0	0	0	0	0	1	1	0	0	0	0	0	0	0	0	2

Table S4c. Binary matrix

ncRv10713B	0	0	0	0	0	1	1	0	0	0	0	0	0	0	0	2
ncRv10749A	0	0	0	0	0	0	1	0	0	0	0	0	0	0	0	1
ncRv10753c	0	0	0	0	0	0	0	0	0	0	0	1	1	0	0	2
ncRv10755c	0	0	0	0	0	0	0	0	0	0	0	0	0	0	1	1
ncRv10756c	0	0	0	0	0	0	0	0	0	0	0	0	1	0	0	1
ncRv10810c	0	1	1	1	1	0	0	0	0	0	0	0	0	0	0	4
ncRv10813c	0	1	1	1	1	1	0	0	0	0	0	1	1	0	1	8
ncRv10815c	0	0	1	1	0	1	1	1	1	1	1	1	1	0	0	10
ncRv10817c	0	0	1	1	1	1	0	0	0	0	0	0	0	0	0	4
ncRv10833	0	0	1	1	0	0	0	0	0	0	0	1	1	1	1	6
ncRv10841	0	1	1	1	1	1	0	0	0	0	0	0	1	1	1	8
ncRv10847	0	0	0	0	0	1	1	0	0	0	0	1	1	0	0	4
ncRv10858c	0	0	1	1	0	0	0	0	0	0	0	0	1	0	1	4
ncRv10867c	1	0	1	1	1	1	1	1	1	1	1	1	1	0	0	12
ncRv10871	1	0	1	1	0	0	0	1	1	0	1	0	0	0	0	6
ncRv10884c	0	0	0	0	0	0	0	0	0	0	0	0	1	0	0	1
ncRv10887cA	0	0	0	0	0	0	0	0	0	0	0	0	0	0	1	1
ncRv10887cB	0	0	0	0	0	0	0	0	0	0	0	0	0	0	1	1
ncRv10922	0	0	0	0	0	0	1	1	1	1	1	1	1	0	0	7
ncRv10932c	1	0	0	1	0	1	0	0	0	0	0	0	0	1	1	4
ncRv10950c	1	0	0	0	0	0	0	0	0	0	0	0	0	0	0	1
ncRv10953c	0	0	1	0	0	0	0	0	0	0	0	0	0	0	0	1
ncRv11005c	1	0	0	0	0	0	0	0	0	0	0	1	0	1	1	3
ncRv11012	1	0	0	0	0	0	0	0	0	0	0	0	1	0	0	2
ncRv11015c	0	0	0	0	0	1	0	0	0	0	0	0	0	0	0	1
ncRv11026	0	0	0	0	0	1	0	0	0	0	0	0	0	0	0	1
ncRv11051c	1	0	0	0	0	0	0	0	0	0	0	0	0	0	0	1
ncRv11052A	0	0	0	0	0	0	1	1	1	1	0	0	0	0	0	4
ncRv11052B	0	0	0	0	0	0	1	1	1	1	0	0	0	0	0	4

Table S4c. Binary matrix

ncRv11052C	0	0	0	0	0	0	1	1	1	1	0	0	0	0	0	4
ncRv11052D	0	0	0	0	0	0	1	1	1	1	0	0	0	0	0	4
ncRv11064c	0	0	1	1	0	1	0	0	0	0	0	0	1	0	0	4
ncRv11071c	1	1	1	1	1	1	1	0	0	1	0	0	1	0	1	10
ncRv11072	0	1	1	1	1	1	0	0	0	1	1	0	0	0	1	8
ncRv11075c	1	0	0	0	0	0	0	0	0	0	0	0	0	1	0	1
ncRv11077	0	0	0	1	0	1	0	0	0	0	0	0	0	0	1	3
ncRv11080c	1	0	1	1	0	1	0	0	0	0	0	1	1	1	1	8
ncRv11094	1	0	1	1	1	1	0	1	1	1	1	1	1	0	0	11
ncRv11101c	0	0	0	0	0	0	0	0	0	0	0	1	1	0	0	2
ncRv11133c	1	1	1	1	1	1	1	1	1	1	1	1	1	1	1	15
ncRv11147A	0	0	0	0	0	1	1	1	1	1	1	0	0	1	1	8
ncRv11147B	1	0	0	0	0	1	1	1	1	1	1	0	0	1	1	8
ncRv11147C	0	0	0	0	0	1	1	1	1	1	1	0	0	1	1	8
ncRv11156A	1	0	0	0	0	0	1	1	1	1	1	1	0	0	0	7
ncRv11156B	0	0	0	0	0	0	1	1	1	1	1	1	0	0	0	7
ncRv11160	1	0	1	1	0	1	1	1	1	1	1	1	1	1	1	13
ncRv11169c	0	0	0	1	0	1	1	0	0	0	0	0	0	0	0	3
ncRv11172c	1	1	1	1	1	1	0	0	0	0	0	1	0	0	1	8
ncRv11173	0	1	1	1	1	1	0	0	1	1	1	0	1	0	0	9
ncRv11174c	0	0	0	0	0	0	1	0	0	0	0	1	1	0	0	3
ncRv11176c	0	1	0	0	1	1	0	0	0	0	0	1	1	1	1	7
ncRv11179cA	0	0	1	0	0	1	0	0	0	0	0	0	0	0	1	3
ncRv11179cB	0	0	1	0	0	1	0	0	0	0	0	0	0	0	1	3
ncRv11184c	0	0	0	0	1	1	0	0	0	0	0	0	0	0	0	2
ncRv11194c	0	0	1	0	0	0	0	0	0	0	0	1	0	1	0	3
ncRv11199c	1	0	1	1	0	1	1	1	1	1	1	1	1	0	1	12
ncRv11221	0	0	1	1	0	0	1	0	0	0	0	1	1	0	0	5
ncRv11248c	0	0	0	0	0	0	0	0	0	0	0	0	0	1	1	2

Table S4c. Binary matrix

ncRv11268c	0	0	0	0	0	1	0	0	0	0	0	0	0	0	0	1
ncRv11296	1	1	1	0	0	1	1	1	1	1	1	1	1	1	1	13
ncRv11297	1	0	0	0	0	1	0	0	1	1	1	0	0	0	0	5
ncRv11302	0	0	0	0	0	0	1	0	0	0	0	1	1	0	0	3
ncRv11323	0	0	1	1	0	0	0	0	0	0	0	0	0	0	0	2
ncRv11327c	0	0	0	0	0	0	0	0	0	0	0	0	0	1	0	1
ncRv11351	0	0	0	0	0	1	0	0	0	0	0	0	0	0	0	1
ncRv11368	0	0	0	0	0	1	0	0	0	0	0	0	0	0	0	1
ncRv11374c	0	0	0	0	0	1	1	1	1	1	1	1	1	0	0	8
ncRv11385	0	1	0	1	1	1	0	0	0	0	0	0	0	0	1	5
ncRv11387	1	0	1	1	0	0	0	1	1	1	1	0	0	0	0	7
ncRv11388	0	0	0	1	0	0	1	1	1	1	1	0	1	0	0	7
ncRv11396c	0	0	1	1	1	1	0	0	0	0	0	0	1	0	0	5
ncRv11405c	0	0	0	0	0	0	0	0	0	1	1	1	0	0	0	3
ncRv11435c	1	0	0	0	0	0	0	0	0	0	0	0	0	1	0	1
ncRv11440	0	0	1	0	1	1	0	0	0	0	0	0	0	0	0	3
ncRv11478	0	0	0	0	0	0	0	0	0	0	0	0	0	1	0	1
ncRv11490A	0	0	0	0	0	0	0	0	0	0	0	0	0	1	1	2
ncRv11490B	0	0	0	0	0	0	0	0	0	0	0	0	0	1	1	2
ncRv11501	0	0	0	0	0	0	0	0	0	0	0	0	0	1	0	1
ncRv11510	0	0	0	0	0	0	0	0	0	0	0	0	0	1	0	1
ncRv11534	1	1	1	1	1	1	1	1	1	1	1	1	1	1	1	15
ncRv11535	1	1	1	1	1	1	1	1	1	1	1	0	1	1	1	14
ncRv11536	0	0	0	0	0	0	0	0	0	0	0	0	0	0	1	1
ncRv11591A	0	0	0	1	0	0	0	0	0	0	0	0	0	0	0	1
ncRv11591B	0	0	0	1	0	0	0	0	0	0	0	0	0	0	0	1
ncRv11592c	0	0	0	1	0	0	0	0	0	0	0	1	1	1	0	4
ncRv11614A	0	1	0	0	0	0	0	1	0	1	1	0	0	0	0	4
ncRv11614B	0	1	0	0	0	0	0	1	0	1	1	0	0	0	0	4

Table S4c. Binary matrix

ncRv11629	1	0	0	0	0	0	0	0	0	0	0	0	0	0	0	1
ncRv11631	0	1	0	0	0	0	0	0	0	0	0	0	0	0	0	1
ncRv11640c	1	1	1	1	1	1	1	1	1	1	1	0	0	0	1	12
ncRv11645c	0	0	0	0	0	0	0	0	0	0	0	0	0	0	1	1
ncRv11698	0	0	0	1	0	1	0	0	0	0	0	0	0	0	0	2
ncRv11702cA	0	0	0	0	0	0	1	1	0	0	0	0	0	0	0	2
ncRv11702cB	0	0	0	0	0	0	1	1	0	0	0	0	0	0	0	2
ncRv11706A	0	0	1	1	0	1	0	0	0	0	0	0	0	1	0	4
ncRv11706cA	0	0	0	0	0	0	1	0	0	0	0	1	1	0	1	4
ncRv11706cB	0	0	0	0	0	0	1	0	0	0	0	1	1	0	1	4
ncRv11733c	1	0	0	1	0	1	1	0	0	0	0	1	1	1	1	7
ncRv11734c	0	0	0	0	0	1	1	0	0	0	0	0	0	0	0	2
ncRv11737cA	0	0	0	1	0	1	0	0	0	0	0	1	0	1	0	4
ncRv11737cB	1	0	0	1	0	1	0	0	0	0	0	1	0	1	0	4
ncRv11759cA	0	0	0	0	0	0	1	0	0	0	0	0	0	0	1	2
ncRv11759cB	0	0	0	0	0	0	1	0	0	0	0	0	0	0	1	2
ncRv11765c	1	0	0	0	0	0	1	1	1	1	1	0	0	0	0	6
ncRv11778c	0	0	0	0	0	0	0	0	0	0	0	0	0	1	1	2
ncRv11781cA	0	0	0	0	0	0	0	0	0	0	0	0	1	0	1	2
ncRv11781cB	0	0	0	0	0	0	0	0	0	0	0	0	1	0	1	2
ncRv11790	0	0	0	0	0	0	0	0	0	0	0	1	1	0	0	2
ncRv11794	0	0	1	1	0	1	1	1	1	1	1	1	1	1	1	12
ncRv11803c	0	0	0	0	0	0	0	0	0	0	0	1	0	0	0	1
ncRv11804c	1	0	1	1	0	1	0	0	0	0	0	0	1	1	1	7
ncRv11806	0	0	0	0	0	0	0	0	0	0	0	1	0	0	1	2
ncRv11806B	0	0	0	0	0	0	0	0	0	0	0	1	0	0	1	2
ncRv11808	0	0	0	0	0	0	0	0	0	0	0	0	0	0	1	1
ncRv11809A	1	0	0	0	0	1	0	0	1	0	1	1	1	0	1	7
ncRv11809B	0	0	0	0	0	1	0	0	1	0	1	1	1	0	1	7

Table S4c. Binary matrix

ncRv11810	1	0	1	1	0	1	0	0	0	0	0	0	0	0	0	4
ncRv11812c	0	0	0	0	0	1	1	0	0	0	0	1	1	1	0	5
ncRv11813c	1	0	0	0	0	0	1	1	0	1	1	1	1	1	1	9
ncRv11814	0	1	0	0	1	0	0	0	0	0	0	0	0	0	0	2
ncRv11818c	1	0	0	0	0	0	0	0	0	0	0	0	0	0	0	1
ncRv11826	1	1	1	1	1	1	1	1	1	1	1	0	0	1	1	13
ncRv11828	0	0	0	0	0	1	0	0	0	0	0	0	0	1	1	3
ncRv11829A	0	0	0	0	1	1	1	0	0	0	0	0	0	0	1	4
ncRv11829B	0	0	0	0	1	1	1	0	0	0	0	0	0	0	1	4
ncRv11829C	0	0	0	0	1	1	1	0	0	0	0	0	0	0	1	4
ncRv11829D	0	0	0	0	1	1	1	0	0	0	0	0	0	0	1	4
ncRv11832	0	0	1	1	0	0	0	0	0	0	0	0	0	0	0	2
ncRv11846c	0	0	1	1	0	0	0	0	0	0	0	0	1	0	1	4
ncRv11856c	0	0	0	1	0	0	0	0	0	0	0	0	0	0	1	2
ncRv11875A	0	0	1	1	0	0	1	0	0	0	0	0	1	0	0	4
ncRv11875B	0	0	1	1	0	0	1	0	0	0	0	0	1	0	0	4
ncRv11875C	0	0	1	1	0	0	1	0	0	0	0	0	1	0	0	4
ncRv11886c	0	0	0	0	0	0	1	0	0	0	0	0	0	1	1	3
ncRv11903	0	0	1	0	0	1	0	0	0	0	0	1	0	0	1	4
ncRv11914c	0	0	0	0	0	0	0	0	0	0	0	0	0	0	1	1
ncRv11941	0	0	0	0	0	0	0	0	0	0	0	0	0	1	0	1
ncRv11976cA	0	0	0	0	0	0	0	0	0	0	0	0	1	0	1	2
ncRv11976cB	0	0	0	0	0	0	0	0	0	0	0	0	1	0	1	2
ncRv11979c	1	1	1	0	1	1	0	0	0	0	0	1	0	0	0	6
ncRv11980c	0	0	1	1	0	1	0	0	0	0	0	1	0	1	1	6
ncRv11984c	0	0	0	0	0	0	1	0	0	0	0	0	0	0	0	1
ncRv11985c	0	0	0	0	0	0	0	0	0	0	0	0	0	0	1	1
ncRv11986A	0	0	1	1	0	0	0	0	0	0	0	0	0	0	0	2
ncRv11986B	0	0	1	1	0	0	0	0	0	0	0	0	0	0	0	2

Table S4c. Binary matrix

ncRv11987	0	0	0	0	0	0	0	0	0	0	0	0	0	1	1	2
ncRv11988	0	0	0	1	0	1	0	0	0	0	0	0	0	0	0	2
ncRv11990c	0	0	0	0	0	0	0	0	0	0	0	0	0	0	1	1
ncRv11996A	0	0	0	0	0	0	1	0	0	0	0	1	0	0	0	2
ncRv11996B	0	0	0	0	0	0	1	0	0	0	0	1	0	0	0	2
ncRv12031c	0	0	0	0	0	1	0	0	0	0	0	0	0	0	0	1
ncRv12049cA	1	0	0	1	0	0	0	0	0	0	0	0	1	0	1	4
ncRv12049cB	0	0	0	1	0	0	0	0	0	0	0	0	1	0	1	4
ncRv12075c	0	0	0	0	0	0	1	0	0	0	0	0	0	0	0	1
ncRv12080	1	0	0	0	0	1	0	0	0	0	0	0	0	0	0	2
ncRv12081c	1	0	1	0	0	1	1	0	0	0	0	0	0	0	1	5
ncRv12108	0	0	1	1	0	0	0	0	0	0	0	0	0	0	0	2
ncRv12111c	0	0	0	0	0	1	1	0	0	0	0	0	0	0	0	2
ncRv12115c	0	0	0	0	0	0	0	0	0	0	0	0	0	0	1	1
ncRv12126c	0	0	0	0	0	1	0	0	0	0	0	0	0	1	1	3
ncRv12145cA	0	0	0	0	0	0	0	0	0	0	0	1	1	0	1	3
ncRv12145cB	0	0	0	0	0	0	0	0	0	0	0	1	1	0	1	3
ncRv12146c	1	0	0	0	0	1	0	0	0	0	1	0	0	0	0	3
ncRv12161c	1	0	0	0	0	0	0	1	1	1	0	0	0	0	0	4
ncRv12169c	0	0	0	0	0	0	0	0	0	0	0	1	1	0	1	3
ncRv12190c	0	0	1	0	0	0	0	0	0	0	0	0	0	1	0	2
ncRv12198c	0	0	0	0	1	1	0	0	1	1	1	0	0	0	1	6
ncRv12200c	0	0	1	1	1	1	0	0	0	0	0	0	0	0	0	4
ncRv12202c	0	0	0	0	0	0	1	1	0	0	0	0	0	0	1	3
ncRv12214c	0	0	0	0	0	0	0	0	0	0	0	0	1	0	0	1
ncRv12219A	1	0	0	0	0	0	0	0	0	0	0	0	0	0	0	1
ncRv12220	1	0	1	0	1	1	0	1	1	1	1	0	0	0	1	9
ncRv12225	0	0	0	0	0	0	1	0	0	0	0	0	0	0	0	1
ncRv12301	1	0	0	0	0	0	0	0	0	0	0	0	0	0	1	2

Table S4c. Binary matrix

ncRv12307c	0	0	0	0	0	0	0	0	0	0	0	0	0	0	1	1
ncRv12327A	0	0	1	0	0	0	0	0	0	0	0	0	0	0	1	2
ncRv12327B	0	0	1	0	0	0	0	0	0	0	0	0	0	0	1	2
ncRv12329c	0	0	0	0	0	0	0	0	0	0	0	1	0	0	0	1
ncRv12333cA	0	0	1	0	1	0	0	0	1	1	1	0	0	0	0	5
ncRv12333cB	0	0	1	0	1	0	0	0	1	1	1	0	0	0	0	5
ncRv12357c	0	0	0	0	0	0	0	0	0	0	0	0	1	0	0	1
ncRv12359	0	0	0	0	0	0	1	0	0	0	0	0	0	0	0	1
ncRv12374c	1	0	0	0	0	0	0	0	0	0	1	0	0	0	0	2
ncRv12386cA	0	0	0	0	0	1	0	0	1	1	1	0	0	0	1	5
ncRv12386cB	0	0	0	0	0	1	0	0	1	1	1	0	0	0	1	5
ncRv12390cA	1	1	1	1	1	1	1	1	1	1	1	1	1	0	1	14
ncRv12390cB	0	1	1	1	1	1	1	1	1	1	1	1	1	0	1	14
ncRv12395	1	1	1	0	0	1	0	0	0	0	0	0	1	0	0	5
ncRv12395A	0	1	0	0	0	0	0	0	0	0	0	0	1	0	0	2
ncRv12411c	0	0	0	0	0	1	0	0	0	0	0	0	0	0	0	1
ncRv12442c	0	0	0	0	0	0	0	0	0	0	0	1	1	0	0	2
ncRv12444c	1	0	0	1	0	1	1	1	1	1	1	1	1	1	1	12
ncRv12455c	0	0	0	1	0	0	0	0	0	0	0	1	0	0	0	2
ncRv12457cA	0	0	1	0	0	0	0	0	0	0	0	1	0	1	0	3
ncRv12457cB	0	0	1	0	0	0	0	0	0	0	0	1	0	1	0	3
ncRv12459	0	0	1	0	0	0	0	0	0	0	0	0	1	1	1	4
ncRv12461c	1	0	1	0	0	0	0	0	0	0	0	0	0	1	1	4
ncRv12484c	0	0	0	0	0	0	0	0	0	0	0	0	1	0	0	1
ncRv12497cA	0	0	1	0	0	0	1	1	1	0	0	1	1	0	1	7
ncRv12497cB	0	0	1	0	0	0	1	1	1	0	0	1	1	0	1	7
ncRv12512cA	0	0	0	0	0	0	0	0	0	0	0	1	0	0	1	2
ncRv12512cB	0	0	0	0	0	0	0	0	0	0	0	1	0	0	1	2
ncRv12519	0	0	0	0	0	0	0	0	0	0	0	1	0	0	0	1

Table S4c. Binary matrix

ncRv12524cA	1	0	0	0	0	0	0	0	0	1	1	1	1	1	0	5
ncRv12524cB	0	0	0	0	0	0	0	0	0	1	1	1	1	1	0	5
ncRv12542	0	0	0	0	0	0	1	1	1	1	1	0	0	0	0	5
ncRv12560	1	0	0	0	0	0	0	0	0	0	0	0	0	0	0	1
ncRv12589	0	0	1	0	0	0	0	0	0	0	0	0	0	0	0	1
ncRv12615cA	0	0	0	0	0	0	0	0	0	0	0	0	1	0	0	1
ncRv12615cB	0	0	0	0	0	0	0	0	0	0	0	0	1	0	0	1
ncRv12617c	0	0	0	0	0	0	0	0	0	0	0	0	1	0	0	1
ncRv12622	0	0	0	0	0	1	0	0	0	0	0	0	0	0	0	1
ncRv12626cA	0	0	0	0	0	0	1	0	0	0	0	1	0	0	0	2
ncRv12626cB	0	0	0	0	0	0	1	0	0	0	0	1	0	0	0	2
ncRv12628	1	0	0	1	0	1	1	1	0	1	1	1	1	1	1	10
ncRv12632c	0	0	0	0	0	1	0	0	0	0	0	1	1	0	1	4
ncRv12637	0	0	0	1	0	1	0	0	0	0	0	0	0	0	0	2
ncRv12664	0	0	0	1	0	0	0	0	0	0	0	0	1	1	1	4
ncRv12688c	0	0	0	0	0	0	0	0	0	0	0	0	1	0	1	2
ncRv12694c	0	0	0	1	0	0	0	0	0	0	0	0	0	0	0	1
ncRv12702	1	0	1	1	0	1	0	0	0	0	0	1	1	0	1	7
ncRv12706c	0	0	0	0	0	0	1	0	0	0	0	0	0	0	0	1
ncRv12709	0	0	1	0	0	1	0	0	0	0	0	0	0	0	0	2
ncRv12710	1	0	0	0	0	0	1	1	1	0	0	1	0	0	0	5
ncRv12744c	1	0	0	0	0	0	1	0	0	0	0	1	1	0	1	5
ncRv12783cA	1	0	0	0	0	0	1	0	0	0	0	0	0	1	0	3
ncRv12783cB	0	0	0	0	0	0	1	0	0	0	0	0	0	1	0	3
ncRv12792c	0	0	0	0	0	0	0	0	0	0	0	0	0	1	1	2
ncRv12808	0	0	0	0	0	0	0	0	0	0	0	0	0	1	1	2
ncRv12813A	0	0	0	0	0	0	0	0	0	0	0	0	0	0	1	1
ncRv12813B	0	0	0	0	0	0	0	0	0	0	0	0	0	0	1	1
ncRv12813C	0	0	0	0	0	0	0	0	0	0	0	0	0	0	1	1

Table S4c. Binary matrix

ncRv12842c	0	0	0	1	1	0	0	0	0	0	0	0	0	1	1	4
ncRv12866	0	0	1	1	0	0	1	0	0	0	0	0	0	0	0	3
ncRv12890c	1	0	0	0	0	0	1	1	1	1	1	1	1	0	0	8
ncRv12898c	0	0	0	0	0	0	0	0	0	0	0	0	0	1	0	1
ncRv12904cA	1	1	1	1	1	1	0	0	0	0	0	1	1	0	1	9
ncRv12904cB	1	1	1	1	1	1	0	0	0	0	0	1	1	0	1	9
ncRv12909c	0	0	0	0	0	0	0	0	0	0	0	1	1	0	0	2
ncRv12929	1	0	1	1	0	1	1	1	1	1	1	1	1	0	1	12
ncRv12939	1	0	0	0	1	1	0	1	1	1	0	0	0	1	1	8
ncRv12940c	0	0	1	0	0	0	0	0	0	0	0	1	1	1	1	5
ncRv12942	0	0	0	0	0	0	0	1	1	0	1	0	0	0	0	3
ncRv12950c	0	0	1	0	0	1	0	0	0	0	0	1	0	1	1	5
ncRv12953	0	0	0	0	1	0	0	0	0	0	0	0	0	0	0	1
ncRv12958c	0	0	0	0	0	0	0	0	0	0	0	1	0	0	0	1
ncRv12970c	0	0	0	0	0	0	1	0	0	0	0	0	0	0	0	1
ncRv12990c	1	1	1	1	1	1	0	1	1	1	1	0	0	0	1	11
ncRv13000	1	0	0	0	0	0	1	1	1	1	1	1	1	0	1	9
ncRv13003c	0	0	0	0	0	0	0	0	0	0	0	0	0	1	1	2
ncRv13027c	1	0	1	1	1	0	0	0	0	0	0	0	0	0	0	4
ncRv13034c	0	0	0	0	0	0	0	0	0	0	0	0	0	1	0	1
ncRv13053c	1	0	1	1	1	1	1	1	1	1	1	1	1	1	1	14
ncRv13059	0	0	0	0	0	0	0	0	0	0	0	0	1	0	0	1
ncRv13061c	0	0	0	0	0	0	0	0	0	0	0	0	0	0	1	1
ncRv13124	0	0	0	0	0	0	0	0	0	0	0	0	0	0	1	1
ncRv13130c	0	0	0	0	0	1	0	0	0	0	0	0	0	0	1	2
ncRv13134c	1	0	0	0	0	1	1	1	1	1	1	1	1	0	0	9
ncRv13171c	0	0	0	1	1	1	1	0	0	0	0	0	0	0	0	4
ncRv13176c	0	0	0	0	0	0	0	0	0	0	0	0	0	0	1	1
ncRv13197AA	1	0	0	0	0	0	1	1	0	0	0	1	0	0	0	4

Table S4c. Binary matrix

ncRv13197AB	0	0	0	0	0	0	1	1	0	0	0	1	0	0	0	4
ncRv13202cA	0	0	0	0	0	0	0	0	0	0	0	0	1	0	0	1
ncRv13202cB	0	0	0	0	0	0	0	0	0	0	0	0	1	0	0	1
ncRv13207c	1	0	0	0	0	0	0	0	0	0	1	0	0	0	0	2
ncRv13208A	0	0	0	0	0	0	0	0	0	0	0	0	0	1	0	1
ncRv13210c	1	0	1	1	0	0	1	1	1	1	1	1	1	1	1	12
ncRv13218	0	1	1	1	1	1	0	1	1	1	1	1	1	1	1	13
ncRv13221c	1	1	1	1	1	1	1	1	1	1	1	1	1	1	1	15
ncRv13223c	0	0	0	0	0	0	1	1	1	1	0	1	1	0	1	7
ncRv13241c	1	0	1	1	0	1	1	0	1	1	1	1	1	0	1	11
ncRv13260c	1	1	1	1	1	0	0	0	0	0	0	1	1	0	0	7
ncRv13287c	0	0	0	0	0	0	1	0	0	0	0	1	1	0	0	3
ncRv13303c	0	0	0	0	0	0	0	1	0	0	0	0	0	0	0	1
ncRv13332A	1	0	0	0	0	0	0	0	0	0	0	0	0	0	0	1
ncRv13332B	0	0	0	0	0	0	0	0	0	0	0	0	0	0	0	1
ncRv13339c	1	0	0	1	0	0	1	0	0	0	1	1	1	0	0	6
ncRv13401	0	0	1	1	0	0	0	0	0	0	0	0	0	0	0	2
ncRv13402cA	0	0	1	1	0	0	0	0	0	0	0	0	0	0	0	2
ncRv13402cB	0	0	1	1	0	0	0	0	0	0	0	0	0	0	0	2
ncRv13411c	0	0	0	1	0	0	0	0	0	0	0	0	0	0	0	1
ncRv13415c	0	0	0	1	0	1	0	0	0	0	0	0	1	0	0	3
ncRv13418c	1	0	0	1	1	1	1	1	1	1	1	1	1	1	1	13
ncRv13424c	0	0	0	0	0	0	0	1	1	1	0	0	0	0	0	3
ncRv13425	0	0	0	0	0	0	0	1	1	1	1	0	0	0	0	4
ncRv13443c	0	0	0	1	0	1	1	0	0	0	0	1	0	0	0	4
ncRv13460c	1	1	1	1	1	1	1	1	1	1	1	1	1	1	1	15
ncRv13462c	1	1	1	1	1	1	1	1	1	1	1	1	1	1	0	14
ncRv13476c	0	1	1	0	0	1	0	0	0	0	0	1	1	0	1	6
ncRv13478	0	1	1	1	1	1	0	1	1	1	1	1	1	1	1	13

Table S4c. Binary matrix

ncRv13483c	0	0	1	1	0	0	0	0	0	0	0	0	0	0	0	2
ncRv13487c	1	1	1	0	1	1	0	1	1	1	1	1	1	1	1	13
ncRv13490	0	0	1	1	1	0	0	0	0	0	0	0	0	0	0	3
ncRv13503c	0	0	0	0	0	0	0	0	0	0	0	0	0	0	1	1
ncRv13520c	1	0	0	0	0	0	1	1	1	1	1	0	1	1	0	7
ncRv13555c	0	0	0	0	0	0	1	0	0	0	0	0	0	0	0	1
ncRv13573c	0	0	0	0	0	0	0	0	0	0	0	1	1	0	1	3
ncRv13595c	0	0	1	0	0	0	0	0	0	0	0	0	0	1	1	3
ncRv13596c	1	0	1	0	0	0	1	0	0	0	0	1	1	0	0	5
ncRv13614c	0	0	0	0	1	1	0	0	0	0	0	0	1	0	0	3
ncRv13616cA	0	0	0	1	0	0	0	0	0	0	0	0	0	1	1	3
ncRv13616cB	0	0	0	1	0	0	0	0	0	0	0	0	0	1	1	3
ncRv13632	1	0	0	0	0	1	0	0	0	0	0	1	1	1	0	5
ncRv13646c	1	1	1	0	0	1	0	0	0	0	0	1	0	1	0	5
ncRv13647c	1	1	0	0	1	0	0	0	0	0	0	0	0	0	0	3
ncRv13648c	1	1	1	1	1	1	1	1	1	1	1	1	1	1	1	15
ncRv13660c	1	1	1	1	1	1	1	1	1	1	1	0	0	1	1	13
ncRv13661	1	1	1	1	1	1	1	1	1	1	1	1	1	1	1	15
ncRv13680	1	0	1	1	0	1	1	0	0	0	0	0	0	0	0	5
ncRv13681cA	0	0	0	1	0	0	0	0	0	0	0	0	0	0	1	2
ncRv13681cB	0	0	0	1	0	0	0	0	0	0	0	0	0	0	1	2
ncRv13687c	0	0	0	0	0	0	1	1	1	1	1	0	0	0	0	5
ncRv13709c	1	0	0	0	0	0	1	0	0	0	0	0	0	1	1	4
ncRv13723	0	0	0	0	1	0	0	0	0	0	0	0	0	0	0	1
ncRv13725	1	1	1	0	1	1	0	1	1	1	1	1	0	1	1	12
ncRv13739c	0	0	0	0	0	0	0	1	1	1	1	1	0	0	0	5
ncRv13750c	0	0	1	1	0	0	0	0	0	0	0	0	0	0	1	3
ncRv13801c	1	0	1	1	0	1	1	1	1	1	1	1	1	0	1	12
ncRv13802c	1	0	1	1	0	0	0	0	0	0	0	1	0	0	0	4

Table S4c. Binary matrix

ncRv13803c	1	0	0	0	0	0	0	0	1	1	1	0	1	0	0	5
ncRv13804c	0	1	1	1	1	1	0	0	0	0	0	1	1	0	0	7
ncRv13810	1	1	0	0	1	0	0	0	0	0	0	0	0	1	0	4
ncRv13822A	1	1	1	0	0	1	0	1	1	1	1	1	0	1	1	11
ncRv13822B	0	1	1	0	0	1	0	1	1	1	1	1	0	1	1	11
ncRv13823c	0	0	1	0	0	0	0	0	0	0	0	0	0	0	1	2
ncRv13836A	0	0	0	0	0	0	1	0	0	0	0	0	1	0	0	2
ncRv13836B	0	0	0	0	0	0	1	0	0	0	0	0	1	0	0	2
ncRv13846	0	0	0	0	0	1	0	0	0	0	0	1	0	0	1	3
ncRv13847	0	0	0	0	0	1	1	0	0	0	0	0	1	1	1	5
ncRv13848	0	0	0	0	0	0	0	0	0	0	0	1	0	0	0	1
ncRv13849	1	0	1	0	1	1	0	0	0	0	0	0	0	0	0	4
ncRv13855A	0	0	0	0	0	0	0	1	1	1	1	0	0	0	1	5
ncRv13855B	0	0	0	0	0	0	0	1	1	1	1	0	0	0	1	5
ncRv13859c	1	0	0	0	0	0	0	0	0	1	0	1	1	0	1	5
ncRv13870	1	0	1	0	0	0	1	0	0	0	0	1	0	1	0	5
ncRv13871	0	0	1	0	0	1	1	1	1	1	1	1	1	1	1	11
ncRv13875	0	1	1	1	0	1	0	0	0	0	0	0	0	1	0	5
ncRv13879cA	0	0	0	0	0	0	0	1	0	0	0	0	1	0	0	2
ncRv13879cB	0	0	0	0	0	0	0	1	0	0	0	0	1	0	0	2
ncRv13879cC	0	0	0	0	0	0	0	1	0	0	0	0	1	0	0	2
ncRv13901c	0	0	0	0	0	0	0	0	0	0	0	0	0	1	1	2
ncRv13907c	0	0	0	0	0	0	0	1	1	1	1	0	0	0	0	4
ncRv13916cA	1	0	0	1	0	0	0	0	0	0	0	1	0	0	1	4
ncRv13916cB	0	0	0	1	0	0	0	0	0	0	0	1	0	0	1	4
ncRv13919c	1	1	1	0	0	0	0	0	0	0	0	0	0	0	0	3

Table S4d. Context-dependent expression of sRNAs

sRNA ID	SRR8550302	SRR1917703	SRR1917706	SRR1917709	SRR1917712	SRR1917714	SRR3725585	SRR3725588	SRR3725591	SRR3725594	SRR3725597	SRR998928	SRR998931	SRR568038	SRR568040
ncRv10001	183.28	502.06	348.29	280.95	410.17	202.5	42.68	235.52	171.05	159.54	143.18	180.89	73.34	1613.26	303.69
ncRv10009	2705.39	2600.02	797.3	750.23	3305.83	809.24	583.01	2693.48	2224.96	1871.45	1628.7	747.56	143.27	588.29	22.78
ncRv10010c	405	893.43	452.07	334.89	737.73	438.5	545.07	1495.45	1224.89	1254.14	1364.77	607.28	230.26	596.38	129.07
ncRv10019c	1172.1	1395.57	798.77	747.94	1573.15	362.88	859.55	1723.44	1601.44	1564.95	1535.4	1525.88	725.46	1948.85	131.6
ncRv10049	823.34	626.51	456.98	876.98	626.83	211.64	857.97	718.79	708.88	649.85	574.24	302.1	413.9	68.74	11.39
ncRv10052	659.81	926.06	443.59	528.01	738.78	417.69	1966.34	980.25	1072.74	1126.93	808.05	856.46	307.01	171.16	50.62
ncRv10053	1763.51	1075.69	457.49	415.03	1003.46	297.66	1815.74	1034.62	1153.95	1110.16	662.11	2320.2	975.62	1307.99	242.95
ncRv10061c	328.56	250.77	191.05	236.67	222.58	131.19	780.82	695.11	913.89	827.71	700.72	1627.4	736.83	458.24	88.58
ncRv10062	911.48	310.34	49.51	74.84	338.34	25.88	154.92	444.43	470.39	471.01	352.41	238.11	102.34	125.34	22.78
ncRv10070cA	307.55	634.99	256.72	347.68	533.56	997.28	196.02	180.74	263.7	274.06	268.84	354.4	105.75	188.01	121.48
ncRv10070cB	480.44	453.17	164.4	232.23	489.17	545.08	110.26	131.15	218.75	295.41	285.12	1018.9	312.98	541.29	419.48
ncRv10071A	741.16	409.26	291.99	216.4	247.58	181.18	608.3	1344.11	1301.03	1434.53	1222.83	275.03	126.22	663.09	136.66
ncRv10071B	332.14	1190.47	751.18	380.73	793.77	412.61	325.33	1350.61	1158.16	1150.21	1025.27	179.05	69.93	475.08	167.03
ncRv10071C	193.56	589.23	417.81	220.45	365.71	155.3	273.17	1396.41	1061.29	1000.27	826.79	107.06	44.35	181.95	37.96
ncRv10107cA	368.79	1357.4	2313.91	900.21	1115.01	1261.44	187.17	276.06	245.89	298.56	292.8	308.25	185.91	562.01	45.55
ncRv10107cB	1232.74	2783.13	3311.3	1548.07	3188.66	1911.07	289.92	441.95	577.08	743.42	828.43	735.25	481.56	355.13	118.95
ncRv10115A	320.52	299.6	206.53	206.73	344.13	772.7	182.74	263.07	348.91	338.94	314	579.59	272.9	244.62	212.59
ncRv10115B	128.85	110.75	78.26	91.52	119.97	265.5	127.41	150.1	158.5	138.94	117.29	636.81	266.08	51.55	85.41
ncRv10116c	38.89	135.23	139.38	281.88	128.39	162.91	47.11	63.75	90.38	75.47	57.45	55.37	29	198.12	630.16
ncRv10123	167.63	133.44	125.14	99.78	154.44	146.17	381.93	238.31	220.29	182.38	135.8	201.19	431.53	84.91	60.74
ncRv10128	1018.92	1084.38	411.48	150.71	1399.33	210.11	172.2	248	613.58	665.52	434.85	473.76	284.27	188.69	40.49
ncRv10129c	4.92	259.98	47.48	31.18	428.85	63.19	44.9	15.47	10.37	12.58	7.99	25.84	46.05	7833.8	197.4
ncRv10135c	149.75	231.09	762.37	927.03	227.05	146.17	153.97	157.84	202.8	239.97	200.02	677.42	656.67	410.39	98.7
ncRv10165c	92.27	1139.7	301.29	153.91	469.47	988.45	53.37	152.89	90.19	111.21	177.83	94.51	23.2	188.42	118.44
ncRv10166	949.63	1401.79	351.68	189.58	1024.85	1000.07	117.82	516.33	557.97	677.99	968.84	411.62	131.33	683.31	369.49
ncRv10186A	406.79	524.21	176.8	613.45	574.43	1003.87	4313.98	633.21	481.84	293.26	249.69	5421.8	4242.49	13767.27	103134.11
ncRv10187	557.89	270.2	118.36	172.12	340.71	159.87	2112.3	709.66	590.9	568.98	464.24	1716.62	7175.61	363.89	3538.02
ncRv10190	416.18	699.92	475.13	445.9	1483.08	785.64	2986.5	1319.35	1063.89	688.8	787.46	435.61	682.25	521.58	121.48
ncRv10210	128.15	220.18	368.3	1923.7	399.38	343.59	304.78	632.39	802.13	825.5	652.99	697.72	817.57	242.6	121.48

Table S4d. Context dependent expression of sRNAs

ncRv10231	158.69	1611.75	710.15	996.88	1190.52	384.45	600.4	194.67	200.53	267.44	419.08	282.41	295.08	287.07	37.96
ncRv10242c	323.2	1041.18	1700.76	883.69	857.17	248.18	548.55	694.18	572.11	529.26	571.16	304.56	162.04	2971.79	273.32
ncRv10243	683.14	1044.81	1714.12	2374.73	451.58	225.19	476.46	608.08	579.31	611.75	682.33	310.84	315.2	702.31	935.37
ncRv10244c	87.32	410.88	1799.9	3058.92	332.03	178.14	191.81	129.16	216.19	299	334.08	489.76	835.76	287.07	40.49
ncRv10245	27.27	128.33	402.55	840.97	166.54	146.93	135.95	62.83	60.58	54.95	52.85	59.07	47.76	58.63	15.18
ncRv10251c	60.8	18.92	27.13	377.61	53.15	35.78	1786.34	296.8	139.63	147.62	111.22	3422.16	9087.63	175.88	1632.35
ncRv10279c	25.93	1541.71	378.81	965.7	77.61	26.64	13.91	17.33	10.69	15.23	24.58	22.15	29	669.16	6590.14
ncRv10281	1425.62	1203.58	1160.61	1111.23	635.8	277.32	1095.65	2382.97	1870.36	2171.61	2320.12	1237.23	692.49	7960	2590.07
ncRv10296c	213.9	766.25	1180.36	1300.74	474.89	679.44	385.88	327.59	274.56	280.68	291.11	263.95	263.52	649.95	470.72
ncRv10297	82.25	110.18	310.99	665.73	52.36	306.03	233.01	189.72	138.98	133.72	108.46	110.75	155.21	90.97	584.61
ncRv10301	240.5	788.62	589.41	642.65	570.92	248.18	1170.13	1092.49	586.69	550.78	729.7	263.95	267.78	5417.96	409.99
ncRv10323c	51.41	65.44	42.05	66.42	105.5	38.83	90.11	82.94	75.81	105.92	82.65	59.07	57.99	701.5	561.83
ncRv10340	120.25	73.37	131.92	318.36	355.45	326.59	116.67	222.21	138.66	123.13	108.46	376.55	649.85	549.88	182.22
ncRv10349	102.52	364.36	203.93	635.48	525.14	349.68	296.14	175.17	104.53	102.39	106.31	819.55	701.58	355.81	70.86
ncRv10353A	202.05	249.11	206.36	220.92	251.39	137.79	303.36	215.4	172.99	154.24	145.33	407.93	542.39	923.88	106.29
ncRv10353B	179.7	254.1	223.15	265.04	207.85	146.93	682.6	740.3	528.7	508.08	559.18	391.31	394	2733.24	242.95
ncRv10397	180.6	251.54	210.26	186.78	350.45	405.76	188.75	231.81	213.81	221.77	194.18	466.99	886.93	236.53	273.32
ncRv10414c	162.12	289.71	463.03	737.97	167.68	127.39	316.59	144.63	134.55	146.52	155.06	141.51	155.78	1332.92	997.13
ncRv10420c	118.46	417.96	520.57	482.38	223.9	98.97	320.91	413.48	260.79	198.6	148.71	321.17	114.28	99.06	0
ncRv10426c	92.09	307.95	161.88	87.31	235.39	148.2	124.78	329.3	258.52	226.4	172.26	708.8	733.42	1491.96	1371.68
ncRv10429c	531.06	1542.47	189.58	126.91	1179.73	188.04	30.35	371.08	581.51	595.13	429.22	1057.66	208.09	4647.72	98.7
ncRv10439c	6382.22	277.32	774.19	4071.88	1115.39	1817.07	4839.95	1471.84	1153.3	642.79	740.63	6522.09	3109.62	406.06	273.32
ncRv10443	645.2	848.61	747.56	795.44	819.64	303.24	471.09	545.83	482.27	519.11	482.37	238.73	158.06	349.07	45.55
ncRv10466	149.01	129.18	937.59	991.78	90.15	123.83	595.23	992.22	2218.91	2486.44	1017.59	418.39	570.82	319.42	298.63
ncRv10468	301.74	287.84	709.81	449.33	289.41	76.89	751.84	917.94	1469.16	1564.62	683.92	902.61	429.82	1077.53	75.92
ncRv10474	145.73	292.95	271.99	202.37	239.16	267.97	573.84	594.53	517.04	720.91	899.92	874.92	815.29	198.12	15.18
ncRv10475	805.09	1484.96	1296.85	1710.63	1137.37	612.83	484.05	426.78	335.95	358.14	335.51	256.57	213.2	297.18	113.88
ncRv10484c	400.18	712.29	2249	1999.24	1204.46	739.96	1727.91	1452.37	1456.4	1653.39	1799.83	869.01	1237.61	3123.82	267.25
ncRv10518	509.16	231.86	129.89	86.69	209.69	101.25	1301.34	595.46	816.7	706.01	578.85	681.11	262.67	810.67	326.47
ncRv10537cA	390.7	182.26	131.24	168.69	169.17	53.29	995.29	1729.11	1686.21	1681.79	1361.39	513.14	262.67	1332.25	121.48
ncRv10537cB	243.63	624.76	446.64	566.88	625.12	198.69	636.76	1083.52	1025.01	1048.59	842.77	409.77	283.14	2527.03	182.22

Table S4d. Context dependent expression of sRNAs

ncRv10563	337.95	249.62	149.56	233.71	209.95	87.17	2004.96	562.03	570.17	788.43	696.83	510.37	853.67	443.75	337.86
ncRv10571c	257.19	109.24	43.3	131.38	127.51	86.53	654.67	150.82	142.22	173.44	176.97	1672.32	217.18	252.7	318.88
ncRv10574c	41.57	73.62	62.74	211.41	64.46	113.43	350.31	43.02	48.92	75.14	85.11	297.18	78.46	1508.13	933.86
ncRv10578c	428.96	788.36	530.54	314.93	457.58	369.37	330.46	715.54	875.34	1208.4	1349.41	303.45	313.15	1935.1	309.77
ncRv10597cA	1848.89	723.43	277.75	160.27	1032.4	86.79	84.1	791.98	1189.26	1207.8	1384.74	258.42	141.57	3218.43	83.52
ncRv10597cB	988.1	792.76	330.72	190.08	1212.78	144.95	72.47	572.31	817.81	868	998.42	154.31	79.82	1715.15	48.59
ncRv10609A	102.37	180.48	124.12	175.24	124.97	109.62	147.97	246.66	197.62	119.49	88.18	73.83	35.82	4291.91	1457.73
ncRv10650A	1449.25	2546.33	363.89	555.97	2032.96	574.01	308.58	720.49	1434.17	1727.8	1909.52	295.33	163.74	737.89	45.55
ncRv10650B	2664.71	490.55	132.6	220.45	430.69	121.04	405.32	1286.54	2781.52	3359.6	3547.74	278.72	158.62	564.03	91.11
ncRv10655A	589.62	1293.87	744.06	514.65	920.84	512.34	425.72	211.07	246.86	417.05	754.59	303.64	139.01	200.14	91.11
ncRv10655B	178.81	711.67	509.72	324.91	362.81	514.63	307.31	177.34	147.4	143.32	262.69	166.12	102.34	58.63	15.18
ncRv10658c	651.02	808.3	705.29	476.14	707.38	139.82	388.46	809.11	787.12	776.63	947.54	452.84	271.76	456.21	17.72
ncRv10666	780.42	667.47	624.56	559.12	554.75	649.44	1618.83	970.95	977.48	914.87	740.46	1428.67	702.72	430.79	505.23
ncRv10668A	454.47	2261.99	1559.79	829.22	1327.77	417.69	447.69	891.95	785.71	634.63	624.32	178.43	68.23	161.73	20.25
ncRv10668B	705.63	863.01	482.42	237.92	490.94	173.95	760.85	1551	1246.92	1010.2	954.14	275.03	99.78	194.08	22.78
ncRv10668C	607.95	1462.97	746.77	364.2	732.99	157.58	478.04	1059.69	822.86	635.51	535.22	199.35	54.58	285.05	7.59
ncRv10680c	358.33	242.54	182.32	233.49	307.4	82.83	713.14	545.69	499.29	469.35	436.16	457.03	494.63	269.28	27.33
ncRv10681A	485.47	937.91	400.86	412.22	802.19	318.98	957.98	765.67	986.78	943.01	804.36	2251.91	837.47	1043.16	440.35
ncRv10681B	1023.24	470.62	171.6	198.78	413.33	161.77	1905.06	1411.73	1733.03	1703.96	1307.17	5011.42	2061.26	1830.58	539.05
ncRv10684	1335.15	852.27	385.51	327.41	692.74	227.62	500.1	1156.71	1238.82	948.47	646.82	4177.1	1328.69	5231.46	979.41
ncRv10689cA	342.87	57.26	92.24	163.08	48.94	44.92	2353.54	385	327.52	268.77	337.97	212.27	247.32	406.35	455.54
ncRv10689cB	236.48	121.17	133.28	178.67	112.61	68.52	2035.47	346.32	259.17	224.41	238.11	252.88	245.61	165.77	121.48
ncRv10694	189.24	595.62	1104.22	361.5	521.99	132.46	214.99	1321.72	1106.22	1233.95	1207.67	86.14	37.52	1872.02	70.86
ncRv10699	3099.21	2001.94	628.55	269.78	1398.84	912.77	1028.61	2944.29	3291.11	2797.11	2578.88	2896.47	642.68	2738.9	331.03
ncRv10713A	557.89	1524.32	574.15	350.79	801.13	457.53	568.47	723.58	818.32	915.53	658.11	616.51	144.98	564.03	144.25
ncRv10713B	1246.75	795.52	333.37	224.51	637.22	331.16	935.85	1095.59	1250.49	1334.9	1017.28	1168.41	221.73	1109.87	235.36
ncRv10749A	25.48	43.71	35.27	64.55	31.05	92.88	987.39	99.04	87.79	78.11	59.3	64.6	54.58	80.87	15.18
ncRv10753c	11.92	12.27	21.48	83.98	12.1	22.33	2428.57	67.26	40.82	30.89	19.66	1103.8	1896.67	13.48	0
ncRv10755c	77.34	217.03	623.67	542.87	157.86	854.92	323.44	233.66	139.3	138.69	147.17	127.36	59.7	121.3	531.46
ncRv10756c	175.23	185.84	177.37	246.65	197.32	146.17	426.51	357.46	303.87	275.72	289.42	736.48	839.17	1073.48	493.5
ncRv10810c	1281.17	3132.24	2768.01	3671.64	3690.48	1961.06	3393.09	2605.58	2468.9	2610.23	2572.24	1681.55	1871.08	14656.78	6423.1

ncRv10813c	1577.55	1214.63	1647	2132.2	1402.97	616.64	1129.03	969.16	949.37	753.35	538.6	740.18	1184.56	413.42	223.97
ncRv10815c	210.46	459.42	645.91	1095.47	520.36	361	845.05	589.7	647.79	573.42	437.45	587.71	873.97	243.81	78.96
ncRv10817c	266.65	655.05	605.35	565.32	664.19	250.84	396.16	635.07	587.34	729.35	602.5	178.12	173.97	962.29	159.44
ncRv10833	1879.93	593.72	469.85	852.42	443.58	140.51	1052.74	983.11	916.44	1057.39	1155.06	483.08	749.51	758.98	203.91
ncRv10841	341.23	2161.44	3129.53	5444.63	1458.44	3065.93	1574.19	1184.83	1379.32	1603.23	2171.29	628.2	902.85	696.11	873.12
ncRv10847	136.79	46.1	6.33	58	93.4	301.97	3565.08	233.35	101.4	38.4	30.62	801.09	825.53	130.73	108.82
ncRv10858c	184.47	286.65	614.74	669.16	279.94	204.02	153.66	554.4	1266.25	2109.32	2666.26	834.31	830.08	824.82	490.97
ncRv10867c	3134.03	863.29	884.61	348.06	1649.74	248.85	851.96	1217.6	2896.78	3161.81	2839.95	1633.76	387.37	318.07	23.62
ncRv10871	1256.14	674.35	716.02	865.81	444.99	301.47	1079.6	1018.22	1049.42	1008.21	1054.25	871.23	625.4	292.46	582.08
ncRv10884c	154.22	243.62	3599.23	2438.41	129.18	335.72	64.5	84.18	184.33	320.07	340.43	1223.78	772.65	727.79	1958.82
ncRv10887cA	219.49	188.14	44.77	26.82	171.28	100.49	120.46	179.19	239.41	386.27	256.86	466.99	184.21	1043.16	4099.85
ncRv10887cB	211	266.62	63.08	35.86	234.16	111.15	69.24	158.15	246.86	403.81	259.62	413.46	136.45	359.85	728.86
ncRv10922	1099.14	815.26	644.49	524.97	843.6	415.35	961.14	1740.44	1708.83	1082.88	872.2	733.9	768.9	421.31	249.03
ncRv10932c	481.89	1430.51	1136.78	612.82	1398.63	567.91	170.73	285.55	312.73	344.01	410.89	289.18	181.93	2576.9	744.05
ncRv10950c	908.35	739.03	331.67	266.92	501.73	91.35	139.75	428.64	552.35	513.37	426.76	1149.95	293.37	3610.62	53.15
ncRv10953c	551.63	1926.17	965.51	678.2	1485.45	451.44	248.19	768.77	658.29	760.3	643.37	263.95	127.92	1285.75	121.48
ncRv11005c	520.34	696	445.17	402.45	596.71	338.52	306.89	669.53	687.88	747.39	642.75	1141.95	402.53	1655.04	470.72
ncRv11012	938.3	316.73	120.73	497.66	539.09	156.06	549.81	425.55	325.9	368.4	344.42	1437.9	927.87	454.87	22.78
ncRv11015c	382.21	989.29	470.04	520.73	498.57	675.26	306.05	929.7	1014.32	1154.18	1023.73	395.01	126.22	272.92	30.37
ncRv11026	236.03	982.9	239.43	249.77	906.37	711.04	256.73	258.42	349.88	382.3	372.69	116.29	54.58	254.72	45.55
ncRv11051c	400.53	124.24	73.25	81.7	132.08	79.17	174.52	196.53	259.17	222.1	151.78	249.19	86.99	143.54	7.59
ncRv11052A	409.47	356.09	205.85	122.23	366.23	216.2	1059.47	1604.39	1179.86	1105.86	942.93	625.73	416.18	956.23	189.81
ncRv11052B	331.24	316.73	171.94	96.04	337.03	194.89	883.68	1272.93	900.93	790.09	656.58	313.79	250.73	337.61	37.96
ncRv11052C	379.08	549.61	319.8	174.62	518.04	315.93	939.96	1587.06	1252.43	1086.66	791.15	594.36	308.72	950.16	113.88
ncRv11052D	326.1	178.94	107	65.17	159.31	97.82	728.76	1147.74	917.13	758.64	548.58	383.01	179.09	276.96	22.78
ncRv11064c	820.29	1218.59	3231.27	5810.39	1101.59	3323.75	552.03	2283.72	2474.73	2758.52	2702.2	660.81	658.38	351.76	182.22
ncRv11071c	842.73	2044.33	2019.2	2905.76	2320.74	924.5	3058.08	1853.34	1987.69	2501.13	2138.66	1791.19	2422	3063.98	1029.52
ncRv11072	4153.15	3540.99	3683.9	3992.29	4188.87	1437.8	6894.94	4880.22	5340.38	6863.08	6670.86	2858.57	3485.18	9353.39	8255.39
ncRv11075c	250.33	201.44	35.61	10.91	408.59	59.38	26.56	24.14	198.26	441.22	362.55	300.87	141.57	2185.38	22.78
ncRv11077	865.21	1442.27	643.68	519.49	1175.39	472.75	387.62	1072.69	1089.96	973.79	735.69	314.71	102.34	463.96	356.84
ncRv11080c	717.92	954.18	556.97	431.14	863.4	325.83	430.51	869.56	942.51	1135.53	1154.01	1725.23	631.09	1616.63	177.15

Table S4d. Context dependent expression of sRNAs

ncRv11094	1675.59	3637.28	2120.83	2052.59	2022.52	639.98	441.68	2105.45	2419.77	2882.09	2824.38	914.91	1745.43	1342.36	50.62
ncRv11101c	754.58	474.19	183.47	152.48	797.19	153.78	851.75	862.54	1012.7	1020.46	1169.06	917.38	649.85	982.51	68.33
ncRv11133c	3190.85	1682.16	1361.24	1378.63	1665.97	436.65	1436.43	3110.27	3260.34	3292.51	3367.96	2905.33	1797.25	4197.18	174.62
ncRv11147A	123.12	203.41	229.06	303.89	208	211.85	626.73	395.26	306.28	272.08	238.33	135.54	101.36	388.73	522.79
ncRv11147B	170.76	205.53	255.3	375.3	233.84	291.11	1187.39	574.78	559.93	563.35	565.33	355.88	253.12	1151.52	2298.96
ncRv11147C	148.86	130.5	169.4	231.06	184.96	302.99	142.12	179.81	259.01	199.76	146.09	180.89	162.89	236.53	337.86
ncRv11156A	1553.85	744.65	540.24	511.69	919.53	197.17	1475.23	2273.81	1858.89	1856.55	1799.22	1351.14	924.45	402.3	15.18
ncRv11156B	1374.15	744.91	473.43	380.42	885.32	155.3	1142.62	1848.89	1486.01	1463.33	1397.34	919.22	695.9	1287.78	53.15
ncRv11160	471.66	671.51	1025.84	1290.99	556.25	939.76	1164.75	675.17	688.09	764.31	843.35	589.43	886.17	903.89	941.45
ncRv11169c	67.5	151.97	617.22	629.4	165.23	316.31	1021.37	311.81	343.88	349.03	265.77	490.99	411.91	107.15	132.87
ncRv11172c	754.24	885.95	480.21	678.28	841.65	588.09	267.56	493.87	537.69	661.83	656.96	619.74	301.9	3319	1996.78
ncRv11173	2529.26	2586.26	3059.53	1781.85	2796.42	1433.64	2721.81	3543.77	3348.84	3207.21	3767.91	501.33	563.54	394.62	63.78
ncRv11174c	421.99	936.5	1073.53	667.13	911.11	561.44	501.6	914.54	944.18	972.3	911.28	1733.23	1019.97	570.1	129.07
ncRv11176c	298.61	1107.14	556.18	516.21	977.54	322.4	304.47	934.04	807.8	958.4	967.51	761.4	480.14	5909.21	318.88
ncRv11179cA	61.47	23.01	57.31	13.56	38.94	33.88	10.43	147.78	161.01	226.57	284.35	162.43	69.08	522.59	736.46
ncRv11179cB	94.99	355.33	489.54	204.86	329.79	705.33	101.01	424	454.35	500.8	526.31	169.82	142.42	192.05	94.9
ncRv11184c	266.65	1139.73	1938.83	471.16	1452.17	1576.23	98.64	467.02	423.09	480.77	531.07	197.5	165.45	376.02	170.83
ncRv11194c	1192.12	769.96	1371.73	237.73	565.35	168.09	337.92	423.13	379.42	468.56	488.76	705.11	82.55	3659.95	78.96
ncRv11199c	406.97	500.12	737.55	492.05	535.25	320.19	548.17	690.47	775.76	826.23	821.81	454.07	339.76	506.21	396.32
ncRv11221	332.59	584.5	1009.26	1417.05	1084.36	446.11	3794.3	593.44	758.72	896.67	769.49	644.19	1361.95	235.52	242.95
ncRv11248c	183.28	262.92	151.76	143.75	243.63	104.3	286.76	154.13	161.01	155.57	139.49	335.02	298.49	1527.34	687.11
ncRv11268c	141.71	201.18	259.44	144.99	259.68	468.95	38.26	96.25	101.4	104.26	85.41	123.67	47.76	46.5	15.18
ncRv11296	654.95	1784.3	1292.68	628.27	987.82	466.77	2958.77	2596.43	2653.79	2917.68	3526.1	1041.05	428.36	1574.56	167.03
ncRv11297	1968.24	3153.2	2926.56	1131.27	2201.87	743.01	3812.8	5237.17	5066.9	4849.08	4968.74	1471.12	1070.29	27851.93	7531.58
ncRv11302	398.89	403.73	221.79	183.76	686.25	149.46	507.13	120.8	127.42	136.15	127.4	770.32	606.64	318.07	22.78
ncRv11323	799.73	743.12	782.38	727.78	732.73	255.79	517.88	811.17	779.77	770.23	770.87	315.64	245.61	741.94	189.81
ncRv11327c	6.26	165.9	83.09	48.02	215.48	47.2	37.62	28.78	23.65	21.85	12.6	40.61	29	3596.47	478.32
ncRv11351	334.37	410.8	318.79	321.48	583.55	599.89	240.6	228.09	216.08	195.95	144.1	592.51	590.15	165.77	113.88
ncRv11368	229.77	836.93	563.98	273.78	585.92	553.45	195.71	185.69	173.64	145.31	143.18	108.9	47.76	70.76	45.55
ncRv11374c	300.18	205.91	77.92	147.72	202.59	787.92	2504.9	1059.84	1251.05	2808.99	2985.33	922.91	5977.4	139.49	15.18
ncRv11385	352.93	840.38	914.14	661.68	1310.23	304.13	158.56	474.76	466.02	499.64	503.11	231.65	112.57	1352.47	383.41

ncRv11387	739.67	503.42	946.56	410.56	614.89	128.06	57.82	851.44	976.56	1274.41	1099.59	263.34	41.69	271.35	33.74
ncRv11388	12553.31	1690.23	978.57	854.53	1487.16	312.51	722.28	5942.34	6163.67	6051.75	6295.41	2872.1	1477.93	317.4	3.8
ncRv11396c	314.82	748.04	597.05	839.1	722.73	528.14	392.91	345.16	542.39	691.28	724.1	375.63	429.39	360.35	74.03
ncRv11405c	186.86	39.11	13.9	75.46	42.36	157.58	3164.19	2120	4915.78	9122.56	8473.15	886	5464.86	12.13	7.59
ncRv11435c	677.24	820.06	162.45	32.74	752.99	110.39	34.78	177.34	1570.24	2015.43	1958.37	1052.12	450.29	30754.98	607.39
ncRv11440	734.46	918.74	783.74	480.82	1161.58	615.11	296.25	692.95	705.59	732.49	653.51	326.71	173.97	224.4	30.37
ncRv11478	529.28	747.97	351.34	272.53	719.05	127.13	240.92	361.79	763.9	841.72	811.12	496.53	276.31	9408.64	1784.2
ncRv11490A	200.49	253.97	128.02	111.16	258.1	99.35	209.78	209.37	225.15	206.21	225.52	95.98	89.55	226.42	193.6
ncRv11490B	173	347.66	153.63	125.66	299.93	63.19	459.39	328.68	368.99	330.33	323.83	123.67	81.87	1342.36	60.74
ncRv11501	245.86	458.35	297.42	285	562.77	166.72	120.78	372.01	348.91	315.11	334.9	520.52	250.73	1221.06	83.52
ncRv11510	215.47	332.83	189.24	167.45	352.29	92.11	127.1	190.03	249.77	234.01	192.64	280.57	136.45	7156.55	873.12
ncRv11534	1460.28	1071.8	759.55	664.9	709.28	2020.94	4645.67	1340.19	2059.52	3030.74	3767.11	4212.99	1902.92	2020.5	4145.41
ncRv11535	1321.58	872.06	678.4	1462.11	985.57	3411.14	934.65	545.01	663.73	851.19	907.04	1503.24	1063.63	2454.66	7835.28
ncRv11536	146.62	111.97	70.2	60.8	123.66	137.79	245.98	372.31	276.66	282.34	343.19	33.22	29	226.42	926.26
ncRv11591A	535.53	578.75	591.11	1138.44	833.23	299.94	457.49	354.67	264.68	256.52	229.2	537.14	368.42	582.23	37.96
ncRv11591B	623.15	520.97	440.54	949.79	722.2	228.38	497.33	377.89	293.83	297.9	251.32	651.58	412.76	578.18	7.59
ncRv11592c	230.44	263.04	338.71	829.82	396.49	120.28	320.35	168.36	170.32	166.74	175.36	700.49	432.81	530.68	9.49
ncRv11614A	669.19	732.38	316.75	141.56	491.73	80.7	250.4	1219.08	1456.53	1327.62	1290.73	228.88	52.87	171.84	0
ncRv11614B	266.87	1467.83	462.92	196.44	954.78	268.73	80.94	626.4	617.47	562.69	482.06	110.75	32.41	88.95	68.33
ncRv11629	877.06	4381.5	2095.17	1849.38	4881.26	2315.05	651.3	1461.71	1613.97	1856.22	1805.67	1203.48	462.23	1572.82	546.65
ncRv11631	569.95	1290.68	737.28	272.53	845.33	200.22	315.22	1191.22	996.5	967.83	1168.75	70.14	32.41	254.72	15.18
ncRv11640c	1395.1	1388.37	1004.42	1804.62	1366.61	785.21	4420.81	1660.1	2073.53	1938.21	2161.59	2468.13	2338.67	1284.02	223.43
ncRv11645c	445.24	1164.65	1379.94	655.13	1035.29	1182.27	190.33	388.1	436.37	641.14	808.05	278.72	382.06	1718.38	880.71
ncRv11698	439.87	796.93	705.91	634.55	862.7	456.39	239.81	425.08	663.63	717.6	686.69	446.69	177.39	314.36	68.33
ncRv11702cA	266.87	653.9	243.5	172.12	489.63	207.83	1150.21	1178.84	963.13	823.52	723.86	265.8	153.51	95.02	30.37
ncRv11702cB	285.65	758.2	292.33	244.46	682.21	238.28	988.97	782.08	651.81	585.53	485.44	260.26	156.92	90.97	22.78
ncRv11706A	82.85	445.31	1041.82	636.6	45.43	447.63	771.87	217.51	360.03	423.67	474.59	169.08	154.87	794.9	850.34
ncRv11706cA	65.52	27.78	26.45	162.35	28.41	257.31	1561.23	385	290.36	257.7	264.49	556.21	676.57	233.16	242.95
ncRv11706cB	154.31	21.4	19.48	98.71	334.03	178.79	309.46	367.58	253.21	309.68	337.84	890.74	950.77	108.59	315.84
ncRv11733c	503.57	33.62	72.41	447.3	35.26	846.54	6812.26	872.14	479.79	712.3	1237.88	1775.68	1101.84	3567.16	158933.89
ncRv11734c	53.64	24.8	50.87	460.55	10.79	924.19	971.89	169.6	96.54	100.95	101.39	286.1	336.01	145.56	75.92

ncRv11737cA	195.8	68.76	22.72	896.78	74.72	292.33	675.65	136.17	122.46	331.66	376.07	1173.94	109.16	2318.8	470.72
ncRv11737cB	405	108.9	41.15	889.3	132.6	524.27	2404.76	150.41	135.42	901.85	1196	11733.3	822.12	9817.01	1791.79
ncRv11759cA	50.96	67.49	68.51	54.88	37.36	45.68	732.56	223.14	145.46	149.61	141.95	47.99	17.06	10.11	7.59
ncRv11759cB	40.83	100.21	96.99	62.78	55.25	126.37	546.12	142.16	90.71	92.46	92.99	65.22	45.48	214.29	217.65
ncRv11765c	685.73	191.21	39.57	34.3	142.6	44.15	855.33	1162.03	1350.05	1389.52	1275.67	655.88	252.43	606.49	70.86
ncRv11778c	319.62	174.85	384.58	368.88	377.02	130.94	181.8	581.22	588.96	588.18	411.4	745.71	532.16	2035.78	394.8
ncRv11781cA	166.74	355.58	312.34	541.94	349.39	272.54	754.69	372.01	298.37	302.2	226.44	586.97	876.7	236.53	113.88
ncRv11781cB	559.67	188.66	210.94	374.49	203.11	105.06	1683.58	926.3	1001.36	926.13	770.26	1714.77	2437.35	1799.25	933.86
ncRv11790	162.49	271.61	205.35	248.21	301.9	313.65	176.74	640.49	575.84	623.26	569.94	1209.02	784.59	443.75	451.74
ncRv11794	352.06	507.68	429.72	364.2	488.08	268.48	642.31	1288.3	1017.31	977.76	827.44	1002.9	681.88	1064.27	467.35
ncRv11803c	51.85	13.8	10.51	39.29	22.89	34.26	3175.25	337.34	230.98	255.2	190.18	985.67	1785.8	105.12	1700.68
ncRv11804c	674.11	361.36	526.88	1039.35	416.22	1158.06	786.62	558.44	630.04	859	1215.21	1277.31	783.91	1063.37	619.53
ncRv11806	46.94	6.14	6.1	89.49	4.74	184.99	72.72	65.3	48.27	53.29	48.85	472.53	307.01	16.17	926.26
ncRv11806B	24.74	3.92	8.59	64.44	3.33	199.96	73.35	64.37	50.54	47	44.45	662.04	347.95	105.12	4337.75
ncRv11808	269.56	31.7	52.23	145.93	61.56	252.74	90.42	113.27	138.98	140.01	110.61	500.22	479.28	268.88	926.26
ncRv11809A	930.26	141.87	195.34	193.64	261.52	711.04	414.81	717.7	886.36	746.73	1043.09	1380.68	1268.99	412.41	2308.07
ncRv11809B	729.1	159.51	180.42	173.68	240.6	613.59	309.37	655.81	817.51	692.61	1052.31	1136.11	1021.68	247.65	596
ncRv11810	770.22	424.47	775.6	625.5	717.21	714.84	227.01	388.72	406.89	367.4	463.94	426.39	309.57	248.66	68.33
ncRv11812c	197.58	182.29	109.65	210.3	167.27	208.59	3135.87	452.68	191.5	194.33	202.23	1908.17	530.64	752.94	32.06
ncRv11813c	320.45	132.56	61.63	194.84	225.06	725.17	6154.12	870.46	358.39	454.69	479.74	4269.66	1189.07	2484.29	188.72
ncRv11814	110.86	690.88	78.91	93.55	616.18	142.61	204.88	61.9	275.15	265.9	312.36	146.44	65.95	265.51	30.37
ncRv11818c	922.66	644.19	401.87	513.87	520.93	179.66	341.78	497.35	546.85	539.85	568.71	334.09	194.44	248.66	60.74
ncRv11826	1236.72	1300.14	812.27	677.09	1382.69	613.16	1810.64	1702.72	1559.92	1621.59	1511.46	2163.31	1267.53	7362.18	3989.22
ncRv11828	354.34	598	656.56	538.82	496.55	378.61	932.06	531.7	372.77	430.51	444.07	360.55	205.81	2029.71	2186.59
ncRv11829A	308	437.89	477.84	370.75	499.36	470.47	300.36	263.68	238.43	253.54	228.59	155.05	185.91	72.78	204.99
ncRv11829B	315.15	395.97	425.61	372	465.16	468.19	683.55	397.07	298.69	304.52	288.19	123.67	129.63	46.5	53.15
ncRv11829C	264.19	287.33	288.94	302.46	253.89	233.71	1155.9	637.24	458.73	422.68	433.21	215.96	182.5	133.43	75.92
ncRv11829D	376.1	831.82	515.71	441.32	805.26	449.16	504.39	604.53	890.24	918.4	814.6	936.45	347.95	1103.81	496.03
ncRv11832	91.91	251.85	604.61	849.26	257.2	153.47	224.6	178.39	162.11	122.6	99.18	144.71	202.63	129.38	30.37
ncRv11846c	103.49	126.28	2278.47	1459.93	117.74	132.84	339.56	202.72	162.47	152.92	140.87	96.91	715.51	232.49	246.75
ncRv11856c	173.89	825.56	872.59	655.44	882.04	227.62	46.63	258.27	262.41	329.67	333.97	63.68	24.73	2534.11	349.25

ncRv11875A	253.02	198.62	2764.62	2902.39	403.33	120.28	3793.36	496.73	230.01	210.51	326.91	247.34	1144.48	173.86	212.59
ncRv11875B	66.61	125.09	1782.38	1989.6	287.92	77.9	1618.77	211.79	79.69	81.65	153.01	100.91	656.67	37.74	10.12
ncRv11875C	14.75	25.05	158.04	281.57	31.31	24.36	1895.73	121.32	57.02	49.65	36.56	51.68	172.27	36.39	60.74
ncRv11886c	293.25	624.42	334.16	327.41	502.87	334.96	770.18	1860.03	1642.91	1746.11	1482.55	1337.61	344.54	1952.89	339.12
ncRv11903	311.8	960.4	1167.64	402.09	727.6	1320.06	293.4	456.34	589.93	775.52	1078.88	683.88	263.52	601.43	645.35
ncRv11914c	85.38	329.25	309.63	265.67	357.81	392.82	547.28	247.59	218.03	170.13	158.54	254.72	220.03	367.94	447.95
ncRv11941	199.82	310.59	147.52	120.36	346.5	104.3	235.23	284.11	232.93	261.82	284.51	271.34	175.68	2298.59	151.85
ncRv11976cA	5.81	171.78	163.12	125.35	123.66	127.13	58.17	24.45	22.35	28.8	29.5	59.07	122.81	489.23	554.24
ncRv11976cB	61.24	93.05	91.11	88.66	87.87	114.19	318.17	152.27	112.95	119.16	141.33	337.17	484.4	388.15	463.13
ncRv11979c	507.46	1057.7	702.14	452.13	835.18	677.84	466.79	877.59	920.57	814.65	543.45	857.94	214.23	414.03	78.96
ncRv11980c	379.43	914.95	664.57	601.43	661.22	588.32	357.14	582.71	764.94	732.43	588.06	639.39	220.37	951.78	215.62
ncRv11984c	221.28	128.58	205.18	175.24	185.75	86.02	957.98	451.85	483.03	450.48	329.36	252.88	250.73	113.21	30.37
ncRv11985c	19.67	56.24	48.16	93.23	54.99	130.18	137.53	52.92	47.95	24.82	34.41	35.07	59.7	572.12	850.34
ncRv11986A	53.2	115.29	776.96	1922.97	115.24	35.02	404.06	161.24	185.63	128.43	102.31	101.52	325.78	137.47	53.15
ncRv11986B	39.34	55.98	434.43	930.15	53.41	22.84	341.46	146.7	145.78	93.01	78.04	59.07	155.21	72.78	30.37
ncRv11987	589.18	663.62	214.67	178.67	775.61	267.21	274.43	186.31	444.8	513.7	413.55	457.76	325.78	2288.48	569.42
ncRv11988	50.07	73.79	244.4	633.61	115.06	741.99	480.57	94.5	54.43	48.55	54.28	289.18	309.29	67.39	415.05
ncRv11990c	22.35	119.89	95.64	154.66	209.43	215.44	138.8	74.9	64.79	52.3	42.71	145.82	143.27	260.79	759.23
ncRv11996A	132.32	26.24	31.43	430.31	45.43	111.15	3006.1	60.87	43.41	151.15	225.52	1877.82	152.37	226.42	20.25
ncRv11996B	131.87	30.93	23.06	408.17	17.63	156.06	2638.09	59.11	37.26	133.06	186.5	2408.8	170.56	1077.53	888.3
ncRv12031c	14.3	111.2	25.1	2847.82	82.35	853.39	95.17	14.55	20.09	22.51	12.9	71.99	46.05	1047.2	220.18
ncRv12049cA	896.73	298.83	250.96	294.35	229.68	71.56	603.56	362.72	412.08	367.74	345.65	273.18	163.74	117.25	7.59
ncRv12049cB	422.88	824.41	617.56	964.14	759.56	433.93	1008.25	418.43	406.89	379.65	361.63	860.15	972.21	766.2	835.16
ncRv12075c	132.32	102.25	81.05	91.36	121.81	66.99	798.32	383.15	294.16	272.08	310.01	94.14	61.4	345.7	113.88
ncRv12080	553.24	515.35	406.83	346.74	607.02	293.85	229.03	434.89	458.6	412.95	348.17	183.11	95.52	360.66	127.55
ncRv12081c	539.33	514.97	550.08	343	501.99	496.35	494.33	549.34	569.04	619.46	512.94	367.32	156.07	370.97	265.73
ncRv12108	293.92	621.82	800.86	584.19	721.41	298.8	478.04	263.22	228.39	234.51	213.69	80.29	47.76	123.32	18.98
ncRv12111c	774.69	1732.66	1600.71	1501.4	1747.23	922.67	936.8	1038.64	956.66	1006.23	999.77	636.81	399.12	1742.64	326.47
ncRv12115c	44.26	311.87	569.75	537.26	514.09	489.5	213.73	49.21	34.66	42.37	50.7	184.58	467.34	240.57	933.86
ncRv12126c	278.05	997.64	461.45	449.43	962.41	489.25	293.82	382.73	389.18	448.17	396.55	455.3	176.25	3311.42	602.32
ncRv12145cA	385.33	588.08	318.45	248.05	577.37	192.22	1321.73	830.05	938.68	1045.78	1096.24	1615.1	992.68	1887.19	269.53

Table S4d. Context dependent expression of sRNAs

ncRv12145cB	250.33	593.57	513.9	459.41	519.53	273.55	1184.57	670.15	546.41	560.49	540.34	530.37	577.64	1454.22	779.48
ncRv12146c	1152.43	1489.69	928.72	900.84	1689.22	535.18	205.03	311.66	630.59	638.99	706.81	584.2	203.82	2252.09	125.27
ncRv12161c	4393.79	3706.64	5434.62	3617.39	1852.47	2336.37	1025.01	4645.73	5380.66	4630.63	3922.88	1810.75	1296.28	2153.03	3059.71
ncRv12169c	246.46	321.58	228.8	450.47	401.49	505.49	445.16	166.92	183.79	186.02	142.36	647.27	1100.7	490.58	804.79
ncRv12190c	462.67	828.24	426.46	173.84	555.27	238.28	197.29	865.48	852.02	735.64	681.31	721.72	186.77	1191.75	83.52
ncRv12198c	532.63	770.47	404.25	341.28	967.28	350.95	375.76	595.3	699.11	812.43	715.42	1030.89	312.13	3988.67	508.69
ncRv12200c	277.45	1262.64	692.29	502.02	1968.32	368.97	243.03	442.78	530.43	578.14	552.42	1091.5	469.62	469.02	20.25
ncRv12202c	183.73	427.16	364.23	306.52	264.15	136.27	2615.64	1442.84	954.06	826.83	886.09	415.31	322.37	2047.91	387.21
ncRv12214c	708.98	394.69	238.75	299.97	406.49	124.09	337.03	761.96	670.92	688.14	663.34	1234.86	709.54	1148.28	53.15
ncRv12219A	845.77	672.31	420.19	378.86	970.04	336.49	500.49	573.17	674.81	716.6	511.56	1192.4	257.55	673.2	106.29
ncRv12220	1353.92	975.61	591.79	372.54	1842.34	560.87	247.48	539.28	607.18	622.19	574.7	646.04	164.59	745.47	275.22
ncRv12225	80.46	75.92	84.44	113.19	47.62	54.05	947.55	134.63	125.7	109.89	183.12	108.9	73.34	18.19	15.18
ncRv12301	945.01	391.63	322.18	309.01	520.67	355.52	626.96	638.17	631.07	641.14	583.76	1962.11	1555.54	853.13	789.6
ncRv12307c	48.28	350.72	209.58	133.46	393.59	149.21	116.35	64.68	70.3	101.28	108.46	99.67	146.68	1024.96	394.8
ncRv12327A	276.26	739.54	814.26	459.31	538.82	277.11	337.66	471.35	380.01	454.13	535.83	247.34	233.67	224.4	463.13
ncRv12327B	169.87	215.5	218.74	125.66	128.65	76.89	323.75	519.01	403.33	494.18	580.38	245.49	240.49	222.38	652.94
ncRv12329c	73.31	59.56	35.61	10.91	103.13	44.92	79.67	259.04	396.85	249.9	160.38	895.23	225.14	52.56	60.74
ncRv12333cA	356.72	1349.73	778.65	697.53	2007.44	394.34	152.39	254.71	364.13	397.53	348.72	245.49	267.78	200.14	273.32
ncRv12333cB	574.87	804.55	495.36	420.33	1171.14	212.14	256.94	804.46	1038.19	1203.72	1090.1	510.68	492.36	757.44	25.31
ncRv12357c	178.36	95.35	212.64	362.33	324.66	149.21	2247.31	536.34	474.28	428.97	460.25	411.62	677.14	151.62	91.11
ncRv12359	298.16	206.8	236.38	239.79	376.23	126.37	1261.18	470.11	484.97	424.67	413.55	191.97	329.19	169.82	204.99
ncRv12374c	710.54	472.02	506.5	319.77	636.96	251.6	123.46	1014.5	804.23	809.28	763.5	653.42	194.44	702.51	189.81
ncRv12386cA	346.44	261	282.84	259.74	277.83	200.98	385.09	1018.22	1085.27	1403.42	1311.93	365.47	416.18	232.49	83.52
ncRv12386cB	158.25	343.57	452.4	408.06	443.76	380.64	369.7	391.81	358.95	452.36	367.87	187.04	362.73	350.42	465.66
ncRv12390cA	567.5	1604.21	742.03	802.46	2306.71	674.11	2065.04	376.18	652.78	600.26	822.34	465.15	892.9	588.29	265.73
ncRv12390cB	1132.76	1007.8	554.82	627.75	1485.45	347.14	5423.89	925.37	1549.44	1429.11	1914.86	1091.25	2075.42	442.33	167.03
ncRv12395	5364.58	5292.06	5237.59	12239.84	3730.91	7016.46	2360.28	2087.4	2353.46	2732.48	3638.99	1544.34	3805.84	16148.74	77092.44
ncRv12395A	6873.88	4032.57	4277.16	5274.07	2903.29	3736.36	4842.71	5467.12	5039.85	4888.8	4989.01	5243.99	7590.08	1661.78	4684.46
ncRv12411c	437.19	1776.38	534.48	586.53	1942.19	966.83	450.22	594.84	594.14	565.67	529.69	430.08	139.86	2011.52	318.88
ncRv12442c	728.2	270.71	103.1	66.73	227.58	145.4	1075.91	1247.55	1221.98	1323.65	1101.77	1903.05	692.49	956.23	197.4
ncRv12444c	404.78	397.63	327.43	294.82	413.59	227.24	954.27	617.97	634.23	808.37	1136.42	596.66	486.96	1241.28	964.22

Table S4d. Context dependent expression of sRNAs

ncRv12455c	447.02	379.7	556.63	837.12	465.33	155.3	572.68	556.25	665.85	704.36	554.68	1141.95	826.66	1757.47	339.12
ncRv12457cA	533.45	786.32	661.09	393.51	888.75	296.9	630.65	701.51	1382.45	1598.93	1194.36	1899.97	634.5	10318.37	3872.08
ncRv12457cB	246.31	595.87	268.59	143.75	596.97	156.82	547.6	494.87	824.48	905.6	665.8	985.67	240.49	3602.54	220.18
ncRv12459	1162.48	570.31	652.32	619.42	679.98	330.78	1342.91	910.36	784.31	773.54	666.1	533.44	615.73	1232.18	573.22
ncRv12461c	776.63	717.13	587.61	557.11	1109.92	399.93	380.87	760.93	668.22	657.58	519.45	627.58	227.42	6498.85	3254.58
ncRv12484c	140.81	346.12	182.79	161.21	172.59	218.49	314.9	139.27	165.22	175.43	185.57	448.54	798.24	659.05	1662.72
ncRv12497cA	206.82	71.24	730.95	208.5	63.23	62.17	902.13	517.16	453.87	334.97	253.78	2346.04	4299.34	1452.2	3322.91
ncRv12497cB	139.92	56.75	799.68	155.6	46.31	30.45	487.21	423.69	354.74	262.15	204.93	825.08	1117.19	230.47	83.52
ncRv12512cA	199.37	165.9	208.91	128.78	171.54	135.51	491.64	386.86	388.1	390.91	382.21	500.22	288.25	853.13	812.38
ncRv12512cB	134.33	183.93	76.47	70.31	164.57	110	430.14	436.38	437.02	375.68	378.98	703.26	354.77	530.68	170.83
ncRv12519	410.81	380.63	175.33	151.23	395.17	125.61	450.54	435.14	459.7	414.74	262.39	980.13	414.47	105.12	0
ncRv12524cA	828.48	257.68	80.04	223.05	391.84	197.43	524.2	419.67	717.46	808.73	1127.58	2784.74	2061.55	5113.36	182.22
ncRv12524cB	257.49	285.28	75.97	167.13	265.47	128.66	182.43	152.89	294.16	321.73	453.8	1197.94	788	422.52	15.18
ncRv12542	403.66	592.3	1059.79	464.61	452.53	366.94	1172.97	1479.97	1129	1320.34	1341.73	221.5	144.98	897.6	303.69
ncRv12560	446.58	86.15	21.7	14.97	112.61	14.46	93.59	104.92	177.85	156.89	113.07	265.8	64.81	16.17	0
ncRv12589	180.15	1548.1	8109.03	2840.65	1120.01	3007.82	300.04	1081.35	1274.78	2176.29	3176.28	121.82	419.59	418.48	402.39
ncRv12615cA	4.25	7.29	34.25	107.73	6.58	3.43	55.96	13.77	7.78	8.61	8.3	30.46	469.9	24.26	7.59
ncRv12615cB	15.2	21.22	158.04	411.29	11.31	6.09	106.86	31.26	17.82	12.91	11.37	38.76	1652.76	10.11	22.78
ncRv12617c	71.97	74.39	318.45	536.95	86.56	25.88	285.81	136.17	96.22	93.67	82.03	59.07	788	390.17	75.92
ncRv12622	167.63	48.83	352.36	1570.62	56.83	685.15	3454.43	84.49	62.52	204.89	218.14	4858.21	653.26	3361.96	3462.1
ncRv12626cA	17.08	3.27	1.36	100.09	2.32	4.57	862.75	12.57	5.77	25.55	16.41	593.62	33.43	11.73	4.56
ncRv12626cB	89.85	10.23	5.43	370.75	9.73	11.42	2997.57	30.95	17.17	84.4	52.85	1951.04	68.23	18.19	0
ncRv12628	267.12	87.31	45.75	452.34	110.62	266.62	4654.73	339.13	245.92	538.79	536.04	7581.42	960.46	1058.88	315.5
ncRv12632c	216.81	223.29	274.36	249.3	251.26	622.35	287.87	82.79	113.55	145.97	155.77	1012.44	1632.29	378.04	2402.97
ncRv12637	97.9	258.44	345.41	394.92	235.6	617.78	99.75	124.41	122.78	133.89	154.85	168.89	180.8	82.89	121.48
ncRv12664	272.98	799.78	508.93	619.68	560.05	313.14	1317.57	885.34	905.15	975.77	1072.89	604.2	475.3	2384.17	318.88
ncRv12688c	396.96	201.18	165.16	184.6	153.65	92.11	2114.2	769.7	689.06	860.26	1055.69	590.66	680.55	1548.56	804.79
ncRv12694c	218.59	452.72	351.34	822.88	369.65	379.12	1359.83	288.13	323.96	441.88	396.96	1017.05	1773.86	135.45	182.22
ncRv12702	324.39	735.19	691.72	1163.49	582.32	457.02	1275.2	662.2	726.75	816.68	721.51	608.51	434.94	808.65	374.55
ncRv12706c	1677.23	1008.97	1910	1856.24	2505.75	354	9768.89	2221.51	3114.88	3675.7	3458.02	4088.5	8308.15	1067.42	296.1
ncRv12709	51.41	1133.72	895.31	2030.86	1282.87	780.31	238.39	109.87	59.93	47.99	66.06	110.75	109.16	3145.65	12504.56

Table S4d. Context dependent expression of sRNAs

ncRv12710	715.54	823.81	824.77	1975.88	1393.19	473.52	2306.96	1046.28	1031.92	1146.79	854.55	935.22	1895.53	1088.98	1351.43
ncRv12744c	854.49	389.58	251.3	513.72	347.42	132.46	5851.12	1502.1	1555.66	2076	1915.05	2577.7	5023.1	547.86	573.22
ncRv12783cA	1506.47	534.01	261.13	241.97	606.7	73.84	2044.01	1691.05	2185.11	2133.93	1952.84	891.53	339.42	382.09	15.18
ncRv12783cB	433.84	590.25	255.54	261.93	571.19	123.33	867.4	464.85	514.61	512.55	470.24	239.03	98.93	1986.25	72.13
ncRv12792c	252.12	467.8	237.62	109.34	315.72	107.09	100.54	459.9	330.01	315.11	265.05	183.35	102.34	3207.65	713.68
ncRv12808	208.31	219.08	59.35	29.93	222.32	89.83	222.58	427.09	424.06	442.21	429.83	468.84	322.37	7225.29	1791.79
ncRv12813A	7.15	10.23	3.73	2.18	16.05	3.81	10.12	23.52	16.52	21.18	12.9	29.53	13.65	113.21	447.95
ncRv12813B	13.86	8.95	1.7	2.49	14.73	4.57	26.24	50.14	56.69	63.88	56.84	18.46	20.47	240.57	766.82
ncRv12813C	3.58	8.69	3.39	1.25	11.84	3.05	25.93	32.19	41.14	54.61	38.71	44.3	20.47	236.53	675.72
ncRv12842c	81.81	434.06	305.05	354.69	502.52	199.46	122.67	81.24	84.55	82.42	92.94	95.06	90.4	2555.33	1226.16
ncRv12866	95.22	349.32	564.32	536.32	271.52	251.22	652.09	305.93	204.1	169.97	177.59	249.19	469.9	247.65	231.57
ncRv12890c	1052.74	219.16	107.51	116.31	225.74	87.8	742.36	734.42	887.11	881.66	799.86	1193.63	600.38	129.38	37.96
ncRv12898c	128.3	153.38	109.54	69.85	90.51	44.15	135	423.07	309.71	282.01	253.78	358.09	202.97	2082.27	182.22
ncRv12904cA	564.74	1019.45	552.34	466.06	732.99	425.3	571.42	788.37	844.24	858.6	774.05	921.68	324.07	529.67	35.43
ncRv12904cB	520.51	1425.29	1212.61	616.4	1800.33	500.62	156.19	471.78	674.1	823.38	829.56	661.54	530.11	2550.48	482.87
ncRv12909c	2215.9	295	95.97	125.97	348.6	82.98	452.43	1068.05	1207.72	1151.2	1087.03	2829.65	852.82	875.36	68.33
ncRv12929	523.02	467.38	1246.54	899.23	623.98	292.08	1511.06	2219.76	2509.61	2633.56	2115.83	2177.77	1392.65	1286.09	150.58
ncRv12939	587.21	675.27	560.52	359.84	780.87	317.61	252.3	934.41	787.22	723.95	632.43	474.01	324.07	1105.42	513.24
ncRv12940c	365.49	555.43	555.77	383.16	411.8	273.76	374.97	1017.35	1031.88	862.84	682.33	1779.38	792.78	3082.57	543.61
ncRv12942	198.93	340.5	193.98	111.32	124.45	79.17	549.18	1930.9	1217.12	1055.88	1089.18	127.36	47.76	101.08	30.37
ncRv12950c	271.19	564.94	683.47	199.77	379.74	453.72	651.72	1518.97	1629.09	1993.92	1834.04	1016.43	351.36	2462.34	551.71
ncRv12953	710.32	1026.36	619.26	295.6	1276.55	312.12	377.19	849.55	784.63	818.55	832.32	452.23	184.21	325.48	45.55
ncRv12958c	656.23	396.74	130.91	89.49	649.06	156.82	56.28	594.22	588.31	470.68	296.18	1760.92	409.35	244.62	37.96
ncRv12970c	438.08	694.8	411.2	313.69	570.66	282.82	678.81	596.69	611.96	648.42	628.93	424.54	325.78	555.95	94.9
ncRv12990c	587.77	2906.96	891.24	1814.06	471.25	258.84	130.71	693.7	462.62	932.18	1360.12	170.34	140.35	302.67	344.91
ncRv13000	790.67	228.85	102.84	85.67	184.69	113.62	732	668.11	581.59	544.82	622.01	466.99	270.77	211.26	170.83
ncRv13003c	155.12	563.92	288.94	198.32	595.65	338.01	296.56	416.88	315.54	337.95	317.38	254.72	109.16	22332.89	2839.53
ncRv13027c	1139.46	875.79	763.9	662.77	818.89	135.89	135.48	591.28	620.71	617.97	446.58	298.1	82.72	135.45	3.8
ncRv13034c	398.3	134.46	68.84	100.4	132.6	45.68	275.7	352.82	382.27	349.86	395.11	371.01	218.32	1467.7	189.81
ncRv13053c	578.09	833.93	1768.24	1700.15	1244.87	626.89	3738.98	1405.12	1390.22	1252.27	1396.19	2701.3	3276.87	1177.66	907.03
ncRv13059	175.68	202.2	175.67	115.68	151.02	121.8	237.44	147.01	192.43	193.96	151.78	823.24	745.36	165.77	235.36

Table S4d. Context dependent expression of sRNAs

ncRv13061c	63.92	58.79	50.19	104.46	29.99	45.68	2076.26	244.5	189.84	180.06	194.79	199.35	426.41	204.18	645.35
ncRv13124	224.41	219.33	281.14	134.7	149.18	239.04	494.48	931.56	903.53	1007.22	909.44	363.63	167.15	446.78	409.99
ncRv13130c	201.46	155.76	87.5	1015.9	199.78	5549.73	1531.3	48.49	66.3	282.23	344.11	2274.06	65.95	397.59	693.43
ncRv13134c	657.27	431.93	145.15	170.46	701.77	378.86	721.17	1115.4	1224.03	980.19	553.14	1167.79	689.65	324.81	35.43
ncRv13171c	952.16	2379.92	1008.36	501.82	3632.33	715.6	653.41	1111.68	2017.84	2546.24	2452.82	424.54	258.12	305.94	40.49
ncRv13176c	20.12	65.44	111.58	86.37	41.57	97.44	169.15	101.2	73.22	75.8	59.91	83.06	85.28	272.92	387.21
ncRv13197AA	211.44	177.28	122.6	87.15	160.36	91.73	678.65	648.38	509.27	378.66	352.72	959.83	208.94	89.96	11.39
ncRv13197AB	482.96	192.34	143.25	108.89	154.28	118.15	458.69	376.09	320.72	268.9	281.56	684.43	213.55	264.43	75.92
ncRv13202cA	180.15	74.13	43.07	98.22	88.93	32.74	663.63	195.6	190.16	180.39	164.38	265.8	627.67	90.97	91.11
ncRv13202cB	147.96	82.06	56.47	89.02	114.58	36.54	498.12	160.62	171.86	147.79	131.96	291.64	585.03	41.44	53.15
ncRv13207c	625.61	244	195.85	210.16	228.37	80.7	354.26	472.74	529.35	618.63	657.96	210.42	128.78	170.83	15.18
ncRv13208A	316.05	524.3	138.37	73.59	605.39	91.35	36.36	93.78	511.86	529.92	495.28	291.64	129.63	2403.71	7.59
ncRv13210c	692.71	692.25	436.53	454.75	619.96	197.63	630.31	1090.76	1525.21	1719.72	2307.4	823.24	555.36	3013.03	534.5
ncRv13218	1571.86	1847.62	2958.51	2123.29	2564	1114.3	950.57	2346.63	2681.28	3034.85	2985.26	3944.27	3607.66	8789.45	3587.91
ncRv13221c	1420.55	1074.06	728.53	480.82	1058.02	550.71	1392.2	1034.93	1033.5	1102.08	1178.95	787.43	722.85	4506.2	1072.04
ncRv13223c	139.98	192.23	164.72	183.17	180.79	127.68	7235.95	683.97	520.37	450.44	315.76	1369.08	2018.01	166.35	132.32
ncRv13241c	396.06	324.36	464.13	453.47	416.37	433.71	526.01	348.13	449.47	512.76	462.62	742.55	1211.49	218.91	240.79
ncRv13260c	598.12	753.19	698.48	578.17	927.32	171.14	57.16	366.74	903.01	1043.43	878.84	942.48	524.65	562.01	56.18
ncRv13287c	137.09	88.96	236.49	351.31	92.26	154.29	2945.82	463.82	232.6	199.92	219.78	1109.96	2752.9	22.91	161.97
ncRv13303c	729.99	479.56	133.96	67.35	184.69	93.64	287.08	1218.15	1120.26	934.4	859.97	84.91	20.47	62.67	0
ncRv13332A	982.11	168.72	127.51	53.94	66.3	23.6	63.23	211.38	210.25	292.93	281.13	38.76	22.17	84.91	7.59
ncRv13332B	942.33	154.66	127.18	43.34	56.83	12.94	75.88	232.74	226.77	324.71	326.29	40.61	15.35	70.76	30.37
ncRv13339c	400.35	173.42	248.25	475.58	200.59	644.96	2201.9	648.32	685.37	757.19	703.34	736.85	1137.32	157.69	30.37
ncRv13401	415.51	214.09	1051.82	805.73	176.01	69.28	56.28	154.28	122.94	125.12	124.74	56.3	62.26	34.37	3.8
ncRv13402cA	113.54	162.58	3346.57	3037.09	318.35	91.35	225.43	239.54	173.32	183.37	177.59	197.5	492.93	147.58	53.15
ncRv13402cB	63.73	120.73	845.61	631.92	229.05	53.72	95.84	125.48	95.43	87	68.3	44.3	130.6	41.01	4.34
ncRv13411c	350.91	973.44	1178.49	938.26	1015.3	436.21	83.47	269.26	333.68	382.63	371.76	166.12	57.99	1099.76	296.1
ncRv13415c	115.78	244.64	982.64	1718.58	323.22	333.06	2609.95	1381.87	1083	1389.85	1298.41	347.94	717.22	129.38	136.66
ncRv13418c	7709.88	518.78	511.12	1501.71	1364.28	1554.75	3457.05	4414.55	3263.3	2111.18	2099	17971.48	8809.37	2860.31	468.55
ncRv13424c	161.82	151.59	62.74	59.87	152.07	159.11	650.99	1359.27	1135.48	1155.5	781.01	105.21	57.99	99.06	0
ncRv13425	145.73	106.85	50.53	66.42	121.81	79.93	150.49	1961.54	1923.35	2267.32	1295.03	145.82	75.05	525.62	7.59

Table S4d. Context dependent expression of sRNAs

ncRv13443c	2819.53	1548.61	407.19	550.88	1437.57	440.53	687.98	1105.7	1293.03	1400.33	1214.02	607.89	262.67	393.54	15.18
ncRv13460c	2607.55	2366.92	1088.23	859.28	2730.81	762.8	2495.99	1969.32	2548.18	2590.84	2267.98	2794.05	1361.1	9244.03	1943.63
ncRv13462c	2019.95	2637.76	1077.99	1134.29	2910.74	990.93	1882.98	1226.09	1676.28	1765.86	1708.07	7720.47	5140.22	7555.49	2725.64
ncRv13476c	922.81	1251.82	585.68	149.05	782.54	781.07	76.83	957.66	1527.69	2073.58	2059.25	2302.97	490.65	1562.04	794.66
ncRv13478	1931.32	1993.51	2083.91	668.29	1024.29	1541.44	243.07	808.01	1285.86	1487.89	1368.09	1457.46	474.85	1011.62	1263.36
ncRv13483c	215.24	328.23	497.34	543.18	336.77	194.13	331.82	358.39	457.59	493.18	415.09	229.81	156.92	244.62	37.96
ncRv13487c	274.38	737.24	685.12	179.54	577.04	382.16	171.73	1019.98	1161.35	1270.71	1397.47	719.09	648.68	1023.16	1181.21
ncRv13490	97.45	913.62	904.58	519.49	684.76	186.26	55.43	131.22	77.1	93.78	119.62	28.3	28.43	328.85	5.06
ncRv13503c	79.12	47.04	61.38	105.08	97.08	52.53	77.14	49.21	52.48	58.92	55	263.95	201.27	371.98	766.82
ncRv13520c	421.99	232.37	225.69	202.84	206.8	132.08	1089.51	1443.45	1214.69	1198.2	1035.87	650.65	369.27	1548.56	284.71
ncRv13555c	571.74	199.39	155.66	295.6	169.17	102.01	993.39	415.64	314.24	287.3	313.7	223.34	191.03	1419.18	258.14
ncRv13573c	67.23	78.73	63.49	121.61	91.35	50.24	412.41	87.15	103.28	165.76	199.09	612.81	777.09	300.82	279.4
ncRv13595c	1129.18	770.81	859.82	957.28	552.86	243.1	905.08	770.42	632.37	550.78	514.94	244.88	358.18	1074.16	526.4
ncRv13596c	737.14	405.86	336.99	267.95	317.65	111.91	592.71	469.29	516.28	521.87	534.4	1087.81	984.72	1014.18	184.75
ncRv13614c	1632.83	1729.25	5172.47	4735.04	1206.74	11940.93	555.61	2538.01	4001.99	5678.56	6167.6	5418.11	7554.83	1870.68	16450.03
ncRv13616cA	174.79	385.75	1270.74	935.76	190.48	2227.51	294.98	617.74	682.26	1158.81	1431.45	2106.09	2558.45	2136.86	4692.06
ncRv13616cB	58.11	109.67	183.81	186.15	57.36	227.62	190.33	259.35	185.31	203.23	234.12	354.4	366.71	333.57	850.34
ncRv13632	869.24	625.4	483.27	485.03	721.28	1454.43	269.53	218.65	249.29	277.04	288.5	610.97	544.95	1602.14	8480.62
ncRv13646c	1382.87	1843.99	728.63	682.25	1696.98	362.75	394.1	795.39	790.3	865.39	662.88	731.87	302.75	2550.28	83.52
ncRv13647c	2244.51	4588.44	2126.88	785.78	4502	1079.5	147.65	1557.66	1798.63	2621.48	2519.7	367.32	119.39	316.38	60.74
ncRv13648c	7558.91	4273.19	2003.31	819.72	3694.12	925.28	464.31	4648.43	5662.55	7861.71	7643.95	3538.71	1508.76	6171.16	516.28
ncRv13660c	206.45	1034.58	1421.2	775.02	845.25	652.92	500.54	647.86	559.32	685.88	740.35	110.44	45.2	3746.75	4560.46
ncRv13661	24085.59	69663.23	167820.22	155625.89	84794.14	928678.08	39945.39	15674.04	21286.03	33216.31	40914.66	168904.9	413577.29	12844	980781.52
ncRv13680	361.79	600.39	629.43	653.46	567.59	374.8	583.54	358.49	387.46	429.41	392.14	158.13	143.27	173.86	118.95
ncRv13681cA	234.69	545	652.15	753.04	465.68	319.74	536.85	456.19	338.21	355.16	371.76	581.44	286.55	315.37	68.33
ncRv13681cB	63.92	73.37	105.13	167.13	80.24	86.79	152.39	92.23	86.17	66.86	74.66	75.68	42.64	531.69	706.09
ncRv13687c	466.25	309.06	310.65	131.9	389.12	145.4	658.26	944.25	847.16	748.05	804.06	215.96	150.1	161.73	30.37
ncRv13709c	364.06	852.99	1123.75	1051.07	812.97	903.49	1327.64	736.95	651.94	694.3	686.14	249.56	158.97	652.58	373.54
ncRv13723	756.81	1163.37	545.33	653.26	1663.31	376.83	256.09	433.9	422.44	393.22	298.64	395.01	163.74	1022.94	75.92
ncRv13725	1219.58	738.03	434.17	260.4	895	327.01	229.61	482.53	555.41	638.23	552.35	728.49	267.97	1154.12	133.29
ncRv13739c	169.2	103.53	70.71	83.88	151.28	77.65	226.37	2756.62	2673.81	4048.24	5363.39	718.03	310.43	136.46	0

Table S4d. Context dependent expression of sRNAs

ncRv13750c	200.86	621.01	640.96	503.27	812.8	1402.28	583.85	224.9	239.3	257.74	294.13	313.79	296.78	667.14	966.76
ncRv13801c	543.69	495.73	466.73	693.95	357.02	315.55	1557.04	928.08	874.13	914.54	927.41	1418.98	1289.46	401.29	123.38
ncRv13802c	635.67	388.97	457.56	574.37	394.12	183.62	392.68	458.54	423.61	363.57	311.91	519.05	252.43	1229.15	1217.81
ncRv13803c	2294.57	2577.27	2161.3	1214.21	1801.7	434.31	187.33	3444.14	3805.56	3597.26	3304.25	1073.35	402.53	412.41	45.55
ncRv13804c	992.84	2572.28	1985.97	952.6	1751.84	716.75	163.14	1770.43	1854.67	1829.08	1600.12	1455.43	514.25	9524.89	417.58
ncRv13810	722.57	1550.04	246.62	93.55	1435.15	228.38	33.26	98.54	664.12	705.28	609.2	391.31	120.76	1170.93	30.37
ncRv13822A	947.91	933.5	782.81	214.61	607.16	244.37	184.25	1528.8	1668.32	1933.18	2367.15	460.07	261.39	836.45	157.54
ncRv13822B	1556.09	2505.18	1956.12	545.06	1459.14	677.54	354.74	2587.02	2624.73	3103.41	3968.35	542.67	266.08	1293.84	918.67
ncRv13823c	253.61	293.63	861.4	307.45	419.2	552.18	65.76	742.15	644.25	777.18	813.37	433.15	360.46	338.29	718.74
ncRv13836A	329.01	399.81	378.47	386.96	460.68	121.8	1139.14	534.18	456.14	472.99	345.65	252.88	332.6	242.6	53.15
ncRv13836B	516.31	393.93	447.66	459.93	514.36	149.97	1670.62	776.51	721.78	759.3	548.43	636.81	880.11	564.03	318.88
ncRv13846	547.28	455.1	273.92	171.41	675.41	475.04	150.22	298.44	291.84	305.56	246.58	723.04	306.53	492.7	264.65
ncRv13847	176.31	212.99	76.78	57.12	170.07	246.96	431.12	455.07	369.83	336.29	336.37	756.05	480.31	617	145.77
ncRv13848	289.22	541.17	313.36	144.37	535.14	215.44	225.74	943.01	1474.34	1585.47	1376.76	878.61	371.83	287.07	83.52
ncRv13849	708.98	1163.12	679.85	340.92	1216.92	382.67	177.9	649.51	790.03	776.52	635.58	670.65	330.89	439.37	141.72
ncRv13855A	524.81	299.34	306.92	270.97	195.48	57.1	786.3	1281.28	1232.99	1434.53	1610.88	649.73	460.52	679.27	288.51
ncRv13855B	519.44	367.34	301.83	351.11	241.79	78.41	815.08	1061.55	1014.32	1151.53	1332.21	690.34	533.86	473.06	440.35
ncRv13859c	415.58	136	125.93	116.2	153.47	222.29	275.59	466.19	498.79	546.14	461.07	554.36	428.11	162.4	698.49
ncRv13870	958.87	787.09	785.77	463.36	771.67	376.07	951.03	2386.78	1709.22	1690.72	1882.48	930.3	1367.92	8551.47	2915.45
ncRv13871	578.75	727.69	651.59	510.76	896.81	478.08	1605.49	4160.56	2589.96	2883.19	2846.51	3460.31	2909.82	1536.44	450.48
ncRv13875	4269.97	7085.39	3981.66	3267.42	11503.7	5592.36	589.54	3204.24	2612.2	2406.34	2090.07	3006.23	3378.3	3060.74	1898.08
ncRv13879cA	490.38	162.33	119.04	133.15	182.06	58.62	352.21	761.65	523.2	451.81	407.1	673.73	866.46	456.89	204.99
ncRv13879cB	468.48	223.68	156.34	179.61	248.36	92.88	340.83	728.54	483.35	419.7	377.91	670.03	874.99	155.67	53.15
ncRv13879cC	462.67	392.9	337.44	312.13	489.89	187.27	436.62	1160.89	854.28	759.63	629.85	492.84	492.93	111.19	83.52
ncRv13901c	91.64	88.7	56.3	35.24	61.83	19.79	275.7	189.72	162.63	153.91	123.51	125.52	78.46	1469.72	372.02
ncRv13907c	433.91	189.34	153.29	134.5	150.67	72.58	470.03	1378.26	1228.89	1212.33	1185.96	331.02	125.08	229.12	65.8
ncRv13916cA	365.37	341.78	412.05	604.82	260.82	177.38	1385.75	951.47	620.28	543.38	508.9	596.2	943.79	506.08	217.65
ncRv13916cB	1296.37	469.85	393.73	451.2	371.76	143.88	7745.11	3640.21	2597.51	2414.61	2152.55	2220.53	3274.82	1874.05	599.79
ncRv13919c	796.3	1432.38	804.43	440.91	741.94	75.62	896.44	1911.4	1296.27	1114.35	1092.15	305.18	146.68	1695.47	50.62

Table S4d. Context dependent expression of sRNAs
