# Supplementary material for: Genome-wide identification of the context-dependent sRNA expression in Mycobacterium tuberculosis
Source: BMC Genomics. 2020 Feb 18;21:167. doi: 10.1186/s12864-020-6573-5 (PMC7029489; doi:10.1186/s12864-020-6573-5)
Supplement: Supplementary file 11 — Additional file 11 Table S5. Target genes of some of the sRNAs. Predicted gene targets of the sRNAs along with their quantified expression in relevant growth conditions. Targets of (a) ncRv11806, (b) ncRv11875 and (c) ncRv11706A. [file 12864_2020_6573_MOESM11_ESM.pdf]

Table S5a. Predicted targets of ncRv11806					
				Expression (RPKM)	
**Target genes**	**Binding energy**	**p-value**	**Protein**	**Exponential phase**	**Stationary phase**
nrdH	-29.3389	6.54E-05	glutaredoxin electron transport protein NrdH	864.477	363.733
rpfB	-27.8514	0.0001727	resuscitation-promoting factor RpfB	249.916	8.723
Rv2619c	-25.7173	0.0006211	hypothetical protein	130.862	43.016
Rv2844	-25.2943	0.0007894	hypothetical protein	236.547	17.114
Rv2275	-24.7601	0.0010621	cyclo(L-tyrosyl-L-tyrosyl) synthase	176.456	1.747
Rv2675c	-24.6502	0.001128	hypothetical protein	131.728	68.149
thyX	-24.2508	0.0014008	thymidylate synthase ThyX	26.48	1.514
senX3	-24.096	0.0015221	two component sensor histidine kinase SenX3	472.998	61.318
Rv2985	-23.6946	0.0018831	8-oxo-dGTP diphosphatase	348.216	17.527
psiH2	-23.2559	0.0023672	phosphate starvation-inducible protein PsiH	527.394	32.388

Table S5b. Predicted targets of ncRv11875							
				Expression (RPKM)			
**Gene**	**Binding energy**	**p-value**	**Protein**	**Exponential phase**	**High iron**	**Low iron day 1**	**Low iron week 1**
PE_PGRS6	-25.4358	0.0006287	PE-PGRS family protein PE_PGRS6	131.025	134.881	125.013	83.403
Rv1924c	-24.92	0.0008343	hypothetical protein	186.926	276.197	249.151	123.69
Rv1526c	-24.7125	0.0009335	glycosyltransferase	32.758	52.444	23.93	13.831
Rv1728c	-24.643	0.0009692	hypothetical protein	137.997	186.725	61.808	34.467
ilvB1	-21.67	0	acetolactate synthase large subunit IlvB	321.238	277.897	86.883	76.876
Rv1937	-19.21	0	oxygenase	19.725	47.257	41.907	92.708
atpA	-18.17	0	ATP synthase subunit alpha	1020.902	596.961	357.539	294.057
Rv0544c	-15.74	0	transmembrane protein	626.959	942.754	730.304	380.442
Rv1626	-14.99	0.001	two-component system transcriptional regulator	592.65	354.751	194.066	128.827
nuoF	-8.59	0.044	NADH-quinone oxidoreductase subunit F	316.344	494.585	85.014	55.005

Table S5c. Predicted targets of ncRv11706A									
				Expression (RPKM)					
**Target genes**	**Binding energy**	**p-value**	**Protein**	**Exponential phase**	**Persistence**	**Reactivation day 1**	**Reactivation day 2**	**Reactivation day 3**	**Reactivation day 4**
Rv3047c	-19.5889	4.06E-05	hypothetical protein	55.091	195.261	127.674	82.305	100.185	104.207
esxT	-18.22	0	ESAT-6 like protein EsxT	36.487	27.673	76.154	44.937	44.651	39.303
Rv1382	-17.94	0	hypothetical protein	72.45	15.887	51.499	46.342	33.644	32.374
Rv1490	-15.11	0.001	membrane protein	32.885	41.934	52.824	42.811	42.369	45.004
Rv2898c	-15.0063	0.0012578	hypothetical protein	53.214	39.355	134.058	96.491	79.301	83.367
ftsE	-14.1393	0.0022508	cell division ATP-binding protein FtsE	132.258	249.564	119.722	119.112	95.638	84.911
Rv2325c	-11.9418	0.0090732	hypothetical protein	143.517	147.45	329.987	290.941	265.558	273.099
recX	-11.6571	0.010782	regulatory protein RecX	249.531	28.197	158.659	370.101	239.412	221.786
vapB27	-11.52	0.01	antitoxin VapB27	400.711	218.15	211.012	255.232	255.408	309.815
nuoN	-9.44	0.031	NADH-quinone oxidoreductase subunit N	273.077	126.101	300.998	284.081	269.185	372.02
